# Heck Diversification of Indole‐Based Substrates under Aqueous Conditions: From Indoles to Unprotected Halo‐tryptophans and Halo‐tryptophans in Natural Product Derivatives

**DOI:** 10.1002/chem.201901327

**Published:** 2019-07-19

**Authors:** Cristina Pubill‐Ulldemolins, Sunil V. Sharma, Christopher Cartmell, Jinlian Zhao, Paco Cárdenas, Rebecca J. M. Goss

**Affiliations:** ^1^ Department of Chemistry and BSRC University of St Andrews St Andrews KY16 9ST UK; ^2^ Present address: Department of Chemistry School of Life Sciences University of Sussex Brighton BN19QJ UK; ^3^ Pharmacognosy, Department of Medicinal Chemistry Uppsala University Uppsala 75123 Sweden

**Keywords:** aqueous cross-coupling, barettin, Heck reaction, natural product modification, halo-tryptophan

## Abstract

The blending of synthetic chemistry with biosynthetic processes provides a powerful approach to synthesis. Biosynthetic halogenation and synthetic cross‐coupling have great potential to be used together, for small molecule generation, access to natural product analogues and as a tool for chemical biology. However, to enable enhanced generality of this approach, further synthetic tools are needed. Though considerable research has been invested in the diversification of phenylalanine and tyrosine, functionalisation of tryptophans thorough cross‐coupling has been largely neglected. Tryptophan is a key residue in many biologically active natural products and peptides; in proteins it is key to fluorescence and dominates protein folding. To this end, we have explored the Heck cross‐coupling of halo‐indoles and halo‐tryptophans in water, showing broad reaction scope. We have demonstrated the ability to use this methodology in the functionalisation of a brominated antibiotic (bromo‐pacidamycin), as well as a marine sponge metabolite, barettin.

## Introduction

Tryptophan is a key residue in many biologically active natural products, peptides and proteins.^1^, ^2^ Its intrinsic fluorescence dominates the spectrophotometric properties of a given peptide or protein; it is a crucial residue for stabilising secondary and tertiary structure through intra and intermolecular interactions.^3^ Tryptophan residues have been shown to play a central role in protein folding^4^ as well as being implicated in governing the function of many biologically important systems including mechanosensitive channels within the membrane.^5^ The possibility of functionalising tryptophan in order to modify this important residue both sterically and electronically would be exciting and potentially afford a means of interrogating, modulating and tuning the properties of peptides, proteins and natural products.

Though considerable research has been carried out on selective modification of halogenated phenylalanines and tyrosines^7^ through the application of cross‐coupling chemistry, the functionalisation of tryptophan has, until recently, remained largely unexplored.^6^ One potential reason for this is the challenge that such metal mediated cross‐coupling reactions present with tryptophan; indeed, tryptophan has been demonstrated to poison the Suzuki–Miyaura cross‐coupling of halo‐indoles.^8^ This may be attributed to the amino acid coordinating to the palladium catalyst.^9^ Notably, esterification of the carboxylate and acylation of the primary amine reduces reaction poisoning, however even this species is not fully innocent and its incorporation in a reaction is still seen to impact upon conversion.^8^

In recent years, series of studies enabling the cross‐coupling of halo‐tryptophans through application of Suzuki–Miyaura and Sonogashira chemistries in aqueous media have been reported.^6^, ^7^, ^8^ Combination of these chemistries with enzymatic halogenation has been used powerfully for selective C−H activation and diversification of small molecules^10^ The application of Heck cross‐coupling would provide a valuable addition to this growing portfolio of reactions for tryptophan functionalisation, enabling the potential for extension of conjugation and tuning of electronic and fluorescence properties as well as the opportunity to potentially modulate conformations of small molecules, peptides and proteins.

Heck cross‐coupling methodologies have been effectively applied to series of other biomolecules including nucleosides,^11^ nucleotides and nucleoside triphosphates.^12^ However, there are very few studies reported of the utilisation of Heck methodologies for the modification of amino acids, and peptides, mostly utilising highly activated (iodo or triflate) substrates. These include modification of N‐ and C‐protected 3,5‐di‐iodo‐l‐tyrosine, modulating and extending its conjugation and enabling its fluorescence properties to be tuned, and diversification of N‐ and C‐protected l‐tyrosine *para*‐triflate,^13^ and selective modification of 4‐iodo‐l‐phenylalanine within a small protein, using Mizoroki–Heck conditions.^14^

**Scheme 1:**
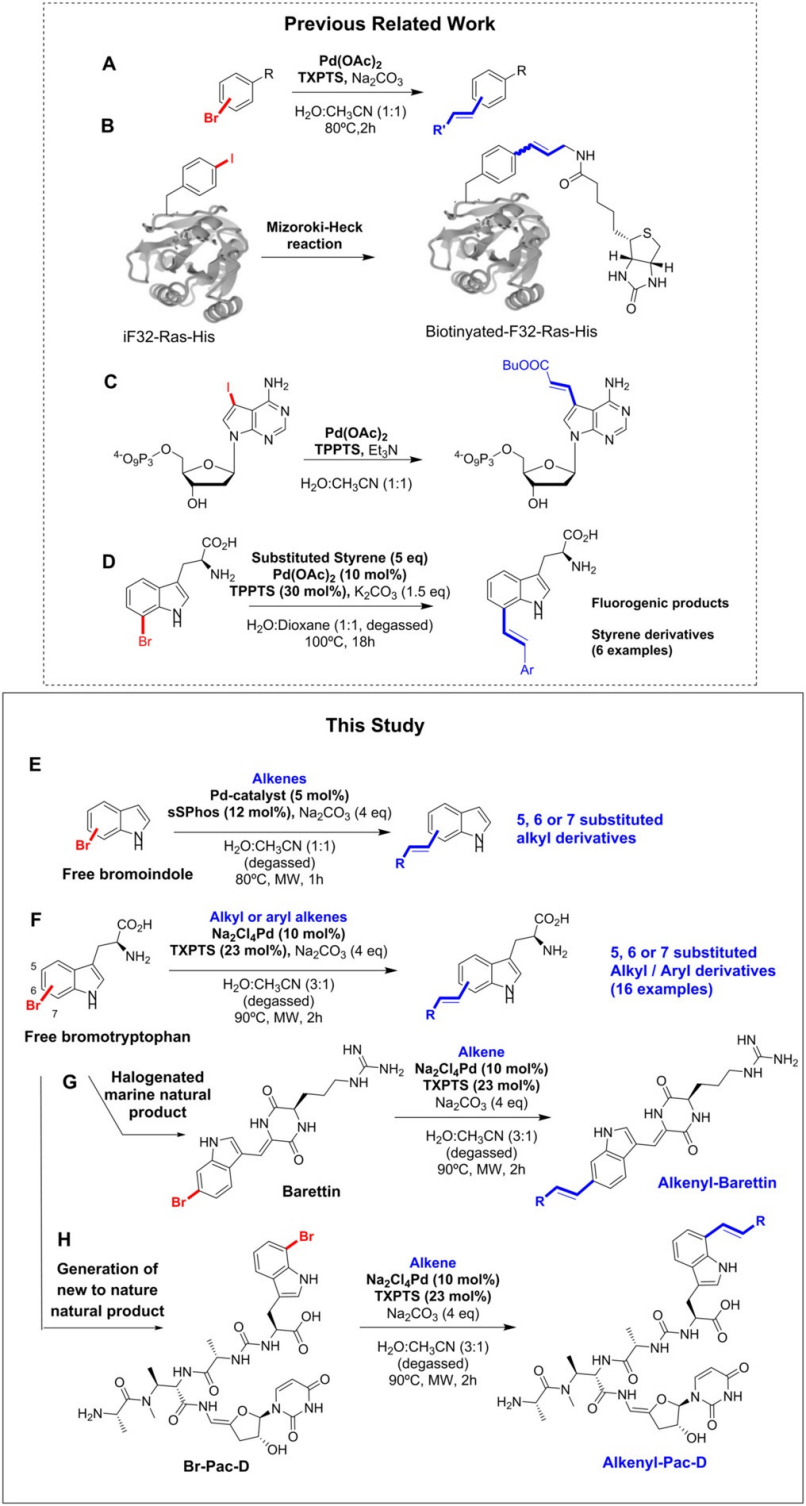
Heck cross‐coupling under aqueous conditions applied to biomolecules: all relevant prior work is outlined along with work within this study. (A) Shaughnessy's initial aqueous Heck cross‐coupling.^16^ (B) Tagging of 4‐iodo‐l‐phenylalanine within a small protein.^14^ (C) Heck modification of iodinated nucleoside triphosphates.^12^, ^13^ (D) Reported Heck modification of 7‐bromo‐tryptophan with styrenes.^15^ (E), (F) Heck modification of a series of indoles and tryptophans, respectively. (G) and (H) Heck modification of the antibiotics barettin and pacidamycin, respectively.

Whilst this manuscript was being prepared, the first Mizoroki–Heck coupling of a halo‐tryptophan was reported. In this study free, unprotected 7‐bromo‐tryptophan was derivatised with 6 different styrenes.^15^ Excitingly, as observed with the Suzuki–Miyaura cross‐coupling of tryptophan,6a, 8 these tryptophan‐7‐styrene products were shown to be fluorogenic, thus again opening up the way for fluorescence modulation of halo‐tryptophans. There is considerable potential for the development of Heck methodologies for the functionalisation of free and biomolecule embedded halo‐tryptophans. Herein, we report our exploration of the systematic application of the Heck reaction first to halo‐indoles, then to free and unprotected halo‐tryptophans. We then move on to challenge the developed methodology, applying it to unprotected and complex natural product barettin as well as natural product derivative bromo‐pacidamycin and to a range of aliphatic and aromatic alkenes (Scheme 1).

## Results and Discussion

Moore and Shaughnessy first exemplified the use of aqueous phase Heck coupling of aryl bromides using the sterically demanding tri‐(4,6‐dimethyl‐3‐sulphonatophenyl)phosphine trisodium salt (TXPTS) as ligand to enable water solubilisation of the Pd(OAc)_2_. Their mild aqueous conditions, using Na_2_CO_3_ as base enabled modification of aryl iodides and bromides in high yield (79–94 %).^16^ Starting with their reported conditions, we explored whether these could be utilised to enable Heck modification of reactive 5‐iodo‐indole **1** with acrylic acid **2**. By heating for 18 h using Na_2_PdCl_4_‐TXPTS as the catalyst gave a conversion to 51 % and an isolated yield of 43 % (Table 1).

**Table 1:** Initial Heck cross‐coupling conditions explored for 5‐I‐indole with acrylic acid.

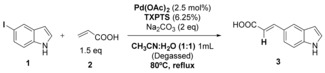					
Entry	X	Pd/Ligand	*t* [h]	Conv. [%]^[a,b]^	Yield [%]^[c]^
1	5‐I **1**	Pd(OAc)_2_/TXPTS	2	18	–
2	5‐I **1**	Pd(OAc)_2_/TXPTS	18	32	–
3	5‐I **1**	Na_2_PdCl_4_/TXPTS	18	51	43

Reaction conditions: [a] 5‐I‐indole (0.1 mmol), acrylic acid (1.5 equiv, 0.15 mmol), Pd salt (2.5 mol %), TXPTS (6.25 mol %), Na_2_CO_3_ (2.0 equiv, 0.2 mmol), CH_3_CN/H_2_O (1:1, 1 mL), conventional heating, reflux, solids and solvents purged with argon. [b] Based on ratio of starting material aromatic peak at *δ*_H_ 6.40 ppm compared to product aromatic peak at *δ*_H_ 6.50 ppm in CD_3_OD. [c] Isolated yields are reported after flash chromatography.

To improve these results, we next explored the impact of varying the catalyst, (investigating water‐soluble Na_2_PdCl_4_) in combination with exploring the application of the sterically more demanding and electron‐rich ligand ^S^SPhos, designed by Buchwald.^17^ Microwave heating was also explored (Table 2).

**Table 2:** Optimization of the reaction conditions.

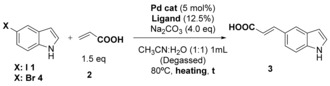						
Entry	X	Pd/Ligand	*T*	*t* [h]	Conv. [%]^[a,b]^	Yield [%]^[c]^
1	5‐I **1**	Pd(OAc)_2_/TXPTS	reflux	18	78	67
2	5‐I **1**	Pd(OAc)_2_/^s^SPhos	reflux	18	85	73
3	5‐I **1**	Na_2_PdCl_4_/^s^SPhos	reflux	18	97	86
4	5‐I **1**	Na_2_PdCl_4_/^s^SPhos	MW	1	>99	90
5	5‐I **1**	Na_2_PdCl_4_/none	MW	1	>99	89
6	5‐Br **4**	Na_2_PdCl_4_/none	MW	1	48	36
7	5‐Br **4**	Na_2_PdCl_4_/TXPTS	MW	1	77	65
8	5‐Br **4**	Na_2_PdCl_4_/^s^SPhos	MW	1	>99	94

Reaction conditions: [a] 5‐X‐indole (0.1 mmol), acrylic acid (1.5 equiv, 0.15 mmol), Pd catalyst (5 mol %), ligand (12.5 mol %), Na_2_CO_3_ (4 equiv, 0.4 mmol), CH_3_CN/H_2_O (1:1, 1 mL), conventional or microwave heating (*T*=80 °C), solids and solvents purged with argon. [b] Based on ratio of starting material aromatic peak at *δ*_H_ 6.40 ppm compared to product aromatic peak at *δ*_H_ 6.50 ppm in CD_3_OD. [c] Isolated yields are reported after flash chromatography.

These studies revealed that by using ^S^SPhos in place of TXPTS gave a modest increase in conversion of 5‐iodo‐indole **1** (78–85 %, Table 2, entries 1 and 2). A significant increase in conversion (97 %) could be seen upon replacing Pd(OAc)_2_ with Na_2_PdCl_4_ (Table 2, entry 3). By replacing conventional heating with microwave heating, >99 % conversion could be achieved after only one hour. Notably, for 5‐iodo‐indole **1**, it was possible to achieve almost quantitative conversion using microwave heating and in the absence of an additional ligand (Table 2, entry 5). Next, we set out to explore the Heck modification of the less reactive 5‐bromo‐indole **4**. Here, we observed conversions to be far more modest in the absence of any ligand, however utilisation of ^S^SPhos again enabled almost quantitative conversions (Table 2, entry 8).

With these conditions in hand, we next set out to explore whether we could proceed past acrylic acid **2** as the coupling partner. The conditions that we had developed showed good applicability enabling the Heck modification of both 5‐iodo **1** and 5‐bromo‐indole **4** to proceed almost quantitatively with a range of different alkene cross‐coupling partners (Table 3).

**Table 3:** Heck cross‐coupling of 5‐X‐indoles with different alkenes using Na_2_PdCl_4_/^s^SPhos as catalytic system.

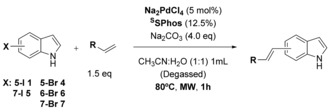					
Entry	X	R	Product	Conv. [%]^[a,b]^	Yield [%]^[c]^
1	5‐I **1**	CO_2_Et **8**	**13**	>99	60^[d]^
2	5‐I **1**	COOH **2**	**3**	>99	92
3	7‐I **5**	COOH **2**	**14**	>99	91
4	5‐I **1**	COMe **9**	**15**	>99	85
5	5‐I **1**	CN **10**	**16**	>99	75
6	5‐I **1**	Ph **11**	**17**	33	–
7	5‐Br **4**	CO_2_Et **12**	**13**	>99	71^[d]^
8	5‐Br **4**	COOH **2**	**3**	>99	94
9	6‐Br **6**	COOH **2**	**18**	>99	83
10	7‐Br **7**	COOH **2**	**14**	>99	89

Reaction conditions: [a] 5‐X‐indole (0.1 mmol), alkene (1.5 equiv, 0.15 mmol) Na_2_PdCl_4_ (5 mol %), ^s^SPhos (15 mol %), Na_2_CO_3_ (4.0 equiv, 0.4 mmol), CH_3_CN/H_2_O (1:1, 1 mL), MW heating used, solids and solvents purged with argon. [b] Based on ratio of starting material aromatic peak at *δ*_H_ 6.40 ppm compared to product aromatic peak at *δ*_H_ 6.50 ppm in CD_3_OD. [c] Isolated yields are reported after flash chromatography. [d] Desired product formed alongside acrylic acid derivative due to hydrolysis of the ethyl ester under basic conditions. Yields are reported for the ethyl acrylate product only.

The only exception to this rule was styrene **11** (Table 3, entry 6) because of the reduced reactivity of this species arising due to the electron rich nature of the alkene. Also, for this reason, nonactivated 1‐octene and cyclohexene gave no conversion under the same reaction conditions. From NMR characterisation of the products it may be seen that all Heck coupling reactions progressed with a high level of stereoselectivity to generate only the *E*‐isomers. The *trans*‐relation of the double bonds was established on the basis of the coupling constant for the vinylic protons in the ^1^H NMR spectra (*J*=16 Hz, see Supporting Information).

Having achieved a system that would work well for the functionalisation of iodo‐ and bromo‐indoles **1** and **4**, we next set out to explore whether it might be possible to extend this methodology further to the aqueous cross‐coupling of the far more challenging free, unprotected halo‐tryptophans.

Halo‐tryptophans may be readily accessed through a simple one‐step biotransformation using tryptophan synthase,^18^, ^19^ or through a 4–5 step chemical synthesis.^20^ Challenges that need to be addressed to render Heck cross‐coupling of free halo‐tryptophans useful are their poor solubility and their propensity to chelate to and deactivate the palladium catalyst.^9^

Utilisation of our previous conditions that had been optimised for the aqueous Heck cross‐coupling of halo‐indoles with acrylic acid resulted in almost no observable conversion of 5‐bromo‐tryptophan **19** even at 100 °C (Table 4, entries 1 and 2), we therefore returned to exploration of both TXPTS and TPPTS as water soluble ligands with the highly reactive 7‐iodo‐tryptophan **20**. By doubling both the amount of catalyst and ligand, it was possible to achieve almost quantitative conversion using either TPPTS or TXPTS when heated to 90 °C, though with TPPTS an extended reaction time of 2 h was required (Table 4, entries 3–6). Applying these conditions, using TPPTS to the less reactive 7‐bromo‐tryptophan **21**, a conversion of only 47 % is observed (Table 4, entry 7). However, by switching to the more sterically demanding TXPTS ligand, almost quantitative conversion was achieved (Table 4, entry 8). As the 5 and 6‐halo‐tryptophans are more reactive than the 4 and 7‐counterparts, the sterically less demanding TPPTS was found suitable to afford almost quantitative conversion of 5‐bromo and 6‐bromo‐tryptophan **19** and **22** (Table 4, entries 9 and 10). Importantly, as seen for the halo‐indoles the reaction is highly stereoselective towards the *E* product (See Supporting Information).

**Table 4:** Heck cross‐coupling on unprotected halo‐tryptophans with acrylic acid.

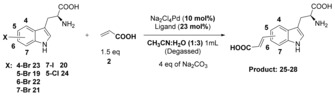							
Entry	X	Prod	Ligand	*t* [h]	*T* [°C]	Conv. [%]^[a,b]^	Yield [%]^[c]^
1	5‐Br **19**	**25**	^S^SPhos	1	80	<1	–
2	5‐Br **19**	**25**	^S^SPhos	2	100	<1	–
3	7‐I **20**	**26**	TPPTS	1	90	65	–
4	7‐I **20**	**26**	TPPTS	2	90	>99	93
5	7‐I **20**	**26**	TXPTS	1	80	24	**–**
6	7‐I **20**	**26**	TXPTS	1	90	>99	93
7	7‐Br **21**	**26**	TPPTS	2	90	47	–
8	7‐Br **21**	**26**	TXPTS	1	90	>99	87
9	5‐Br **19**	**25**	TPPTS	1	90	>99	95
10	6‐Br **22**	**27**	TPPTS	1	90	>99	–^[d]^
11	4‐Br **23**	**28**	TXPTS	1	90	<1	–
12	5‐Cl **24**	**25**	TXPTS	1	90	<1	–

Reaction conditions: [a] Halo‐tryptophan (0.05 mmol, 1.0 equiv), acrylic acid (0.075 mmol, 1.5 equiv), Na_2_PdCl_4_ (10 mol %), ligand (23 mol %), Na_2_CO_3_ (4 equiv), water/acetonitrile (3:1, 1 mL, degassed), microwave heating, 90 °C, 1 h. [b] Based on NMR ratios of starting material to product material in CD_3_OD. [c] Products purified by reverse phase chromatography with a MeOH/H_2_O gradient. [d] Product could not be isolated pure due to potential degradation through reverse‐phase column chromatography.

### Observed limitations in reactivity

The 4‐bromo‐tryptophan **23** is highly unreactive and even using TXPTS as ligand, only a small trace of product may be perceived from the reaction. Curiously, the sole application of Heck cross‐coupling to tryptophan reported in the literature is the functionalisation of 4‐bromo‐tryptophan **23** in the synthesis of clavicipitic acid (reported as 91 % conversion using conventional heating);^15^ we have been unable to reproduce this conversion using either the system that we have developed or their conditions with alkene 1,1‐dimethylallyl alcohol, TPPTS, Pd(OAc)_2_ and NaOH as base. Forcing the reaction by MW heating to 130 °C for 2 h we were finally able to obtain a conversion of around 15 %.

The less reactive aryl chloride, as a component of either the simple indole system or within tryptophan is also recalcitrant to Heck modification under all conditions that we have explored. However, we demonstrate that following protection of the primary amine (using *N*‐Boc‐4‐bromo‐tryptophan), Heck coupling at position 4 can be achieved using our conditions. A similar trend was observed for reactivity of free versus protected 2‐bromo or 4‐bromo‐phenylalanine. These observations strongly indicate influence of free α‐amino group on the Pd‐catalysed cross‐coupling of 4‐halo‐tryptophans (see Supporting Information).

**Scheme 2:**
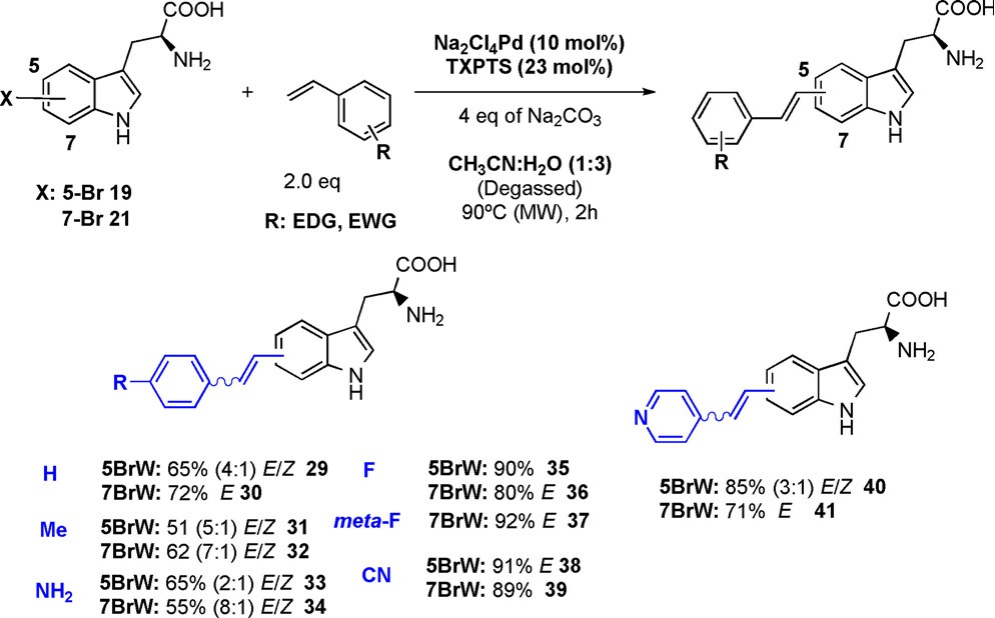
Scope of alkene partner with styrene derivatives. The impact of modification of sterics and electronics is explored.

With conditions established that would enable the conversion of 5, 6 and 7‐iodo and bromo‐tryptophans, we next set out to explore the impact of sterics and electronic and solubility of the cross‐coupling partners (Scheme 2). Through this we could observe that progression from acrylic acid to bulkier aromatic substrates could be successfully achieved. Trend in coupling efficiency seems to follow electronics rather than substrate solubility. It is evident from the results that electron donating substituents (i.e. Me or amino) decrease the reactivity of alkene resulting in lower yields (51–65 %, compounds **31**–**34**). On the contrary, electron‐withdrawing fluoro or nitrile derivatives gave significantly higher yields (80–92 %, compounds **35**–**39**), and reactions also worked well with heterocyclic alkene substrates such as 4‐vinylpyridine (compounds **40** and **41**).

From NMR characterisation of the products, it may be seen that for the 5‐bromo and 5‐iodo‐tryptophan, an almost equal mixture of the *E* and *Z* isomer are generated, whereas for the 7‐bromo and 7‐iodo‐tryptophans the *E* stereoisomers predominate (see Supporting Information).

**Scheme 3:**
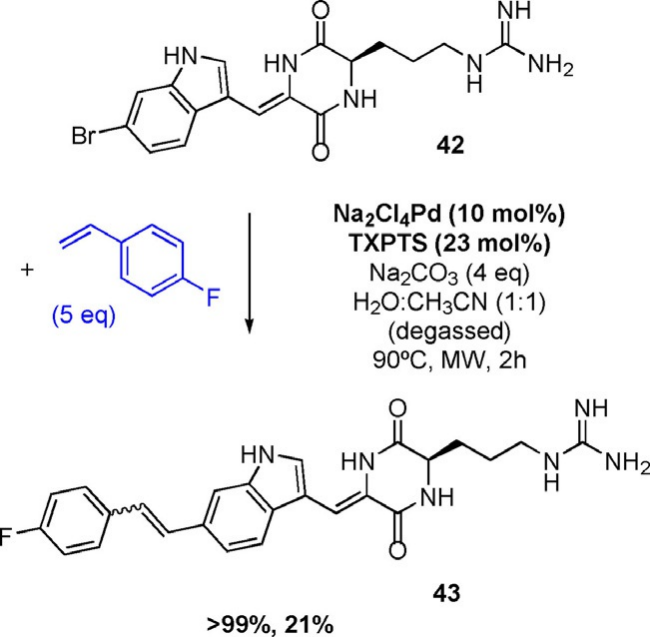
Application of Heck to the diversification of the marine natural product, barettin. Reaction carried out on a 2 mg scale.

The selective diversification of natural products is an important area. Such work can enable systematic modification and optimisation of a bioactive molecule's properties, or enable tagging and tracking, or be utilised in target identity. As proof of principle, we next set out to explore whether Heck cross‐coupling might be applied to the cross‐coupling of tryptophan residues within two test‐bed natural products. The sponge halo‐metabolite barettin, a brominated a diketopiperazine‐type cyclic dipeptide (**42**, Scheme 3), is known for its roles in chemical defence against predators, antifouling activity and binding to serotonergic 5‐HT receptors.^21^ Heck modulation could be potentially explored to enable analogue generation to gain greater understanding of the molecule's structure–activity relationship. Furthermore, Heck tagging impacts upon the fluorescence of halo‐indoles/tryptophans, such a strategy could potentially be applied to enable the tissues in which it is generated to be seen, as well as enabling tracking of the metabolite and potentially revealing its targets.

Within barettin, the primary amine and carboxylate of tryptophan, that would usually add challenge to cross‐coupling reaction progression, are masked as amides within a diketopiperazine. Many marine metabolites are highly lipophilic, limiting their diffusion from their producer, enabling the producer to retain the metabolites. To dissolve barettin, a higher ratio of acetonitrile was required. Once dissolved, the reaction proceeded well, affording product **43**.

**Scheme 4:**
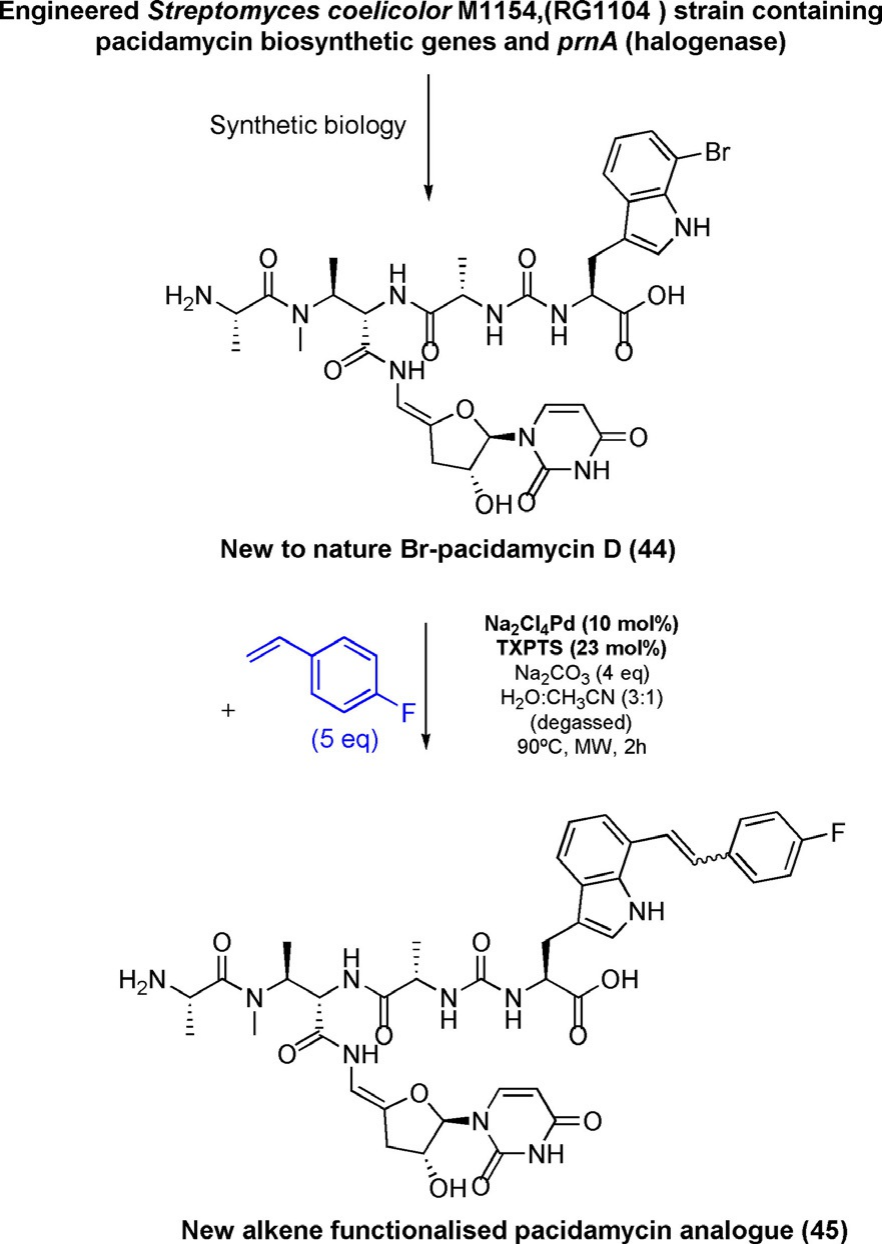
Application of Heck to the diversification of pacidamycin.

Pacidamycin, belonging to the class of uridyl peptide antibiotics, represents a potentially more challenging substrate, comprised of a pseudo‐peptide backbone attached via an exocyclic enamide to a modified uridine. We had previously demonstrated the first out of context use of a halogenase, by introducing the gene encoding tryptophan‐7‐halogenase *prnA* in *Streptomyces coeruleorubidis* (RG‐5059) in order to generate Cl‐pacidamycin.6a We successfully utilized a synthetic biological approach to generate an engineered strain (*S. coelicolor* M1154, named RG1104) capable of generating new bromometabolites, Br‐pacidamycin D (**44**) and performing Suzuki–Miyaura derivatisation to gain access to the analogue aryl‐pacidamycin D (Scheme 4).^8^

In this study, we employed the same strain in ISP2 medium for production and isolation of Br‐pacidamycin D and cultures were grown for a total of 7–8 days. Production of Br‐pacidamycin D along with wild type pacidamycin D was detected by LC‐HRMS analysis. Isolation of the target compounds followed sequence of purification steps: solid‐phase extraction (XAD‐16 resin), enrichment of pacidamycins using ion exchange chromatography (HiTrap SP‐FF columns) and reverse phase purification (semi‐preparative HPLC) (see Supporting Information for details).

In this manner, reasonably pure samples of wild‐type pacidamycin D (1 mg) and Br‐pacidamycin D (ca. 0.5 mg) were isolated. With the isolated material in hand, we could perform partial NMR analysis on these samples. While the ^1^H NMR on pacidamycin D was satisfactory, peak broadening was observed for the brominated analogue. Gratifyingly, some key differences were noted by careful comparison of the HSQC‐NMR (see Supporting Information), notably ^1^H and ^13^C peak at 7.35 and 110.7 ppm for pacidamycin D was absent in the HSQC spectrum of Br‐pacidamycin D, thus indicating substitution in desired position. LC‐HRMS^2^ analyses showed desired isotope pattern (*m*/*z* 790, 792 for ^79^Br, ^81^Br, respectively) and satisfactory MS^2^ fragmentation was obtained (see Supporting Information).

To enable the modification of bromo‐pacidamycin through application of Heck chemistry we first explored reactions on crude extracts containing very low concentrations of this bromometabolite. Using our optimum conditions developed for Br‐tryptophan, we were pleased to see full conversion of Br‐pacidamycin D from crude extract and LC‐HRMS^2^ analyses confirmed formation of the desired cross‐coupling product. Next, we carried out the Heck coupling on a purified sample of Br‐pacidamycin D (ca. 0.5 mg). Although the cross‐coupling was successful as confirmed by LC‐HRMS^2^ analysis (LC‐HRMS^2^ product *m*/*z* C_40_H_47_FN_9_O_10_^+^ [*M*+H]^+^: 832.3424; found: 832.3422), the corresponding product **45** could not be successfully isolated on this very small scale. These results indicated that our method has the potential to be utilised for micro‐scale tagging or functionalisation of brominated metabolites, even as components of a complex extract without the need for prior application of purification or protection strategies.

## Conclusions

We have developed conditions enabling Heck cross‐coupling of iodo‐ and bromo‐indoles and free unprotected 5, 6 and 7‐bromo‐tryptophan and 7‐iodo‐tryptophan, showing the reaction to be very high yielding. Near quantitative cross‐coupling of iodo‐ and bromo‐indoles can be achieved in the absence of ligand, however to enable comparable conversions of halo‐tryptophans, a water‐soluble ligand is required, and we found that TXPTS and TPPTS could be used effectively. While high conversions could be achieved for 5, 6, and 7‐iodo and bromo‐indoles and tryptophans, exploration of limitation of scope revealed the less reactive aryl chlorides to be recalcitrant to cross‐coupling under our conditions, 4‐halo species also proved demanding.

We have demonstrated the ability to carry out Heck modification of halo‐tryptophan with a wide range of alkenyl cross‐coupling partners. Introducing this chemistry to more sensitive and complex systems in which halo‐tryptophans are embedded, we have used synthetic biology to engineer the production of the halo‐metabolite, bromo‐pacidamycin, and have demonstrated the selective modification of this polar antibiotic, even as a component of a complex extract. We have also demonstrated the application of these conditions to the modification of barettin, a natural and lipophilic metabolite obtained from a sponge.

The fairly mild aqueous conditions, the high conversions and flexibility of the substrate scope, make the Heck reaction a useful tool for application to chemical biology and molecule tagging as well as to GenoChemetic approaches to natural product analogue generation. Tryptophan is an important residue in natural products, peptides and proteins with a key role in folding, function and fluorescence. The extension and modulation of the conjugation enabled by Heck cross‐coupling with series of alkene partners provides the potential for tuning the conformation and modulating fluorescence properties.

## Experimental Section

Proton (^1^H), and carbon NMR (^13^C) were recorded on either a Bruker Ascend HD700 (700 MHz), Bruker Ascend 500 (500 MHz) or a Bruker 400 UltraShield (400 MHz) spectrometer. The NMR experiments were carried out in deuterated chloroform (CDCl_3_) deuterated water (D_2_O), deuterated DMSO ([D_6_]DMSO) or deuterated methanol ([D_4_]MeOH). The chemical shifts (*δ*) are quoted in parts per million (ppm). Using a DEPTQ sequence or an HSQC experiment with multiplicity editing, the ^13^C NMR signals were identified to CH_3_, CH_2_, CH and C. Coupling constants are reported in Hertz (Hz).

High‐ and low‐resolution mass spectra that were recorded at the University of St. Andrews on an Orbitrap VELOS pro. Freeze drying was carried out on a Scanvac CoolSafe freeze dryer. Microwave reactions were performed in sealed vials using a Biotage Initiator^+^ microwave reactor. UPLC analysis was acquired on a Waters Acquity H‐Class UPLC system fitted with a Waters Acquity UPLC BEH C18 column (1.7 μm, 2.1×50 mm) or Phenomenex Kinetex Phenyl‐hexyl column (2.1 μm, 2.1×75 mm).

Flash chromatography was performed using Davisil silica gel LC60A (40–63 micron). Thin layer chromatography (TLC) was executed using aluminium sheets of silica gel 60 F254 and was visualised under a Mineralight model UVGL‐58 lamp (254 nm). The plates were developed with ninhydrin in acetone or basic potassium permanganate solutions. Purification of unprotected tryptophan derivatives and peptides was carried out on a Biotage Isolera Four using reverse‐phase SNAP C18 12 g column cartridges. The purification was carried out using water (solvent A) and methanol/acetonitrile (solvent B) using the following gradient: 0–1.5 min (5 % B), 1.5–3.0 min (5 % to 15 % B), 3.0–5.0 min (15 % B), 5.0–15.0 min (15 % to 95 % B), 15.0–18.0 min (95 % B), 18.0–20.0 min (95 %‐5 % B), 20.0–25.0 min (5 % B) at a flow rate of 12–15 mL min^−1^.

Preparative RP‐HPLC purification was performed using a Gilson 322 pump, 151 UV/Vis detector and 233XL fraction collector, using a Phenomenex Luna C18 (5 micron, 250×21.2 mm) with UV detection at 234 nm. Elution was carried out using a shallow linear gradient with starting conditions 95 % solvent A (0.1 % formic acid in MQ water) to 5 % solvent B (ACN) to 40 % solvent B over 40 min.

Experimental details and characterisation data *N*‐Boc protection of 4‐Br‐(*S*)‐tryptophan, it's subsequent Heck coupling and deprotection affording compound **28**, as well as Heck reaction for *N*‐Boc‐4‐Br‐phenylalanine and *N*‐Boc‐2‐Br‐phenylalanine are presented in the Supporting Information.

**General protocol for Heck cross‐coupling of halo‐indoles with alkenes in aqueous conditions**: In a 10 mL pear‐shape flask or 0.5‐2 mL MW vial, sodium tetrachloropalladate (1.6 mg, 5 μmol, 5 mol %), sulfonated SPhos (6.6 mg, 12.5 μmol, 12.5 mol %) were purged with nitrogen and stirred at RT for 15 min after adding 1 mL of degassed water/acetonitrile (1:1) mixture. Then, appropriate halo‐indole (0.1 mmol, 1.0 equiv) is added together with Na_2_CO_3_ (22 mg, 0.2 mmol, 2 equiv) followed by addition of the alkene (0.15 mmol, 1.5 equiv). The reaction mixture was heated at 80 °C (MW or conventional heating) for the required period of time. The reaction mixture was cooled to RT and diluted with 5 mL of a saturated solution of NaHCO_3_. The aqueous layer was extracted with ethyl acetate (3×10 mL). The combined organic layers were dried over anhydrous Na_2_SO_4_ and the solvent removed in vacuo. Purification by column chromatography using silica gel (Hexanes/ethyl acetate 4:1). Characterisation data for isolated indole‐Heck products (**3**, **13**–**18**) are presented in the Supporting Information.

**General protocol for Heck cross‐coupling of unprotected halo‐tryptophans with acrylic acid in aqueous conditions**: In a 0.5‐2 mL MW vial, sodium tetrachloropalladate (1.5 mg, 5 μmol, 10 mol %), with appropriate ligand (TPPTS 6.5 mg or TXPTS: 7.0 mg, 11.5 μmol, 23 mol %) were purged with argon and stirred at RT for 15 min after adding 1 mL of degassed water/acetonitrile (3:1) mixture, 1 mL. Then, the corresponding halo‐tryptophan (0.05 mmol) was added together with Na_2_CO_3_ (22 mg, 0.2 mmol, 4 equiv) followed by addition of the acrylic acid (6 μl, 0.075 mmol, 1.5 equiv). The vial was closed, and the reaction mixture was stirred and heated at 90 °C (MW) for 1 hour. After completion, the reaction was cooled down to room temperature was diluted with water (2 mL) and acidified (pH=2–3) using 0.1 m HCl. Solvent was removed under reduced pressure. The desired product was obtained by purification using gradient reversed phase chromatography (C18, 12 g) eluting with MeOH–water (5–95 % gradient).

**(*E*)‐3‐(3‐((*S*)‐2‐amino‐2‐carboxyethyl)‐1*H*‐indol‐5‐yl)acrylic acid (25)**: Using TPPTS as a ligand, the above procedure afforded 13 mg (95 % from 5‐Br‐tryptophan) of the title product as a white solid.

^**1**^ **H NMR (400 MHz, MeOD)**: *δ*=8.01 (s, 1 H, ArH), 7.84 (d, *J*=15.9 Hz, 1 H, CH=C*H*‐Ar), 7.51–7.33 (m, 2 H, ArH), 7.25 (s, 1 H, ArH), 6.46 (d, *J*=15.9 Hz, 1 H, CH=CH‐Ar), 3.89 (dd, *J*=9.1, 4.1 Hz, 1 H, CH), 3.52 (dd, *J*=15.0, 4.5 Hz, 1 H, C*H*_A_H_B_), 3.19 ppm (dd, *J*=15.3, 9.1 Hz, 1 H, CH_A_ *H*_B_). ^**13**^ **C NMR (126 MHz, [D_6_]DMSO)** *δ*=170.1 (CO), 168.3 (CO‐CH=CH), 146.1 (CO‐CH=C*H*‐Ar), 137.6 (C), 127.7 (C), 125.6 (CH), 124.9 (C), 121.1 (CH), 120.3 (CH), 115.3 (CO‐*CH*=*C*H‐Ar), 111.8 (CH), 110.6 (C), 54.7 (CH), 26.9 ppm (CH_2_). **HRMS (FTMS ‐p NSI)** C_14_H_13_N_2_O_4_ [*M*−H]^−^ calculated for 273.0881, found 273.0880.

**(*E*)‐3‐(3‐((*S*)‐2‐amino‐2‐carboxyethyl)‐1*H*‐indol‐7‐yl)acrylic acid (26)**: The above procedure afforded 11.9 mg (87 % from 7‐Br‐tryptophan) and 12.7 mg (93 % from 7‐I‐tryptophan) of the desired product as a white solid.

^**1**^ **H NMR (400 MHz, MeOD)** *δ*=8.10 (d, *J*=16.0 Hz, 1 H, CH=C*H*‐Ar), 7.80 (d, *J*=7.5 Hz, 1 H, ArH), 7.47 (d, *J*=7.4 Hz, 1 H, ArH), 7.29 (s, 1 H, ArH), 7.13 (t, *J*=7.7 Hz, 1 H, ArH), 6.57 (d, *J*=16.0 Hz, 1 H, CH=CH‐Ar), 3.87 (dd, *J*=9.1, 4.1 Hz, 1 H, CH), 3.52 (dd, *J*=15.3, 4.0 Hz, 1 H, C*H*_A_H_B_), 3.20 ppm (dd, *J*=15.3, 9.1 Hz, 1 H, CH_A_ *H*_B_). ^**13**^ **C NMR (101 MHz, MeOD)** *δ*=174.2 (CO), 171.0 (CO‐CH=CH), 142.2 (CO‐CH=C*H*‐Ar), 136.7 (C), 129.8 (C), 126.2 (CH), 122.5 (CH), 122.1 (CH), 120.6 (CH), 119.9 (C), 119.3(CO‐*CH*=*C*H‐Ar), 110.6 (C), 56.6 (CH), 28.2 ppm (CH_2_). **HRMS (FTMS ‐p NSI)** C_14_H_13_N_2_O_4_ [*M*−H]^−^ calculated for 273.0881, found 273.0884.

**(*E*)‐3‐(3‐((*S*)‐2‐amino‐2‐carboxyethyl)‐1*H*‐indol‐6‐yl)acrylic acid (27)**: Using TPPTS as a ligand, after completion, the reaction was cooled down to RT was diluted with water (2 mL) and acidified (pH=2–3) using 0.1 m HCl. Solvent was removed under reduced pressure. ^1^NMR analysis showed clearly full conversion (>99 %) towards the title product. Unfortunately, our attempts to purify the product by reverse phase chromatography failed as the product seems to degrade through C18 even at neutral pH (7). ^1^H NMR extracted from the mixture of final product and partially oxidized TXPTS.

^**1**^ **H NMR (500 MHz, MeOD)** *δ*=7.78–7.75 (m, 2 H, overlapping ArH, CH=C), 7.60 (s, 1 H, ArH), 7.38 (dd, *J*=8.4, 1.4 Hz, 1 H, ArH), 7.33 (s, 1 H, ArH), 6.46 (d, *J*=15.9 Hz, 1 H, C=CH), 3.87 (dd, *J*=9.1, 4.2 Hz, 1 H, CH), 3.52 (dd, *J*=15.2, 4.1 Hz,1 H, C*H*_A_H_B_), 3.20 ppm (dd, *J*=15.2, 9.1 Hz, 1 H, CH_A_ *H*_B_). ^**13**^ **C NMR (126 MHz, MeOD)** *δ*=172.6 (CO), 169.8 (CO)*, 146.5 (CH), 137.0 (C), 129.0 (C), 128.2 (C), 126.3 (CH), 118.4 (CH), 118.5 (CH), 115.5 (CH), 112.2 (CH), 108.7 (C), 55.3 (CH), 26.8 ppm (CH_2_).* Identified from HMBC spectrum. **HRMS (FTMS ‐p NSI)** C_14_H_13_N_2_O_4_ [*M*−H]^−^ calculated for 273.0881, found 273.0871.

**General protocol for Heck cross‐coupling of unprotected halo‐tryptophans with styrene derivatives in aqueous conditions**. In a 0.5‐2 mL MW vial, sodium tetrachloropalladate (1.5 mg, 10 μmol, 10 mol %), TXPTS (7.0 mg, 23 μmol, 23 mol %) were purged with argon and stirred at RT for 15 min after adding 1 mL of degassed water/acetonitrile (3:1) mixture. Then, appropriate halo‐tryptophan (0.05 mmol) was added together with Na_2_CO_3_ (22 mg, 0.2 mmol, 4 equiv) followed by addition of the styrene derivative coupling partner (0.075 mmol, 1.5 equiv). The vial was closed and the reaction mixture was stirred and heated at 90 °C (MW) for 2 hours. After completion, the reaction cooled down to RT was diluted with water (2 mL) and extracted with diethyl ether (3×2 mL) to remove the excess of the alkene coupling partner. The aqueous layer was acidified (pH=2–3) using 0.1 m HCl. Solvent was removed under reduced pressure. The desired product was obtained by purification using gradient reversed phase chromatography (C‐18, 12 g) eluting with water‐MeOH (5–95 % gradient).

**(*S*)‐2‐amino‐3‐(5‐styryl‐1*H*‐indol‐3‐yl)propanoic acid (29)**: The above procedure afforded 10.9 mg (72 % from 5‐Br‐tryptophan) of the desired product as a white solid. LC‐MS analysis indicated the presence of separable *E* and *Z* isomers (4:1).

^**1**^ **H NMR (500 MHz, MeOD)** *δ*=7.89 (s, 1 H, CH), 7.58–7.50 (m, 2 H, CH=C*H*), 7.43 (dd, *J*=8.5, 1.4 Hz, 1 H, ArH), 7.39–7.27 (m, 4 H, ArH), 7.23–7.11 (m, 3 H, ArH), 3.95 (dd, *J*=9.4, 4.3 Hz, 1 H, CH), 3.55 (dd, *J*=15.6, 4.3 Hz, 1 H, C*H*_A_H_B_), 3.20 ppm (dd, *J*=15.6, 9.4 Hz, 1 H, CH_A_ *H*_B_). ^**13**^ **C NMR (126 MHz, MeOD)** *δ*=174.4 (CO), 139.6 (C), 138.3 (C), 131.3 (CH), 130.3 (C), 129.8 (2CH), 128.8 (C), 127.8 (CH), 127.1 (2CH), 126.6 (CH), 125.9 (CH), 121.6 (CH), 118.2 (CH), 112.7 (CH), 110.1 (C), 56.7 (CH), 28.5 ppm (CH_2_). **HRMS (FTMS ‐p NSI)** C_19_H_19_N_2_O_2_ [*M*−H]^−^ calculated for 307.1441, found 307.1437.

**(*S*)‐2‐amino‐3‐(7‐(*E*)‐styryl‐1*H*‐indol‐3‐yl)propanoic acid (30)**: The above procedure afforded 9.8 mg (65 % from 7‐Br‐tryptophan) of the desired product as a white solid.

^**1**^ **H NMR (500 MHz, MeOD)** *δ*=7.69–7.66 (m overlapped, 3 H), 7.65 (overlapped, 1 H), 7.49 (d, *J*=7.4 Hz, 1 H), 7.39 (t, *J*=7.7 Hz, 2 H), 7.30 (overlapped, 1 H), 7.29 (overlapped, 1 H), 7.27 (overlapped, 1 H), 7.12 (t, *J*=7.4 Hz, 1 H), 3.90 (dd, *J*=9.3, 4.1 Hz, 1 H, CH), 3.55 (dd, *J*=15.2, 4.1 Hz, 1 H, C*H*_A_H_B_), 3.20 ppm (dd, *J*=15.2, 9.3 Hz, 1 H, CH_A_ *H*_B_).^**13**^ **C NMR (126 MHz, MeOD)** *δ*=173.0 (CO), 137.9 (C), 135.0 (C), 128.5 (C), 128.3 (2CH), 127.9 (C), 127.0 (CH), 126.1 (2CH), 124.1 (CH), 123.9 (CH), 121.5 (C), 119.3 (CH), 118.3 (CH), 117.6 (CH), 108.7 (C), 55.3 (CH), 27.0 ppm (CH_2_). **HRMS (FTMS ‐p NSI)** C_19_H_17_N_2_O_2_ [*M*−H]^−^ calculated for 305.1296, found 305.1292.

**(*S*)‐2‐amino‐3‐(5‐(4‐methylstyryl)‐1*H*‐indol‐3‐yl)propanoic acid (31)**: The above procedure afforded 9.9 mg (62 % from 5‐Br‐tryptophan) of the desired product as a yellowish solid. LC‐MS analysis indicated the presence of separable *E* and *Z* isomers (7:1).

^**1**^ **H NMR (500 MHz, MeOD)** *δ*=7.91 (s, 1 H), 7.45 (d, *J*=8.2 Hz, 2 H), 7.43 (dd, *J*=8.6, 1.4 Hz, 1 H), 7.37 (d, *J*=8.6 Hz, 1 H), 7.26 (d, *J*=16.3 Hz, 1 H), 7.21 (s, 1 H), 7.17 (d, *J*=8.2 Hz, 2 H), 7.12 (d, *J*=16.3 Hz, 1 H), 3.91 (dd, *J*=9.7, 3.8 Hz, 1 H), 3.57 (dd, *J*=15.2, 3.8 Hz, 1 H), 3.18 (dd, *J*=15.2, 9.7 Hz, 1 H), 2.35 ppm (s, 3 H). ^**13**^ **C NMR (126 MHz, MeOD)** *δ*=173.1 (CO), 136.8 (C), 136.3 (C), 135.4 (C), 129.1 (C), 128.9 (CH), 128.9 (2CH), 127.4 (C), 125.7 (2CH), 125.2 (CH), 124.4 (CH), 120.2 (CH), 116.6 (CH), 111.3 (CH), 108.7 (C), 55.3 (CH), 27.2 (CH_2_), 19.9 ppm (CH_3_). **HRMS (FTMS +p ESI)** *m*/*z* C_20_H_21_N_2_O_2_^*+*^ [*M*+H]^+^ calculated 321.1598, found 321.1595.

**(*S*)‐2‐amino‐3‐(5‐(4‐aminostyryl)‐1*H*‐indol‐3‐yl)propanoic acid (33)**: The above procedure afforded 10.4 mg (65 % from 5‐Br‐tryptophan**)** of the desired product as a yellowish solid. LC‐MS analysis indicated the presence of separable *E* and *Z* isomers (2:1).

^**1**^ **H NMR (500 MHz, MeOD)** *δ*=7.61 (s, 1 H, ArH), 7.34 (d, *J*=8.5 Hz, 1 H, ArH), 7.21–7.18 (m, 2 H, ArH, CH=), 7.04 (d, *J*=8.4 Hz, 2 H, ArH), 6.74 (d, *J*=8.4 Hz, 1 H, ArH), 6.58 (d, *J*=12.1 Hz, 1 H,=CH), 6.55 (d, *J*=8.4 Hz, 2 H, ArH), 3.78 (dd, *J*=9.6, 4.1 Hz, 1 H, CH), 3.46 (dd, *J*=15.1, 4.1 Hz, 1 H, C*H*_A_H_B_), 3.07 ppm (dd, *J*=15.1, 9.6 Hz, 1 H, CH_A_ *H*_B_). ^**13**^ **C NMR (126 MHz, MeOD)** *δ*=172.9 (CO), 146.6 (C‐NH_2_), 136.1 (C), 129.6 (CH), 129.0 (C), 128.0 (CH), 127.4 (C), 127.0 (C), 126.7 (CH), 125.7 (CH), 124.1 (CH), 122.7 (CH), 118.5 (CH), 115.3 (CH), 114.7 (CH), 110.6 (CH), 108.5 (C), 55.3 (CH), 27.0 ppm (CH_2_). **HRMS (FTMS +p ESI)** *m*/*z* C_19_H_20_N_3_O_2_ [*M*+H]^+^ calculated 322.1550, found 322.1544.

**(*S*)‐2‐amino‐3‐(7‐(4‐aminostyryl)‐1*H*‐indol‐3‐yl)propanoic acid (34)**: The above procedure afforded 8.8 mg (55 % from 7‐Br‐tryptophan) of the desired product as a yellowish solid. LC‐MS analysis indicated the presence of separable *E* and *Z* isomers (8:1).

^**1**^ **H NMR (500 MHz, MeOD**) *δ*=7.60 (t, *J*=7.7 Hz, 1 H, ArH), 7.40 (dd, *J*=8.4, 2.9 Hz, 2 H, ArH), 7.33‐ −7.28 (m, 1 H, CH=), 7.18–7.01 (m, 2 H, ArH), 7.01–6.90 (m, 1 H, CH=), 6.73 (d, *J*=8.4 Hz, 1 H, ArH), 6.59 (s, 1 H, ArH), 6.46 (d, *J*=8.5 Hz, 1 H, ArH), 3.87 (dt, *J*=9.3, 3.4 Hz, 1 H, CH), 3.52 (dt, *J*=14.9, 4.0 Hz, 1 H, C*H*_A_H_B_), 3.15 ppm (dt, *J*=15.4, 9.1 Hz, 1 H, CH_A_ *H*_B_). ^**13**^ **C NMR (126 MHz, MeOD**) *δ*=174.4 (CO), 148.7 (C‐NH_2_), 148.5 (C), 136.2 (C), 132.5 (CH), 131.0 (CH), 129.3 (C), 128.8 (C), 128.6 (CH), 125.3 (CH), 123.7 (C), 122.7 (CH), 120.7 (CH), 120.1 (CH), 119.0 (CH), 118.1 (CH), 116.5 (CH), 109.9 (C), 56.7 (CH), 28.5 ppm (CH_2_). **HRMS (FTMS +p ESI)** *m*/*z* C_19_H_20_N_3_O_2_ [*M*+H]^+^ calculated 322.1550, found 322.1544.

**(*S*)‐2‐amino‐3‐(5‐(4‐fluorostyryl)‐1*H*‐indol‐3‐yl)propanoic acid (35)**: The above procedure afforded 14.6 mg (90 % from 5‐Br‐tryptophan) of the desired product as a yellowish solid. LC‐MS analysis indicated the presence of separable *E* and *Z* isomers (15:1).

^**1**^ **H NMR (500 MHz, MeOD)** *δ*=7.88 (s, 1 H), 7.56 (dd, *J*=8.7, 5.4 Hz, 2 H), 7.43 (dd, *J*=8.5, 1.2 Hz, 1 H), 7.36 (d, *J*=8.5 Hz, 1 H), 7.28–7.13 (m, 3 H), 7.07 (t, *J*=8.8 Hz, 2 H), 3.96 (dd, *J*=9.2, 4.0 Hz, 1 H, CH), 3.55 (dd, *J*=15.1, 4.0 Hz, 1 H, C*H*_A_H_B_), 3.20 ppm (dd, *J*=15.1, 9.2 Hz, 1 H, CH_A_ *H*_B_). ^**13**^ **C NMR (126 MHz, [D_6_]DMSO)** *δ*=170.5 (CO), 161.2 (d, *J*_C‐F_=248 Hz, CF), 136.3 (C), 134.4 (C), 130.3 (CH), 127.7 (d, *J*_C‐F_=6.2 Hz, CH),), 125.2 (CH), 123.7 (CH), 119.8 (CH), 117.5 (CH), 115.5 (d, *J*_C‐F_=21.2 Hz, CH), 111.8 (CH), 109.4 (C), 54.2 (CH), 26.9 ppm (CH_2_). ^**19**^ **F NMR (471 MHz, MeOD)** *δ*=−115.4 (major), −114.8 ppm (minor) isomer. **HRMS (FTMS +p ESI)** *m*/*z* C_19_H_18_FN_2_O_2_ [*M*+H]^+^ calculated 325.1347, found 325.1337.

**(*S*)‐2‐amino‐3‐(7‐((*E*)‐4‐fluorostyryl)‐1*H*‐indol‐3‐yl)propanoic acid (36)**: The above procedure afforded 12.9 mg (80 % from 7‐Br‐tryptophan) of the desired product as a yellowish solid.

^**1**^ **H NMR (500 MHz, MeOD)** *δ*=7.68–7.65 (m overlapped, 3 H), 7.58 (d, *J*=16.3 Hz, 1 H), 7.47 (d, *J*=7.4 Hz, 1 H), 7.29 (s, 1 H), 7.27 (d, *J*=16.3 Hz, 1 H), 7.14–7.10 (m, overlapped, 3 H), 3.89 (dd, *J*=9.3, 4.1 Hz, 1 H, CH), 3.55 (dd, *J*=15.3, 4.1 Hz, 1 H, C*H*_A_H_B_), 3.20 ppm (dd, *J*=15.3, 9.3 Hz, 1 H, CH_A_ *H*_B_). ^**13**^ **C NMR (126 MHz, MeOD)** *δ*=173.0 (CO), 162.2 (d, *J*_C‐F_=245.8 Hz, CF), 135.0 (C), 134.3 (C), 127.9 (C), 127.8 (d, *J*_C‐F_=7.9 Hz, CH), 127.2 (CH), 124.1 (CH), 123.9 (CH), 121.4 (C), 119.3 (CH), 118.3 (CH), 117.7 (CH), 115.1 (d, *J*_C‐F_=21.9 Hz, CH), 108.7 (C), 55.4 (CH), 27.0 ppm (CH_2_). ^**19**^ **F NMR (471 MHz, MeOD)** *δ*=−117 ppm. **HRMS (FTMS +p ESI)** *m*/*z* C_19_H_18_FN_2_O_2_ [*M*+H]^+^ calculated 325.1347, found 325.1342.

**(*S*)‐2‐amino‐3‐(7‐((*E*)‐3‐fluorostyryl)‐1*H*‐indol‐3‐yl)propanoic acid (37)**: The above procedure afforded 14.9 mg (92 % from 7‐Br‐tryptophan) of the desired product as a white solid.

^**1**^ **H NMR (500 MHz, MeOD)** *δ*=7.73–7.62 (m, 2 H), 7.49 (d, *J*=7.4 Hz, 1 H), 7.45–7.33 (m, 3 H), 7.26 (d, *J*=17.0 Hz, 2 H), 7.11 (t, *J*=7.6 Hz, 1 H), 6.98 (td, *J*=8.3, 1.9 Hz, 1 H), 3.91 (dd, *J*=9.1, 4.1 Hz, 1 H, CH), 3.53 (dd, *J*=15.2, 4.1 Hz, 1 H, C*H*_A_H_B_), 3.20 ppm (dd, *J*=15.2, 9.1 Hz, 1 H, CH_A_ *H*_B_). ^**13**^ **C NMR (126 MHz, MeOD)** *δ*=174.2 (CO), 164.7 (d, *J*_C‐F_=242 Hz, CF), 141.9 (d, *J*_C‐F_=8.8 Hz, C), 136.5 (C), 131.3 (d, *J*_C‐F_=8.8 Hz, CH), 129.4 (C), 128.3 (CH), 126.9 (CH), 125.6 (CH), 123.7 (CH), 122.4 (C), 120.7 (CH), 119.9 (CH), 119.4 (CH), 114.9 (d, *J*_C‐F_=22 Hz, H‐C‐CF), 113.4 (d, *J*_C‐F_=28 Hz, H‐C‐CF), 110.00 (C), 56.5 (CH), 28.3 ppm (CH_2_). ^**19**^ **F NMR (377 MHz, [D_6_]DMSO)** *δ*=−113.5 ppm. **HRMS (FTMS +p ESI)** *m*/*z* C_19_H_18_FN_2_O_2_ [*M*+H]^+^ calculated 325.1347, found 325.1338.

**(*S*)‐2‐amino‐3‐(5‐((*E*)‐4‐cyanostyryl)‐1*H*‐indol‐3‐yl)propanoic acid (38)**: The above procedure afforded 15 mg (91 % from 5‐Br‐tryptophan) of the desired product as a yellowish solid.

^**1**^ **H NMR (500 MHz, MeOD)** *δ*=7.96 (s, 1 H, ArH), 7.77–7.61 (m, 4 H, ArH, CH=), 7.60–7.44 (m, 2 H, ArH), 7.39 (d, *J*=8.5 Hz, 1 H, ArH), 7.26–7.13 (m, 2 H, ArH), 3.93 (dd, *J*=9.1, 4.1 Hz, 1 H, CH), 3.54 (dd, *J*=15.1, 3.9 Hz, 1 H, C*H*_A_H_B_), 3.21 ppm (dd, *J*=15.2, 9.1 Hz, 1 H, CH_A_ *H*_B_). ^**13**^ **C NMR (126 MHz, DMSO)** *δ*=169.8 (CO), 142.8 (C), 136.7 (C), 134.4 (CH), 132.6 (CH), 127.6 (C), 127.1 (C), 126.6 (CH), 125.1 (CH), 123.1 (CH), 120.1 (CH), 119.3 (C), 118.5 (CH), 111.8 (CH), 110.4 (C), 108.5 (C), 54.7 (CH), 27.1 ppm (CH_2_). **HRMS (FTMS ‐p NSI)** C_20_H_16_N_3_O_2_ [*M*−H]^−^ calculated for 330.1248, found 330.1245.

**(*S*)‐2‐amino‐3‐(7‐((*E*)‐4‐cyanostyryl)‐1*H*‐indol‐3‐yl)propanoic acid (39)**: The above procedure afforded 14.7 mg (89 % from 7‐Br‐tryptophan) of the desired product as a yellowish solid.

^**1**^ **H NMR (500 MHz, MeOD)** *δ*=7.86–7.75 (m, 3 H, ArH), 7.74–7.65 (m, 3 H, ArH, CH=), 7.51 (d, *J*=7.4 Hz, 1 H, ArH), 7.31 (d, *J*=16.1 Hz, 1 H, CH=), 7.29 (s, 1 H, ArH), 7.13 (t, *J*=7.7 Hz, 1 H, ArH), 3.98 (dd, *J*=8.8, 4.4 Hz, 1 H, CH), 3.52 (dd, *J*=15.3, 4.1 Hz, 1 H, C*H*_A_H_B_), 3.23 ppm (dd, *J*=15.3, 8.8 Hz, 1 H, CH_A_ *H*_B_). ^**13**^ **C NMR (126 MHz, MeOD)** *δ*=173.6 (CO), 144.2 (C), 136.5 (C), 133.6 (CH), 132.9 (CH), 130.7 (CH), 129.4 (CH), 128.1 (CH), 127.8 (CH), 125.9 (CH), 122.1 (C), 120.8 (CH), 120.4 (CN), 112.0 (CH), 111.2 (C), 109.8 (C), 55.7 ppm (CH), 28.0 (CH_2_). **HRMS (FTMS ‐p NSI)** C_20_H_16_N_3_O_2_ [*M*−H]^−^ calculated for 330.1248, found 330.1245.

**(*S*)‐2‐amino‐3‐(5‐(2‐(pyridin‐4‐yl)vinyl)‐1*H*‐indol‐3‐yl)propanoic acid (40)**: The above procedure afforded 13 mg (85 % from 5‐Br‐tryptophan) of the desired product as a reddish solid. LC‐MS analysis indicated the presence of separable *E* and *Z* isomers (3:1).

^**1**^ **H NMR (500 MHz, MeOD)** *δ*=8.51 (d, *J*=5.9 Hz, 2 H, ArH), 7.86 (s, 1 H, ArH), 7.66 (d, *J*=16.4 Hz, 1 H, CH=CH‐Py), 7.56 (d, *J*=5.5 Hz, 2 H, ArH), 7.47 (d, *J*=8.5 Hz, 1 H, ArH), 7.38 (d, *J*=8.4 Hz, 1 H, ArH), 7.23 (s, 1 H, ArH), 7.13 (d, *J*=16.4 Hz, 1 H, CH=C*H*‐Py), 3.91 (dd, *J*=9.2, 3.6 Hz, 1 H, CH), 3.56 (dd, *J*=15.0, 3.6 Hz, 1 H, C*H*_A_H_B_), 3.21 ppm (dd, *J*=15.0, 9.2 Hz, 1 H, CH_A_ *H*_B_). ^**13**^ **C NMR (126 MHz, MeOD)** *δ*=173.0 (CO), 146.9 (CH‐N), 131.1 (C), 129.6 (CH), 127.9 (C), 127.2 (CH), 126.8 (C), 123.8 (CH), 122.1(C), 121.3 (CH), 119.3 (CH), 118.7 (CH), 116.9 (CH), 108.5 (C), 55.3 (CH), 27.2 ppm (CH_2_). **HRMS (FTMS +p ESI)** *m*/*z* C_18_H_18_N_3_O_2_ [*M*+H]^+^ calculated 308.1394, found 308.1388.

**(*S*)‐2‐amino‐3‐(7‐((*E*)‐2‐(pyridin‐4‐yl)vinyl)‐1*H*‐indol‐3‐yl)propanoic acid (41)**: The above procedure afforded 10.9 mg (71 % from 7‐Br‐tryptophan) of the desired product as a reddish solid.

^**1**^ **H NMR (500 MHz, MeOD)** *δ*=8.47 (d, *J*=5.8 Hz, 2 H, ArH), 7.95 (d, *J*=16.4 Hz, 1 H, CH=CH‐Py), 7.74 (d, *J*=7.6 Hz, 1 H, ArH), 7.64 (d, *J*=6.1 Hz, 2 H, ArH), 7.54 (d, *J*=7.4 Hz, 1 H, ArH), 7.30 (s, 1 H, ArH), 7.26 (d, *J*=16.3 Hz, 1 H, CH=C*H*‐Py), 7.14 (t, *J*=7.7 Hz, 1 H, ArH), 3.88 (dd, *J*=9.1, 4.2 Hz, 1 H, CH), 3.52 (dd, *J*=15.3, 4.2 Hz, 1 H, C*H*_A_H_B_), 3.20 ppm (dd, *J*=15.3, 9.1 Hz, 1 H, CH_A_ *H*_B_). ^**13**^ **C NMR (126 MHz, MeOD)** *δ*=174.3 (CO), 150.2 (2CH‐N), 148.1 (C), 136.6 (C), 131.3 (CH=CH‐Py), 129.6 (C), 126.3 (CH=C*H*‐Py), 125.8 (CH), 122.4 (2CH), 121.6 (C), 120.8 (CH), 120.7 (CH), 120.6 (CH), 110.3 (C), 56.6 (CH), 28.3 ppm (CH_2_). **HRMS (FTMS +p ESI)** *m*/*z* C_18_H_18_N_3_O_2_ [*M*+H]^+^ calculated 308.1394, found 308.1392.

### Barettin purification from extract^21^

Barettin extract was kindly provided by Dr. Paco Cárdenas, Uppsala University, Sweden; 2 g of freeze‐dried extract was added onto a filter paper in a funnel and rinsed copiously with dichloromethane to remove any lipids from the sample. After rinsing with DCM, the freeze‐dried extract was washed thoroughly with 60 % aqueous acetonitrile. The washings were checked via LCMS and combined. The sample was concentrated to 1.5 mL.

Barretin extract was purified via RP‐HPLC using a Phenomenex Luna C18 (5 micron, 250×21.2 mm) with UV detection at 234 nm. The compound was eluted using a shallow linear gradient with starting conditions 95 % solvent A (0.1 % formic acid in MQ water) to 5 % solvent B (ACN) to 40 % solvent B over 40 min. Over the next 15 min solvent B was increased to 95 %, held isocratically for 5 min before returning to starting conditions. Barettin (8 mg) eluted with a retention time of 37 min and was confirmed by LCMS and characterised by NMR.

**(*R*)‐1‐(3‐(5‐(((*Z*)‐6‐bromo‐1*H*‐indol‐3‐yl)methylene)‐3,6‐dioxopiperazin‐2‐yl)propyl)guanidine (42)**: ^**1**^ **H NMR (700 MHz, MeOD)** *δ*=7.75 (s, 1 H, Ar‐CH), 7.66–7.46 (m, 2 H), 7.24 (dd, 1 H, *J*=8.5, 1.7 Hz, Ar‐CH), 7.17 (s, 1 H, Ar‐CH), 4.20 (t, 1 H, *J*=5.6 Hz, CH), 3.23–3.19 (m, 2 H, CH_2_), 1.99–1.93 (m, 1 H, C*H*_A_H_B_) 1.92–1. 85 (m, 1 H, CH_A_ *H*_B_), 1.77–1.66 ppm (m, 2 H, CH_2_). ^**13**^ **C NMR (176 MHz, MeOD)** *δ*=167.2 (CO), 162.1 (CO), 157.2 (C(NH)_2_NH_2_), 137.0 (C), 126.2 (CH), 126.1 (C), 123.2 (CH), 121.9 (CH), 119.5 (CH), 115.7 (C), 114.3 (CBr), 109.7 (CH), 108.5 (C), 55.1 (CH), 40.5 (CH_2_), 31.2 (CH_2_), 23.7 ppm (CH_2_). **MS (ESI)** *m*/*z* 421 (100) [*M*(^81^Br)+H]^+^, 419 (100) [*M*(^79^Br)+H]^+^; **HRMS (FTMS +p ESI)**: *m*/*z* calculated for: C_17_H_20_BrN_6_O [*M*(^79^Br)+H]^+^: 419.0826; found: 419.0809.

**Heck cross‐coupling on purified Barettin in aqueous conditions giving 1‐(3‐((*R*)‐5‐(*Z*)‐((6‐(4‐fluorostyryl)‐1*H*‐indol‐3‐yl)methylene)‐3,6‐dioxopiperazin‐2‐yl)propyl)guanidine (43)**: A stock solution of catalyst was prepared as follows: sodium tetrachloropalladate (1.5 mg, 5 μmol), TXPTS (7.0 mg, 11.5 μmol, 23 mol %) were purged with argon and stirred at RT for 15 min after adding 1 mL of degassed water/acetonitrile (1:1) mixture. Then, in a separate MW vial barretin (2 mg, 0.005 mmol) was added together with Na_2_CO_3_ (2 mg, 0.02 mmol, 4 equiv) followed by addition of the 4‐fluorostyrene (4 μL, 0.15 mmol, 10 equiv) and Pd‐catalyst (10 mol % from stock). The vial was closed, and the reaction mixture was stirred and heated at 90 °C (MW) for 2.5 hours. LC‐MS analysis showed full conversion. Purification was done via RP‐HPLC using a Phenomenex Luna C18 (5 micron, 250×21.20 mm) with UV detection at 330 nm. The compound was eluted using a shallow linear gradient with starting conditions 95 % solvent A (0.1 % formic acid in MQ water) to 5 % solvent B (ACN) to 40 % solvent B over 40 min. Over the next 15 min solvent B was increased to 95 %, held isocratically for 5 min before returning to starting conditions. Barettin‐Heck derivative eluted with a retention time of 39 min and was confirmed by LCMS and ^1^H NMR. Depicted product was obtained 0.5 mg (21 % isolated yield) as a white solid.

^**1**^ **H NMR (700 MHz, MeOD)** *δ*=7.81 (s, 1 H, Ar‐CH), 7.69 (d, 1 H, *J*=8.4 Hz, Ar‐CH) 7.65–7.54 (m, 3 H), 7.47 (d, 1 H, *J*=8.4 Hz, Ar‐CH) 7.27 (s, 1 H, Ar‐CH), 7.19 (d, 1 H, *J*=16.3 Hz, Ar‐CH), 7.11 (t, 2 H, *J*=8.7 Hz, 2 H) 4.25 (t, 1 H, *J*=5.6 Hz, CH), 3.27–3.22 (m, 2 H, CH_2_), 2.03–1.98 (m, 1 H, C*H*_A_H_B_) 1.96–1. 89 (m, 1 H, CH_A_ *H*_B_), 1.82–1.70 ppm (m, 2 H, CH_2_). ^**19**^ **F NMR (659 MHz, MeOD)** *δ*=−77.1 ppm. **MS (ESI)** *m*/*z* 461.2 (100); **HRMS (FTMS +p ESI)**: *m*/*z* calculated for C_25_H_26_FN_6_O_2_ [*M*+H]^+^:461.2096; found: 461.2075.

### Culture conditions and isolation of pacidamycin D and Br‐pacidamycin D (44).^8^

Starter cultures of engineered strain *Streptomyces coelicolor* RG1104, with *prnA* knock‐in (performed as previously reported)^3^ were obtained by inoculating 150 mL ISP2 medium with 0.3 mL spore suspension (approximately 10^6^–10^7^ cfu mL^−1^ final concentration) and culturing for 24–48 hours at 28 °C, 220 rpm. Starter culture (20 mL) was then added to 0.5 L ISP2 and incubated with shaking at 28 °C for 7–8 days. Pacidamycins were extracted from the cell‐free broth using 0.05 volumes of XAD‐16 resin. The resin was washed with 20 volumes of water and the extract was eluted with 10 volumes of methanol. The solvent was removed in vacuo. The crude extract was then purified by ion‐exchange chromatography using a 5 mL HiTrap SP‐FF column (GE Healthcare). After loading, the column was washed with 6 volumes 50 mm sodium acetate, pH 3.6. Pacidamycins were eluted with 50 mm sodium acetate in a stepwise gradient from pH 3.6 to pH 5.6. the appropriate pacidamycin‐containing fractions were combined and further purified on a Luna C18(2) 250×22.10 mm column, initial composition 10 % acetonitrile 90 % RP buffer A, held for 2 min before reaching 40 % acetonitrile over 40 min compound eluted at around 28 min. Over the next 20 min quick gradient up to 95 % acetonitrile which was held for 5 min before returning to starting conditions.

**Pacidamycin‐D**: ^**1**^ **H NMR (700 MHz, MeOD)** *δ*=7.61 (d, *J*=7.7 Hz, 1 H), 7.45 (d, *J*=8.2 Hz, 1 H), 7.31 (dd, *J*=8.1, 1.8 Hz, 1 H), 7.20 (s, 2 H), 7.12–7.09 (m, 1 H), 5.98 (s, 1 H), 5.88 (s, 1 H), 5.76 (dd, *J*=8.1, 1.9 Hz, 1 H), 4.52 (ddq, *J*=8.6, 6.5, 2.1 Hz, 3 H), 4.45 (dd, *J*=9.3, 2.0 Hz, 1 H), 4.25 (qd, *J*=7.0, 1.9 Hz, 1 H), 3.28–3.16 (m, 2 H), 2.79 (d, *J*=1.9 Hz, 3 H), 2.59 (d, *J*=17.8 Hz, 1 H), 1.41 (dd, *J*=7.1, 1.9 Hz, 1 H), 1.29 (dd, *J*=7.0, 1.9 Hz, 3 H), 1.22 (d, *J*=7.2 Hz, 3 H), 1.12 ppm (dd, *J*=6.9, 1.9 Hz, 3 H).

**MS (ESI)** *m*/*z* 712 (100) **HRMS (FTMS +p ESI)**: *m*/*z* calculated for C_32_H_42_N_9_O_10_ [*M*+H]^+^ 712.3049; found: 712.3044.

**Br‐Pacidamycin‐D (44)**: Tabulated HSQC NMR data and comparison with wild type pacidamycin D is given in SI. **MS (ESI)**: 790 (100) [*M*(^79^Br)+H]^+^, 792 (100) [*M*(^81^Br)+H]^+^; **HRMS (FTMS +p ESI)**: *m*/*z* calculated for C_32_H_41_BrN_9_O_10_^+^ [*M*(^79^Br+H)]^+^: 790.2154; found: 790.2150.

### Heck cross‐coupling of Br‐pacidamycin D to give 45

To a solution of the purified 7‐Br‐pacidamycin D (0.5 mg) in degassed water‐acetonitrile (3:1), sodium tetrachloropalladate (10 mol %), TXPTS (23 mol %) (from stock solution in degassed water‐acetonitrile (3:1) 10 mm) were added followed by Na_2_CO_3_ (4 equiv) and 4‐fluorostyrene (5 equiv) were added. The vial was closed and the reaction mixture was stirred and heated at 90 °C (MW) for 2 hour. After completion, the reaction was cooled down to RT was diluted with water (10 mL) and acidified (pH=2–3) using 0.1 m HCl. The resulting mixture was extracted with ethyl acetate (3×10 mL). Complete conversion was observed by LC‐HRMS analysis of the crude reaction mixture. Attempted purification by HPLC was not successful to isolate desired product, which may be due to very low quantities of product **45**.

**MS (ESI)**: 832 (100) [*M*+H]^+^; **HRMS (FTMS +p ESI)**: *m*/*z* calculated for C_40_H_47_FN_9_O_10_^+^ [*M*+H]^+^: 832.3424; found: 832.3422.

## Conflict of interest

The authors declare no conflict of interest.

## Supporting information

As a service to our authors and readers, this journal provides supporting information supplied by the authors. Such materials are peer reviewed and may be re‐organized for online delivery, but are not copy‐edited or typeset. Technical support issues arising from supporting information (other than missing files) should be addressed to the authors.

Supplementary
