# Supplementary material for: Heck Diversification of Indole‐Based Substrates under Aqueous Conditions: From Indoles to Unprotected Halo‐tryptophans and Halo‐tryptophans in Natural Product Derivatives
Source: Chemistry. 2019 Jul 19;25(46):10866–75. doi: 10.1002/chem.201901327 (PMC6772188; doi:10.1002/chem.201901327)

CHEMISTRY
A European Journal

Supporting Information

Heck Diversification of Indole-Based Substrates under Aqueous Conditions: From Indoles to Unprotected Halo-tryptophans and Halo-tryptophans in Natural Product Derivatives

Cristina Pubill-Ulldemolins+,[a, b] Sunil V. Sharma+,[a] Christopher Cartmell+,[a] Jinlian Zhao,[a] Paco Cárdenas,[c] and Rebecca J. M. Goss*[a]

chem_201901327_sm_miscellaneous_information.pdf

Supporting Information

Index

	page
General experimental procedures	3
General procedure for LC-HRMS^2 analyses	4
Table S-1: Screening of conditions for Heck cross-coupling of halo-indoles with acrylic acid in aqueous media	5
General protocol for Heck Cross-coupling of halo-indoles with alkenes in aqueous conditions; and Characterisation data for indole-Heck products	6
N-Boc protection of 4-Br-(*S*)-tryptophan	9
Heck Cross-coupling of *N*-Boc-4-Br-(*S*)-tryptophan with acrylic acid in aqueous conditions	10
One pot Heck Cross-coupling and *N*-Boc deprotection of *N*-Boc-4-Br-tryptophan with acrylic acid in aqueous conditions	11
General protocol for Heck Cross-coupling of *N*-Boc protected halo-aminoacids with acrylic acid in aqueous media	11
Table S-2: Comparison of ^{1}H and ^{13}C peaks extracted from HSQC NMR spectra for Pacidamycin D and Br-pacidamycin D	14
Heck cross-coupling of Br-pacidamycin D	17
NMR spectra of purified products.	21

General experimental procedures

All reagents were purchased from commercial suppliers and were used without further purification unless otherwise stated. Proton NMR (^{1}H), and carbon NMR (^{13}C) were recorded on either a Bruker Ascend HD700 (700 MHz), Bruker Ascend 500 (500 MHz) or a Bruker 400 UltraShield (400 MHz) spectrometer. The NMR experiments were carried out in deuterated chloroform ($CDCl_3$) deuterated water (D_2O), deuterated DMSO (DMSO-d_6) or deuterated methanol (d_4-MeOH). The chemical shifts (δ) are quoted in parts per million (ppm). Using a DEPTQ sequence or an HSQC experiment with multiplicity editing, the ^{13}C NMR signals were identified to CH_3, CH_2, CH and C. Coupling constants are reported in Hertz (Hz).

High and low resolution mass spectra that were recorded at the University of St Andrews on an Orbitrap VELOS pro. Freeze drying was carried out on a Scanvac CoolSafe™ freeze dryer. Microwave reactions were effected in sealed vials using a Biotage Initiator$^+$ microwave reactor. UPLC analysis was acquired on a Waters Acquity H-Class UPLC system fitted with a Waters Acquity UPLC BEH C18 column (1.7 μm, 2.1×50 mm) or Phenomenex Kinetex Phenyl-hexyl column (2.1 μm, 2.1×75 mm).

Flash chromatography was performed using Davisil silica gel LC60A (40-63 micron). Thin layer chromatography (TLC) was executed using aluminium sheets of silica gel 60 F254 and was visualised under a Mineralight model UVGL-58 lamp (254 nm). The plates were developed with ninhydrin in acetone or basic potassium permanganate solutions. Purification of unprotected tryptophan derivatives and peptides was carried out on a Biotage Isolera Four using reverse-phase SNAP C18 12 g column cartridges. The purification was carried out using water (solvent A) and methanol/acetonitrile (solvent B) using the following gradient: 0-1.5 min (5% B), 1.5-3.0 min (5% to 15% B), 3.0-5.0 min (15% B), 5.0-15.0 min (15% to 95% B), 15.0-18.0 min (95% B), 18.0-20.0 min (95%-5% B), 20.0-25.0 min (5% B) at a flow rate of 12-15 mL/min.

Preparative RP-HPLC purification was performed using a Gilson 322 pump, 151 UV/VIS detector and 233XL fraction collector, using a Phenomenex Luna C18 (5 micron, 250 ×21.2 mm) with UV detection at 234 nm. Elution was carried out using a shallow linear gradient with starting conditions 95% solvent A (0.1% formic acid in MQ water) to 5% solvent B (ACN) to 40% solvent B over 40 mins.

General procedure for LC-HRMS^2 analyses:

Samples were analysed by LC-HRMS^2 using a Thermo Orbitrap Velos Pro system described in the chemical experimental procedures equipped with a Phenomenex C-18 Kinetix exo (3.5 μm, 2.1×100 mm column) at 40 °C. An injection volume of 10 μL was used for all samples. Analytes were eluted using an initial solvent composition of 95% water/0.1% formic acid (solvent A) and 5% acetonitrile (solvent B) at a flow rate of 0.35 mL/min that was held for 1 minute followed by a linear gradient to 95% solvent B over 10 minutes. This solvent composition was held for a further 2 minutes before returning to initial conditions over 0.5 minutes. Eluent from the column was passed through a PDA monitoring absorbance from 220-800 nm (2 nm resolution, 10 Hz) and a valve which diverted eluent from the first minute of each run to waste. After the first minute, the valve switched to pass eluent through to the inlet valve of the H-ESI source, which was set to positive ionisation mode using a 300 °C heater temperature, 350 °C capillary temperature, 50 U sheath gas flow, 20 U aux gas flow, 2 U sweep gas flow, 3.5 kV ionisation voltage and 50% RF lens power. The scan cycle consisted of one high-resolution survey scan and three data-dependent fragmentation scans. The survey scan was analysed in the orbitrap FTMS analyser at a resolution of 30,000 (at 400 *m/z*) over a range of 1500 – 1500 *m/z*, using a background ion corresponding to the $[M + H]^+$ charge state of n-butyl benzenesulfonamide (exact mass 214.08963) as a lock-mass for internal scan-by- scan calibration.

Table S-1: Screening of conditions for Heck cross-coupling of halo-indoles with acrylic acid in aqueous media

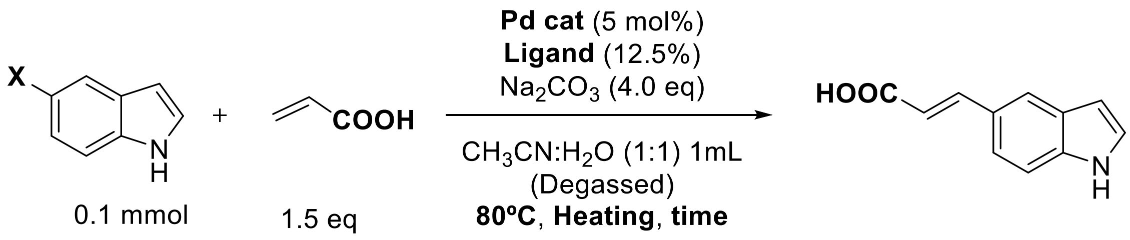

Entry	X	Pd-catalyst	Ligand	Heating	time	Conv. (%) (Isol. Yield)
1	**5-I**	$Pd(OAc)_2$	TXPTS	Reflux	18h	78
2	5-I	"	sSPhos	"	"	97(86)
3	5-I	Na_2PdCl_4	"	MW	1h	>99(90)
5	5-I	"	none	"	"	>99(91)
6	**5-Br**	"	"	"	"	48
7	5-Br	"	TXPTS	"	"	77
8	5-Br	"	sSPhos	"	"	>99(94)
9	**5-Cl**	"	"	"	"	<1(-)

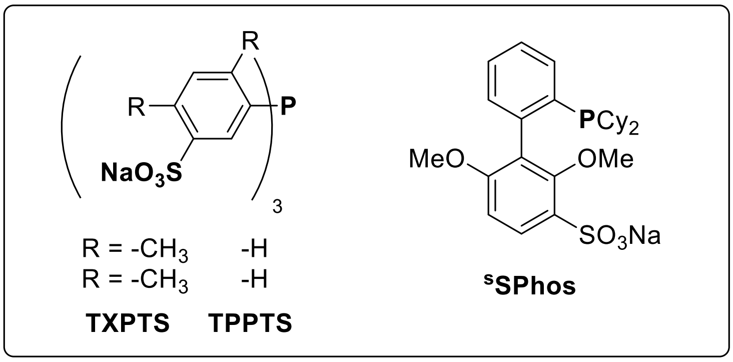

General protocol for Heck Cross-coupling of halo-indoles with alkenes in aqueous conditions.

In a 10 mL pear-shape flask or 5 mL MW vial, sodium tetrachloropalladate (1.6 mg, 5 μmol, 5 mol%), sulfonated SPhos (6.6 mg, 12.5 μmol, 12.5 mol%) were purged with nitrogen and stirred at rt for 15 min after adding 1 mL of degassed water/acetonitrile (1:1) mixture. Then, appropriate halo-indole (0.1 mmol, 1.0 eq) is added together with Na_2CO_3 (22 mg, 0.2 mmol, 2eq) followed by addition of the alkene (0.15 mmol, 1.5 eq). The reaction mixture was heated at 80 °C (MW or conventional heating) for the required period of time. The reaction mixture was cooled to r.t. and diluted with 5 mL of a saturated solution of $NaHCO_3$. The aqueous layer was extracted with ethyl acetate (3×10 mL). The combined organic layers were dried over anhydrous Na_2SO_4 and the solvent removed *in vacuo*. Purification by column chromatography using silica gel (Hexanes/ethyl acetate 4:1).

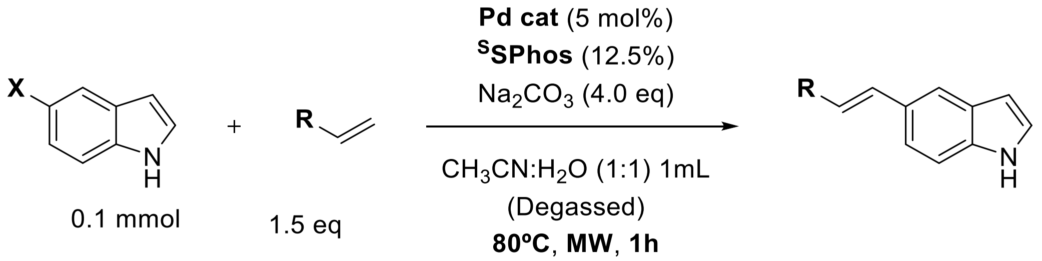

(*E*)-3-(1H-indol-5-yl)acrylic acid (3)[1]

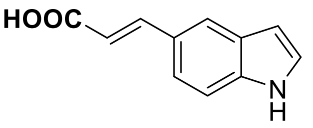

The above procedure afforded 17.6 mg (94% from 5-Br-indole) and 17.2 mg (92% from 5-I-indole) of the title product as a white solid.

^{1}H NMR (400 MHz, MeOD) δ 7.86 – 7.73 (m, 2H, ArH), 7.43 – 7.40 (m, 2H, ArH, CH=), 7.27 (d, J = 3.2 Hz, 1H, ArH), 6.50 (dd, J = 3.2, 0.6 Hz, 1H, ArH), 6.39 (d, J = 15.9 Hz, 1H, CH=).

^{13}C NMR (101 MHz, MeOD) δ 171.3 (CO), 148.9 (CH=), 139.1 (C), 129.7 (C), 126.9 (C), 126.9 (CH), 123.4 (CH), 121.8 (CH), 115.1 (CH=), 112.8 (CH), 103.4 (CH).

HRMS (FTMS -p NSI) $C_{11}H_8NO_2$ [M - H]$^-$ calculated for 186.0561, found 186.0561.

[1] Pisano, C. *et al. PCT, Appl.,* 2006, 2006131482.

Ethyl (*E*)-3-(1H-indol-5-yl)acrylate (13)[2]

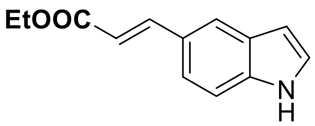

The above procedure afforded 15.3 mg (71% from 5-Br-indole) and 12.9 mg (60% from 5-I-indole) of the title product as a white solid.

^{1}H NMR (400 MHz, $CDCl_3$) δ 8.36 (bs, 1H, NH), 7.93 – 7.77 (m, 2H, ArH), 7.49 – 7.32 (m, 2H, ArH, CH=), 7.23 (dd, J = 3.2, 2.4 Hz, 1H, ArH), 6.63 – 6.55 (m, 1H), 6.42 (d, J = 15.9 Hz, 1H, CH=), 4.28 (q, J = 7.1 Hz, 2H, CH_2), 1.35 (t, J = 7.1 Hz, 3H, CH_3).

^{13}C NMR (101 MHz, $CDCl_3$) δ 167.7 (CO), 146.4 (CH=C), 136.9 (C), 128.1 (C), 126.6 (C), 125.2 (CH), 122.3 (CH), 121.6 (CH), 115.1 (CH=), 111.5 (CH), 103.5 (CH), 60.3 (CH_2), 14.4 (CH_3).

HRMS (FTMS +p NSI) $C_{13}H_{13}NO_2H$ [M + H]$^+$ calculated for 216.1019, found 216.1019.

(*E*)-3-(1H-indol-7-yl)acrylic acid (14)[3]

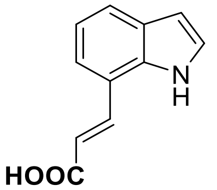

The above procedure afforded 16.6 mg (89% from 7-Br-indole) and 17 mg (91% from 7-I-indole) of the title product as a white solid.

^{1}H NMR (400 MHz, MeOD) δ 8.15 (d, J = 16.0 Hz, 1H, =CH), 7.63 (dd, J = 7.8, 1.0 Hz, 1H, ArH), 7.42 (dt, J = 7.5, 0.6 Hz, 1H, ArH), 7.29 (d, J = 3.2 Hz, 1H, ArH), 7.10 – 7.00 (m, 1H, ArH), 6.58 (d, J = 16.0 Hz, 1H, CH=C), 6.51 (d, J = 3.2 Hz, 1H, ArH).

^{13}C NMR (101 MHz, MeOD) δ 170.8 (CO), 142.9 (CH=C), 135.9 (C), 130.7 (C), 126.5 (CH), 124.1 (CH), 122.2 (CH), 120.4 (CH), 119.5 (C), 118.4 (CH=), 103.1 (CH).

HRMS (FTMS -p NSI) $C_{11}H_8NO_2$ [M - H]$^-$ calculated for 186.0561, found 186.0562.

---

[2] Verma, K. A. *et al. Org Lett.* 2015, (**17**), 3658-3661.
[3] Singh, J. *et al. J. Med. Chem.* 2010, (**53**), 18-36.

(*E*)-4-(1H-indol-5-yl)-but-3-en-2-one (15)[4]

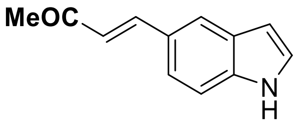

The above procedure afforded 15.7 mg (85% from 5-Br-indole) of the title product as a white solid.

^{1}H NMR (400 MHz, CDCl$_3$) δ 8.54 (bs, 1H, NH), 7.85 (dd, *J* = 1.6, 0.8 Hz, 1Hn ArH), 7.70 (d, *J* = 16.2 Hz, 1H, CH=C), 7.51 – 7.36 (m, 2H, ArH), 7.31 – 7.23 (m, 1H, ArH), 6.75 (d, *J* = 16.1 Hz, 1H, C=CH), 6.61 (ddd, *J* = 3.1, 2.0, 0.9 Hz, 1H, ArH), 2.42 (s, 3H, CH$_3$).

^{13}C NMR (101 MHz, CDCl$_3$) δ 198.8 (CO), 145.7 (CH=), 137.2 (C), 128.2 (C), 126.3(C), 125.4 (CH), 124.6 (CH), 122.8 (CH), 121.7 (CH), 111.7 (CH), 103.5 (CH), 27.3 (CH$_3$).

HRMS (FTMS +p NSI) $C_{12}H_{12}NO$ $[M + H]^+$ calculated for 186.0913, found 186.0911.

(*E*)-3-(1H-indol-5-yl)acrylonitrile (16)[5]

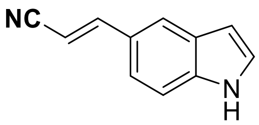

The above procedure afforded 15.3 mg (85% from 5-Br-indole) of the title product as a white solid.

^{1}H NMR (400 MHz, MeOD) δ 7.76 (s, 1H), 7.59 (dd, *J* = 16.6, 2.0 Hz, 1H, CH=C), 7.42 – 7.34 (m, 2H, overlapping ArH & C=CH), 7.28 (d, *J* = 3.2 Hz, 1H, ArH), 6.51 (dd, *J* = 3.2, 0.8 Hz, 1H, ArH), 6.02 (d, *J* = 16.6 Hz, 1H, C=CH).

^{13}C NMR (101 MHz, MeOD) δ 154.3 (CH=), 139.3 (C), 129.6 (CH), 127.2 (CH), 126.7 (C), 122.9 (CH), 121.0 (CH), 120.4 (C), 112.9 (CH), 103.5 (CH), 92.2 (=CH).

HRMS (FTMS -p NSI) $C_{11}H_8NO_2H$ $[M + H]^+$ calculated for 169.0760, found 169.0757.

[4] Boyle, J. *U.S. Pat. Appl. Publ.,* 2008, 20080267942.
[5] Burnett, D. A. *U.S. Pat. Appl. Publ.,* 2005, 20050137210 A1 20050623.

(*E*)-3-(1H-indol-6-yl)acrylic acid (18)

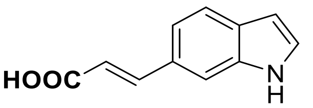

The above procedure afforded 15.5 mg (83% from 6-Br-indole) of the title product as a yellowish solid.

^{1}H NMR (400 MHz, MeOD) δ 7.79 (d, J = 15.9 Hz, 1H, CH=C), 7.62 – 7.53 (m, 2H, ArH), 7.37 – 7.28 (m, 2H, ArH), 6.47 (d, J = 4.0 Hz, 1H, ArH), 6.43 (d, J = 15.9 Hz, 1H, C=CH).

^{13}C NMR (101 MHz, MeOD) δ 171.1 (CO), 148.6 (CH=C), 137.7 (C), 133.6 (C), 131.6 (C), 128.9 (C), 128.2 (CH), 121.6 (CH), 115.8 (CH), 113.5 (CH), 102.9 (CH).

HRMS (FTMS -p NSI) $C_{11}H_8NO_2$ $[M - H]^-$ calculated for 186.0561, found 186.0562.

_N_-Boc protection of 4-Br-(*S*)-tryptophan

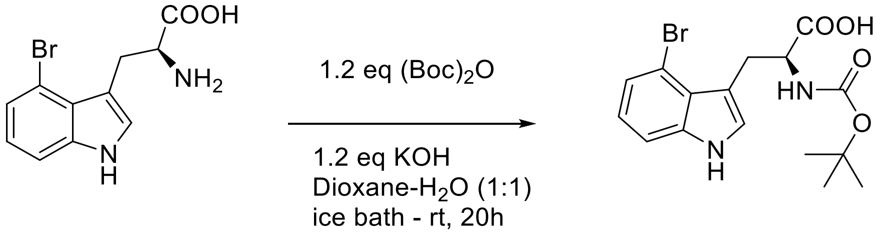

(*S*)-3-(4-bromo-1H-indol-3-yl)-2-((*tert*-butoxycarbonyl)amino)propanoic acid

A suspension of 4-bromo-(*S*)-tryptophan (150 mg, 0.53 mmol, 1.0 eq) and di-*t*-butyl dicarbonate (138 mg, 0.64 mmol, 1.2 eq) in 1,4-dioxane-water (1:1, 4 mL) was cooled to 0 °C. Aqueous KOH (1.8 N, 0.37 mL, 0.64 mmol, 1.2 eq) was added dropwise. The mixture was stirred overnight while warming to room temperature. The reaction was diluted with water (10 mL) and extracted with diethyl ether (2×10 mL). The aqueous layer was cooled in an ice-bath and the pH was adjusted to 2 using 1 M HCl. The resultant white suspension was extracted using ethyl acetate (3×10 mL). The combined organic layers were dried over anhydrous Na_2SO_4 and the solvent was removed *in vacuo* to give (*S*)-3-(4-bromo-1H-indol-3-yl)-2-((*tert*-butoxycarbonyl)amino)propanoic acid (139 mg, 65%) as a white solid that was used without further purification; NMR analysis indicated presence of rotamers (ratio 1:2) which was confirmed by NOE experiments.[6]

Major rotamer: ^{1}H NMR (500 MHz, MeOD) δ 10.67 (bs, 1H, NH), 7.32 (t, J = 8.2 Hz, 1H, ArH), 7.20 – 7.15 (m, 2H, ArH), 6.94 (q, J = 8.8, 7.8 Hz, 1H, ArH), 4.56 (dd, J = 9.5, 5.0 Hz, 1H, CH), 3.71 (dd, J = 14.6, 5.0 Hz, 1H, CH_AH_B), 3.14 (dd, J = 14.6, 9.5 Hz, 1H, CH_AH_B), 1.34 (s, 9H, CH_3).

^{13}C NMR (126 MHz, MeOD) δ 176.2 (CO), 157.9 (CO), 139.4 (C), 127.5 (CH), 126.9 (CH), 126.4 (C), 124.2 (CH), 123.1 (CH), 114.4 (C), 111.8 (C), 80.3 (C), 56.6 (CH), 29.5 (CH_2), 28.6 (CH_3).

Minor rotamer: ^{1}H NMR (500 MHz, MeOD) δ 10.69 (bs, 1H, NH), 7.32 (t, J = 8.2 Hz, 1H, ArH), 7.16 (q, J = 10.7, 9.6 Hz, 2H, ArH), 6.94 (q, J = 8.8, 7.8 Hz, 1H, ArH), 4.68 (dd, J =

[6] Dennis X. Hu, Peter Grice, and Steven V. Ley, J. Org. Chem. 2012, 77, 5198−5202

10.2, 3.5 Hz, 1H, CH), 3.86 – 3.75 (m, 1H, C*H*$_A$H$_B$), 2.93 – 2.78 (m, 1H, CH$_A$*H*$_B$), 1.02 (s, 9H, CH_3).

^{13}C NMR (126 MHz, MeOD) δ 176.9 (CO), 157.4 (CO), 139.6 (C), 127.7 (CH), 127.1 (CH), 126.7 (C), 124.1 (CH), 123.2 (CH), 114.6 (C), 111.9 (C), 80.9 (C), 57.6 (CH), 30.8 (CH_2), 28.0 (CH_3).

HRMS: *m*/*z* calculated for $C_{16}H_{20}BrN_2O_4Na$ [M(^{79}Br)+Na]$^+$: 405.0420; found: 405.0416.

Heck Cross-coupling of *N*-Boc-4-Br-(*S*)-tryptophan with acrylic acid in aqueous conditions.

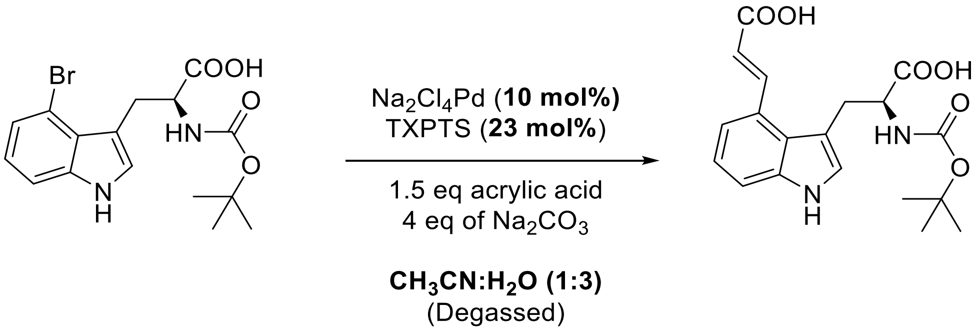

In a 5 mL MW vial, sodium tetrachloropalladate (1.5 mg, 10 µmol, 10 mol%), TXPTS (7.0 mg, 23 µmol, 23 mol%) were purged with argon and stirred at rt for 15 min after adding 1 mL of degassed water/acetonitrile (3:1) mixture, 1 mL. Then, 4-Br-tryptophan (19.16 mg, 0.05 mmol) was added together with Na_2CO_3 (22 mg, 0.2 mmol, 4 eq.) followed by addition of the acrylic acid (6 µl, 0.075 mmol, 1.5 eq). The vial was closed and the reaction mixture was stirred and heated at 90 °C (MW) for 1 hour. After completion, the reaction was cooled down to r.t. was diluted with water (10 ml) and acidified (pH ~2-3) using 0.1 M HCl. The resulting mixture was extracted with ethyl acetate (3×10 mL). The combined organic layers were dried over anhydrous Na_2SO_4 and the solvent was removed *in vacuo* to give (*E*)-3-(3-((*S*)-2-((*tert*-butoxycarbonyl)amino)-2-carboxyethyl)-1H-indol-4-yl)acrylic acid (17.2 mg, 92%) as a yellowish solid that was used without further purification. NMR analysis indicated presence of rotamers (ratio 1:2) which was confirmed by NOE experiments.[1]

^{1}H NMR (500 MHz, MeOD) δ 10.63 (bs, 1H, NH), 8.53 (d, J = 15.6 Hz, 1H, CH=C*H*-Ar), 7.40 – 7.32 (m, 2H, ArH), 7.22 – 7.05 (m, 2H, ArH and CH=C), 6.41 (d, J = 15.6 Hz, 1H, C*H*=CH-Ar), 4.47 (dd, J = 8.9, 5.3 Hz, 1H, CH), 3.52 (dd, J = 14.9, 5.3 Hz, 1H, C*H*$_A$H$_B$), 3.25 (dd, J = 14.9, 8.9 Hz, 1H, CH$_A$*H*$_B$), 1.32 & 1.08 (2×s. 9H, CH_3)

Major rotamer: ^{13}C NMR (126 MHz, MeOD) δ 176.0 (CO), 170.6 (*C*O-CH=CH), 157.9 (CO), 145.5 (CO-CH=*CH*-Ar), 139.1 (C), 128.7 (C), 127.5 (C), 126.7 (CH), 122.4 (CH), 119.3 (CH), 119.1 (CH), 114.5 (CO-*CH*=CH-Ar), 111.7 (C), 80.4 (C), 55.6 (CH), 31.0 (CH_2), 28.7 (CH_3).

Minor rotamer: ^{13}C NMR (126 MHz, MeOD) δ 175.7 (CO), 170.6 (CO-CH=CH), 157.3 (CO), 145.3 (CO-CH=*CH*-Ar), 139.3 (C), 128.7 (C), 127.6 (C), 127.1 (CH), 122.4 (CH), 119.7 (CH), 119.1 (CH), 114.6 (CO-*CH*=CH-Ar), 111.6 (C), 81.0 (C), 56.0 (CH), 31.9 (CH_2), 28.1 (CH_3).

HRMS: *m*/*z* calculated for $C_{19}H_{23}N_2O_6$ [M + H]$^+$: 375.1551; found: 375.1549

One pot Heck Cross-coupling and *N*-Boc deprotection of *N*-Boc-4-Br-tryptophan with acrylic acid in aqueous conditions.

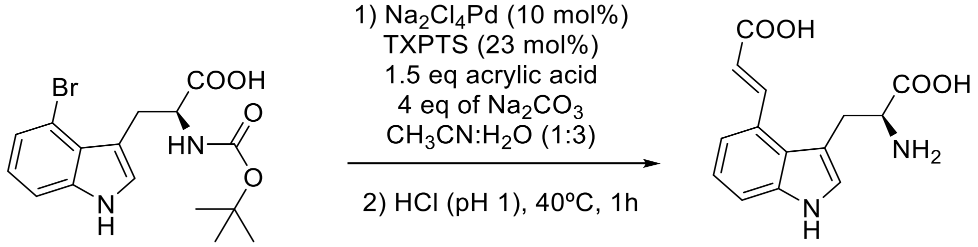

In a 5 mL MW vial, sodium tetrachloropalladate (1.5 mg, 10 µmol, 10 mol%), TXPTS (7.0 mg, 11.5 µmol, 23 mol%) were purged with argon and stirred at r.t. for 15 min after adding 1 mL of degassed water/acetonitrile (3:1) mixture. Then, 4-Br-tryptophan (19.16 mg, 0.05 mmol) was added together with Na_2CO_3 (22 mg, 0.2 mmol, 4 eq.) followed by addition of the acrylic acid (6 µl, 0.075 mmol, 1.5 eq). The vial was closed and the reaction mixture was stirred and heated at 90 °C (MW) for 1 hour. After completion, the reaction was cooled down to room T was diluted with water (10 ml) and acidified (pH ~2-3) using 0.1 M HCl. The resulting mixture was heated at 40 °C for an hour and after the solvent was removed under reduced pressure. The desired product was obtained by purification using gradient reversed phase chromatography (C-18, 12 g) eluting with water-MeOH (5-95% gradient). (*E*)-3-(3-((*S*)-2-amino-2-carboxyethyl)-1H-indol-4-yl)acrylic acid **28** (10.3 mg, 78%) was obtained as a white solid.

^{1}H NMR (500 MHz, MeOD) δ 8.47 (d, J = 15.6 Hz, 1H, CH=C*H*-Ar), 7.44 (d, J = 8.0 Hz, 1H, ArH), 7.39 (d, J = 7.4 Hz, 1H, ArH), 7.32 (s, 1H, CH=C), 7.16 (t, J = 7.7 Hz, 1H, ArH), 6.45 (d, J = 15.6 Hz, 1H, C*H*=CH-Ar), 4.02 (dd, J = 8.9, 5.0 Hz, 1H, CH), 3.74 (dd, J = 15.5, 5.0 Hz, 1H, C$H_A$$H_B$), 3.39 – 3.33 (dd, J = 15.5, 8.9 Hz, 1H, C$H_A$$H_B$).

^{13}C NMR (126 MHz, MeOD) δ 173.4 (CO), 170.82 (CO-CH=CH), 145.0 (CO-CH=*CH*-Ar), 139.4 (C), 128.7 (C), 127.3 (CH), 126.4 (C), 122.9 (CH), 120.4 (C-H), 119.3 (C-H), 114.7 (C CO-*CH*=CH-Ar), 110.02 (C), 55.9 (CH), 30.5 (CH_2).

HRMS: *m*/*z* calculated for $C_{14}H_{15}N_2O_4$ $[M + H]^+$: 275.1026; found: 275.1036

General protocol for Heck Cross-coupling of *N*-Boc protected halo-aminoacids with acrylic acid in aqueous media.

In a 5 mL MW vial, sodium tetrachloropalladate (1.5 mg, 10 µmol, 10 mol%), TXPTS (7.0 mg, 23 µmol, 23 mol%) were purged with argon and stirred at r.t. for 15 min after adding 1 mL of degassed water/acetonitrile (3:1) mixture. Then, appropriate *N*-Boc protected halo-aminoacid (19.2 mg, 0.05 mmol) was added together with Na_2CO_3 (22 mg, 0.2 mmol, 4 eq.) followed by addition of the acrylic acid (6 µl, 0.075 mmol, 1.5 eq). The vial was closed and the reaction mixture was stirred and heated at 90 °C (MW) for 1 hour. After completion, the reaction was cooled down to r.t. was diluted with water (10 ml) and acidified (pH ~2-3) using 0.1 M HCl. The resulting mixture was extracted with ethyl acetate (3×10 mL). The combined organic layers were dried over anhydrous Na_2SO_4 and the solvent was removed *in vacuo* to give the corresponding Heck product without the need for further purification.

(*E*)-3-(4-((*S*)-2-((*tert*-butoxycarbonyl)amino)-2-carboxyethyl)phenyl)acrylic acid

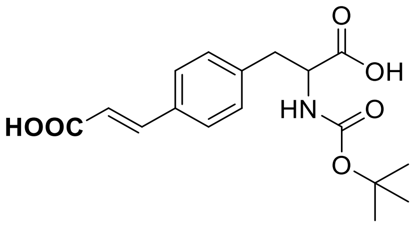

The above procedure afforded 33 mg (98% from *N*-Boc-4-Br-phenylalanine) of the desired product as a white solid. NMR analysis indicated presence of rotamers (ratio 1:4) which was confirmed by NOE experiments.

^{1}H NMR (500 MHz, MeOD) Mixture of rotamers: δ 7.65 (d, J = 16.0 Hz, 1H, CH=C*H*-Ar), 7.52 (d, J = 8.0 Hz, 2H, ArH), 7.29 (d, J = 8.0 Hz, 2H, ArH), 6.45 (d, J = 16.0 Hz, 1H, C*H*=CH-Ar), 4.37 (dd, J = 9.2, 4.9 Hz, 1H, CH), 3.19 (dd, J = 13.8, 4.9 Hz, 1H, CH_AH_B), 2.93 (dd, J = 13.8, 9.3 Hz, 1H, CH_AH_B), 1.37 (s, 7H), 1.32 (s, 2H)

Major rotamer: ^{13}C NMR (126 MHz, MeOD) δ 175.1 (CO), 170.4 (CO-CH=CH), 157.8 (CO), 146.1 (CH=*CH*-Ar), 141.5 (C), 134.3 (C), 131.0 (2CH), 129.2 (2CH), 118.8 (*CH*=CH-Ar), 80.5 (C), 56.1 (CH), 38.5 (CH_2), 28.6 (CH_3).

Minor rotamer: ^{13}C NMR (126 MHz, MeOD) δ 175.1 (CO), 170.4 (*C*O-CH=CH), 157.1 (CO), 146.1 (CH=*CH*-Ar), 141.5 (C), 134.3 (C), 131.1 (2CH), 129.2 (2CH), 118.9 (*CH*=CH-Ar), 81.4 (C), 57.5 (CH), 39.0 (CH_2), 28.4 (CH_3).

HRMS (FTMS +p ESI) *m/z* $C_{17}H_{21}NNaO_6$ $[M+Na]^+$ calculated 358.1261, found 358.1256.

(*E*)-3-((*S*)-2-(2-((*tert*-butoxycarbonyl)amino)-2-carboxyethyl)phenyl)acrylic acid

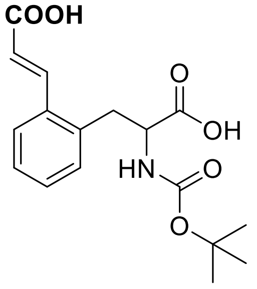

The above procedure afforded 31 mg (92% from *N*-Boc-2-Br-phenylalanine) of the desired product as a white solid. NMR analysis indicated presence of rotamers (ratio 1:2) which was confirmed by NOE experiments.

^{1}H NMR (500 MHz, MeOD) δ 8.09 (d, J = 15.8 Hz, 1H, CH=C*H*-Ar), 7.66 (d, J = 7.3 Hz, 1H, ArH), 7.48 – 7.15 (m, 3H, ArH), 6.42 (d, J = 15.7 Hz, 1H, C*H*=CH-Ar), 4.31 (dd, J = 9.4, 4.9 Hz, 1H, CH), 3.39 (dd, J = 14.1, 4.9 Hz, 1H, CH_AH_B), 3.02 (dd, J = 14.1, 9.5 Hz, 1H, CH_AH_B), 1.34 (s, 6H, CH_3) and 1.24 (s, 3H, CH_3).

Major rotamer: ^{13}C NMR (126 MHz, MeOD) δ 175.0 (CO), 170.3 (*C*O-CH=CH), 157.6 (CO), 143.4 (CH=*CH*-Ar), 138.4 (C), 134.9 (C), 132.2 (CH), 131.0 (CH), 128.5 (CH), 127.8 (CH), 121.2 (*CH*=CH-Ar), 80.5 (C), 56.0 (CH), 36.0 (CH_2), 28.6 (CH_3).

Minor rotamer: ^{13}**C NMR (126 MHz, MeOD)** δ 174.9 (CO), 170.3 (*C*O-CH=CH), 157.6 (CO), 143.1 (CH=*CH*-Ar), 138.5 (C), 134.9 (C), 132.6 (CH), 131.1 (CH), 128.6 (CH), 127.8 (CH), 121.3 (*CH*=CH-Ar), 93.2 (C), 80.5 (C), 57.2 (CH), 36.8 (CH_2), 28.3 (CH_3).

HRMS (FTMS +p ESI) *m/z* $C_{17}H_{21}NNaO_6$ $[M + Na]^+$ calculated 358.1261, found 358.1255.

Br-Pacidamycin-D

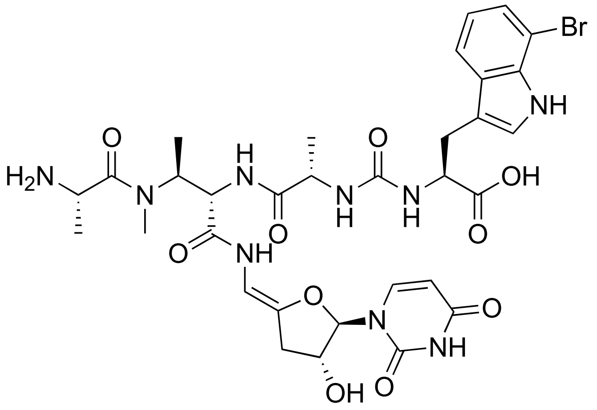

Due to limited amount of material, full NMR characterisation could not be performed. The sample was submitted for HSQC NMR and stacked with wild type pacidamycin D. Closer investigation of the aromatic region showed the downfield shifting of protons at 7.14 and 7.09 ppm to 7.24 and 7.29 ppm respectively. Furthermore, the wild type pacidamycin D had a proton present at 7.34 ppm. After the insertion of the bromine atom, this signal disappeared.

Table S-2: Comparison of ^{1}H and ^{13}C peaks extracted from HSQC NMR spectra for Pacidamycin D and Br-pacidamycin D*

Pacidamycin D		Br pacidamycin D	
**^{1}H**	**^{13}C**	**^{1}H**	**^{13}C**
1.19	12.1	1.20	12.2
1.25	13.8	1.23	13.8
1.27	16.8	1.28	16.8
1.35	14.9	1.38	14.9
1.43	15.5	1.43	15.5
2.60	33.5	2.61	33.4
2.63	33.6	2.63	33.6
2.82	27.0	2.84	27.0
2.84	29.1	2.89	29.1
3.21	27.7	3.20	27.7
3.28	27.5	3.29	27.8
4.60	53.5	4.60	53.4
4.70	54.8	4.71	54.9
5.72	101.9	5.74	101.6
5.98	96.3	5.98	96.6
6.05	94.1	6.06	93.9
7.02	118.5	6.97	119.6
**7.09**	**120.1**	**7.29**	**123.4**
**7.14**	**123.2**	**7.24**	**124.9**
**7.35**	**110.7**	**Absent due to bromination**	**Absent due to bromination**
7.59	118.1	7.60	117.6

*Significant changes are highlighted in bold.

MS (ESI): 790 (100) [M(^{79}Br) + H]$^{+}$, 792 (100) [M(^{81}Br) + H]$^{+}$; **HRMS (FTMS +p ESI):** *m/z* calculated for $C_{32}H_{41}BrN_9O_{10}^{+}$ [M(^{79}Br + H)]$^{+}$: 790.2154; found: 790.2150.

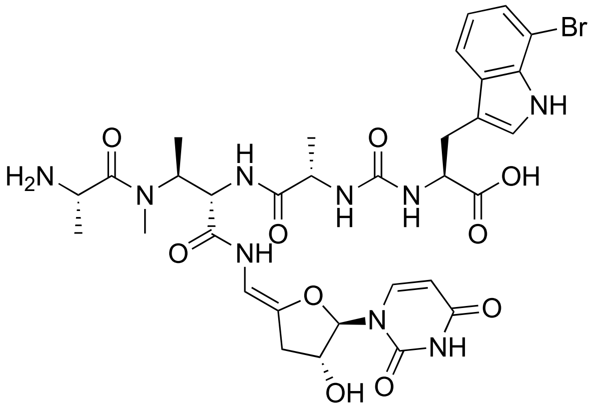

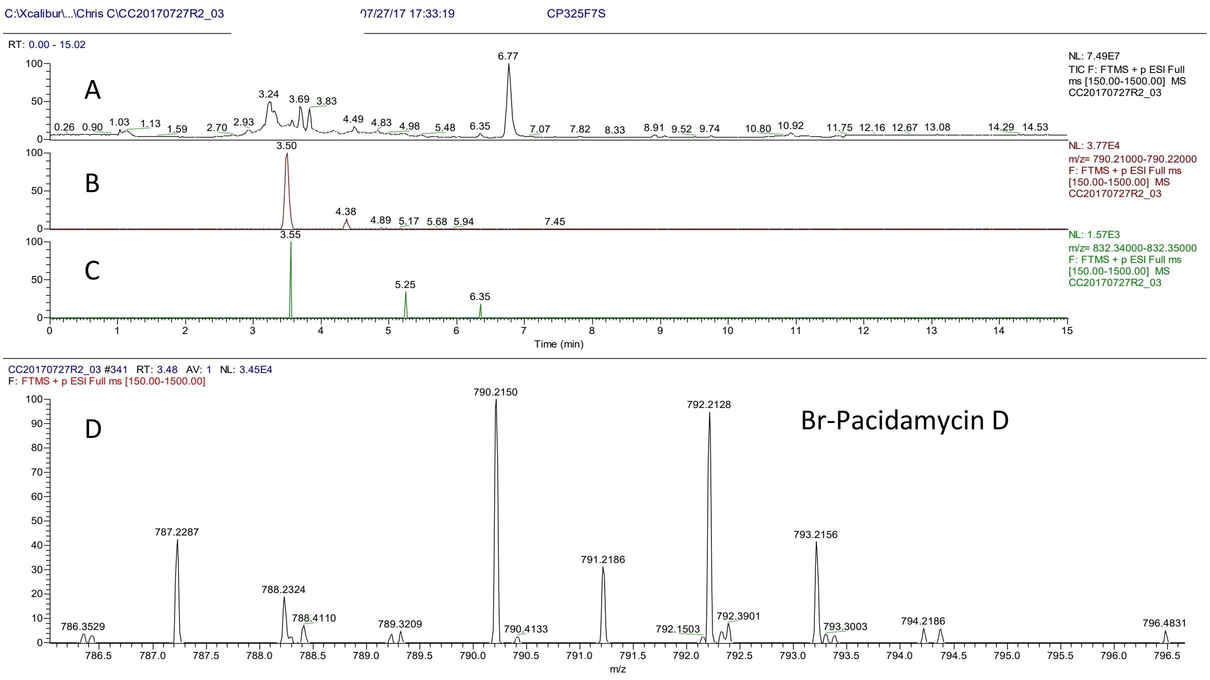

Fig. S1. Mass spectrometry analysis of Br-pacidamycin culture extracts grown in ISP2. A) TIC of culture extract, **B**) EIC for Br-pacidamycin D (*m/z*= 790, 792 for ^{79}Br and ^{81}Br respectively), **C**) EIC showing no Heck product before reaction, **D)** Extracted mass spectrum showing the presence of Br-pacidamycin D.

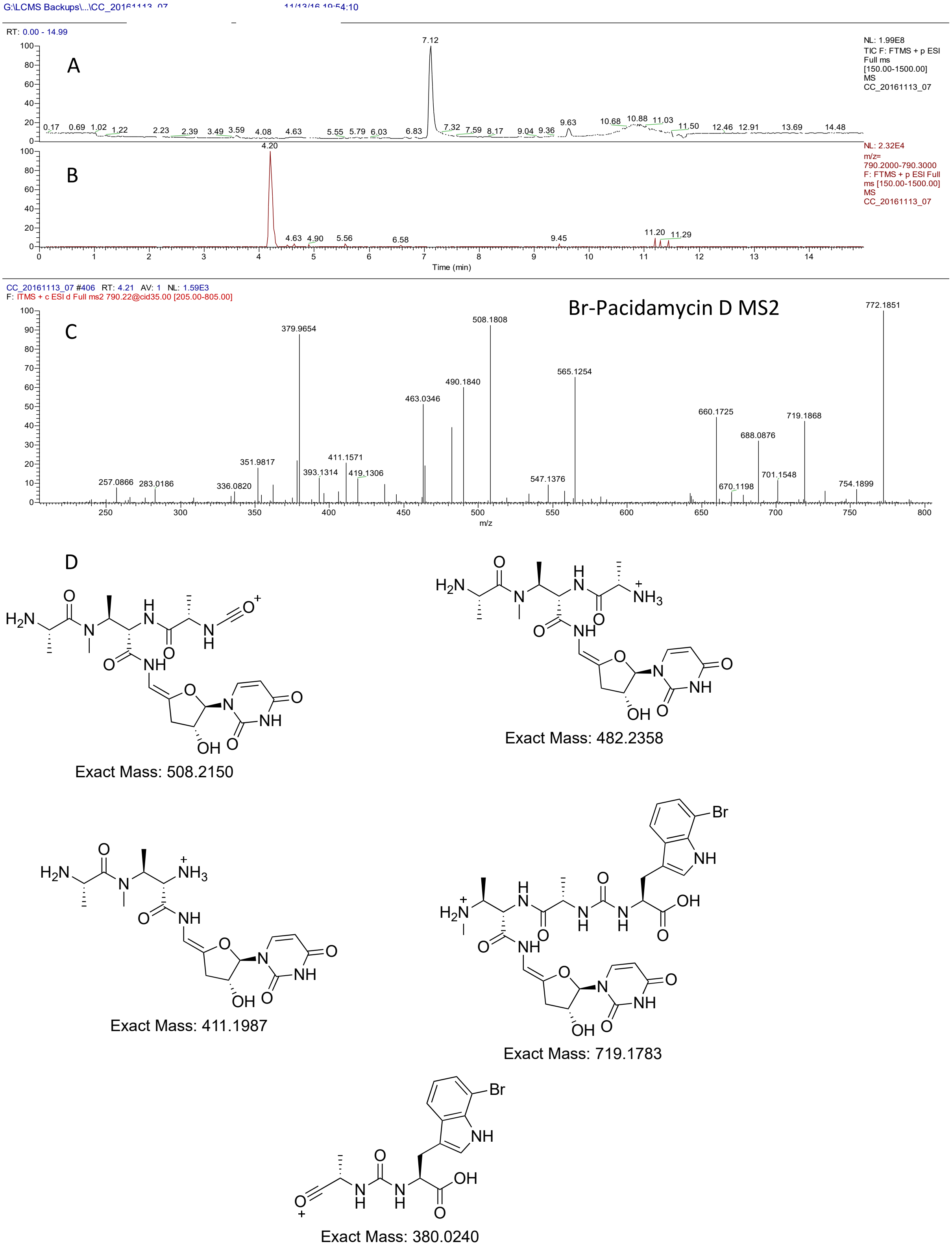

Fig S2. MS2 fragmentation of Br-pacidamycin D for *m/z* 790 (for ^{79}Br). A) TIC of culture, **B**) EIC for Br-pacidamycin D (*m/z*= 790, 792 for ^{79}Br and ^{81}Br respectively). **C**) Extracted MS2 spectrum of Br-pacidamycin D. **D**) Observed fragments from MS2 spectrum of Br-pacidamycin D

Heck cross-coupling of Br-pacidamycin D

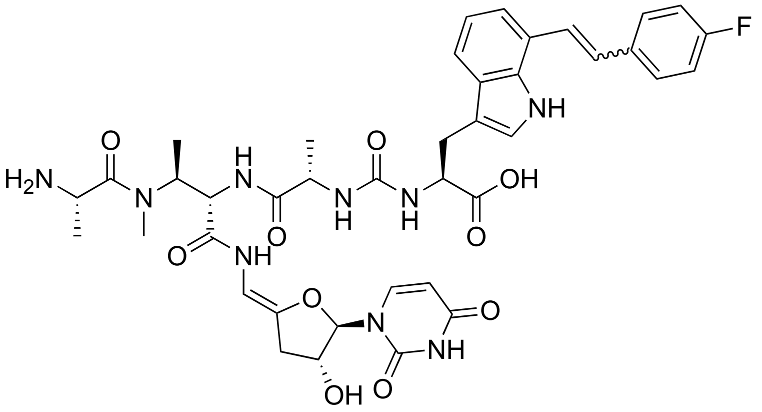

To a solution of the purified 7-Br-pacidamycin D (~0.5 mg) in degassed water-acetonitrile (3:1), sodium tetrachloropalladate (10 mol%), TXPTS (23 mol%) (from stock solution in degassed water-acetonitrile (3:1) 10 mM) were added followed by Na_2CO_3 (4 eq.) and 4-fluorostyrene (5 eq) were added. The vial was closed and the reaction mixture was stirred and heated at 90 °C (MW) for 2 hour. After completion, the reaction was cooled down to rt was diluted with water (10 ml) and acidified (pH ~2-3) using 0.1 M HCl. The resulting mixture was extracted with ethyl acetate (3×10 mL). Complete conversion was observed by LC-HRMS analysis of the crude reaction mixture. Attempted purification by HPLC was not successful to isolate desired product, which may be due to very low quantities of product.

MS (ESI) 832 (100) $[M + H]^+$; **HRMS**: *m/z* calculated for $C_{40}H_{47}FN_9O_{10}^+$ $[M + H]^+$: 832.3424; found: 832.3422.

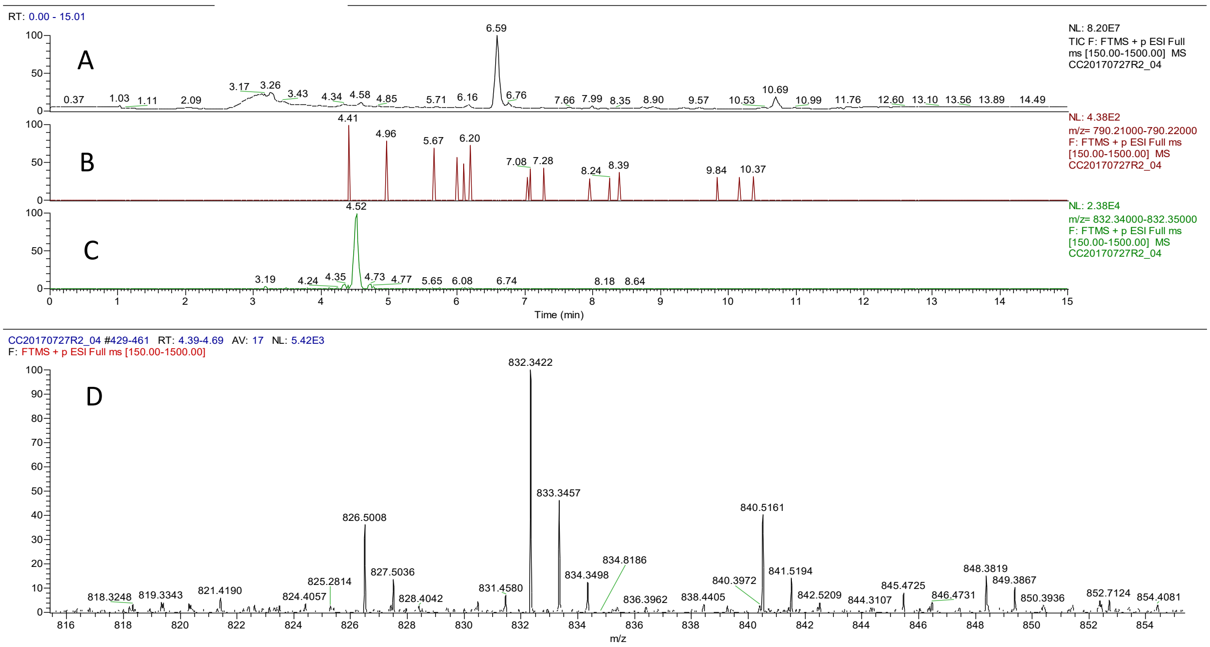

Fig S3. LCMS chromatogram showing the total consumption of Br-pacidamycin D and formation of the Heck cross-coupling product. A) TIC of crude Heck reaction mixture of Br-pacidamycin D, **B**) EIC for Br-pacidamycin D (*m/z*= 790, 792 for ^{79}Br and ^{81}Br respectively) showing complete conversion, **C**) EIC showing formation of desired Heck product (*m/z*= 832), **D)** Extracted mass spectrum showing the presence of 4-fluorostyryl derivative of pacidamycin D.

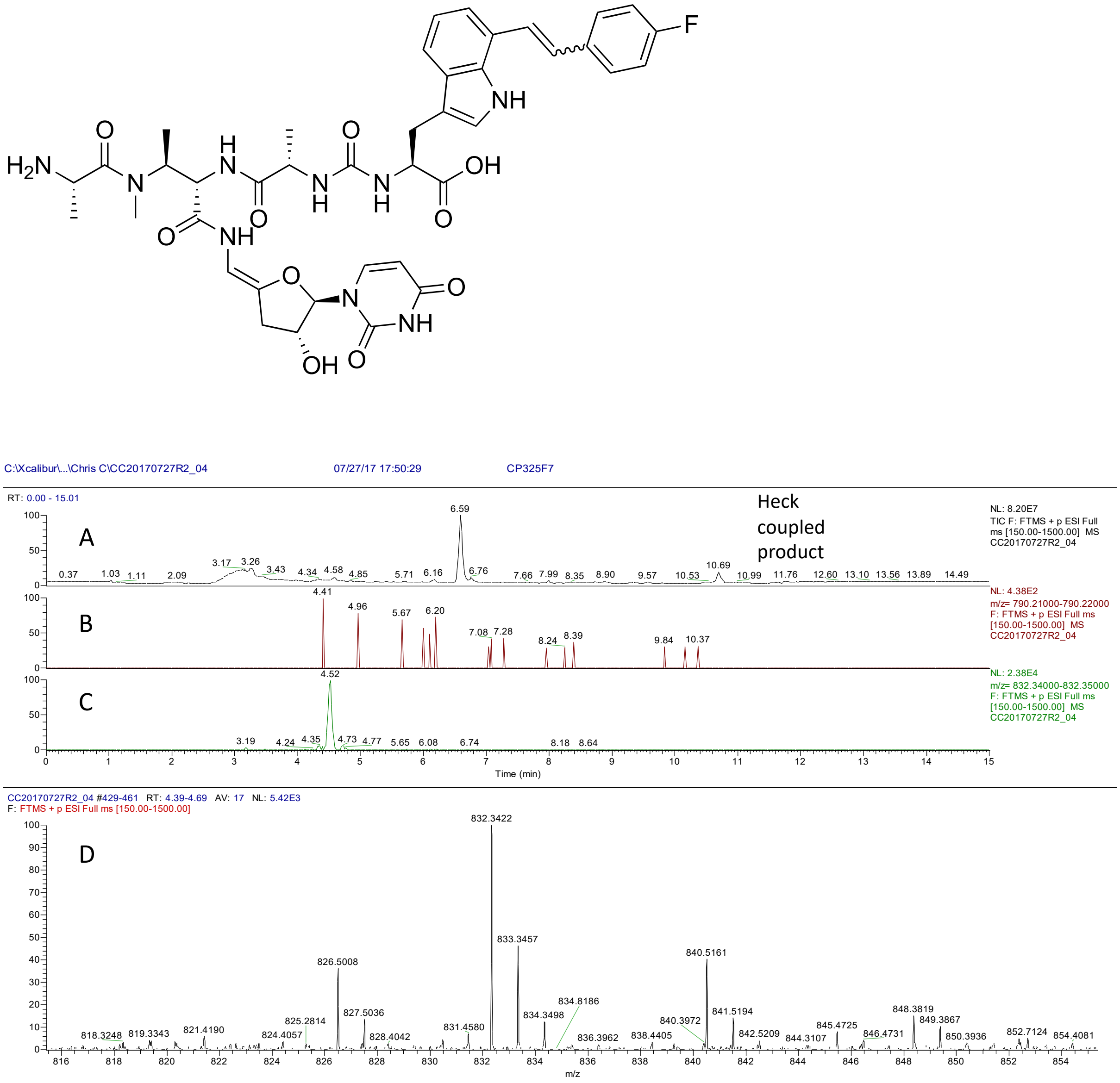

Fig. S4. Mass spectrometry analysis of Heck cross coupling of Br-pacidamycin D A) TIC of reaction mixture, **B**) EIC depicting complete consumption of Br-pacidamycin D, **C**) EIC showing Heck product, **D)** Extracted mass spectrum at showing the presence of Heck cross coupled pacidamycin D

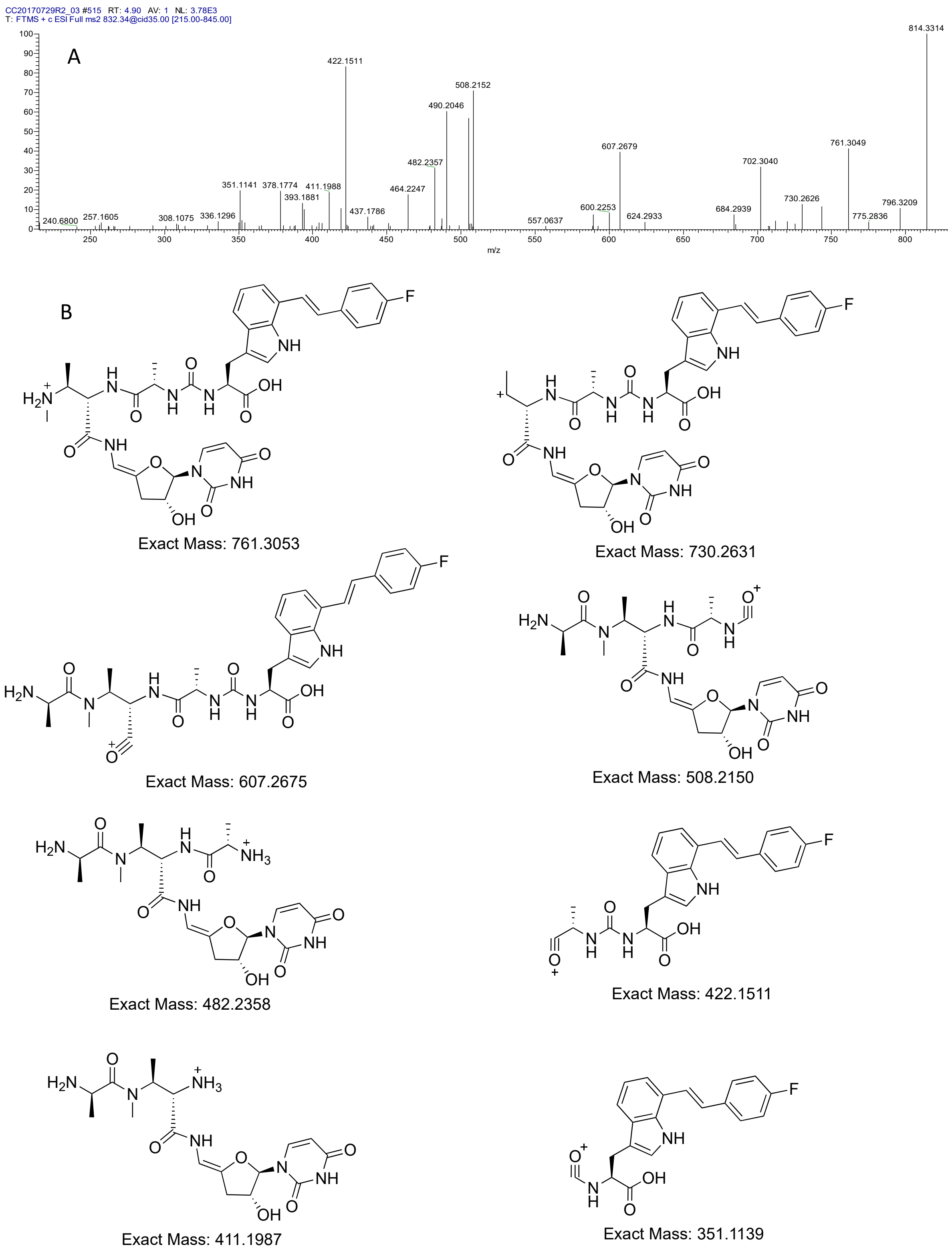

Fig. S5. MS2 fragmentation of Heck cross coupled pacidamycin D for *m/z* 832. A) Extracted MS2 spectrum of Heck cross coupled pacidamycin. **B**) Observed fragments from MS2 spectrum of Heck cross coupled pacidamycin

NMR spectra of purified products.

(*E*)-3-(1H-indol-5-yl)acrylic acid

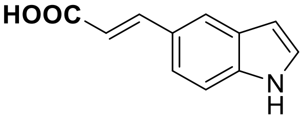

^{1}H NMR (400 MHz, MeOD)

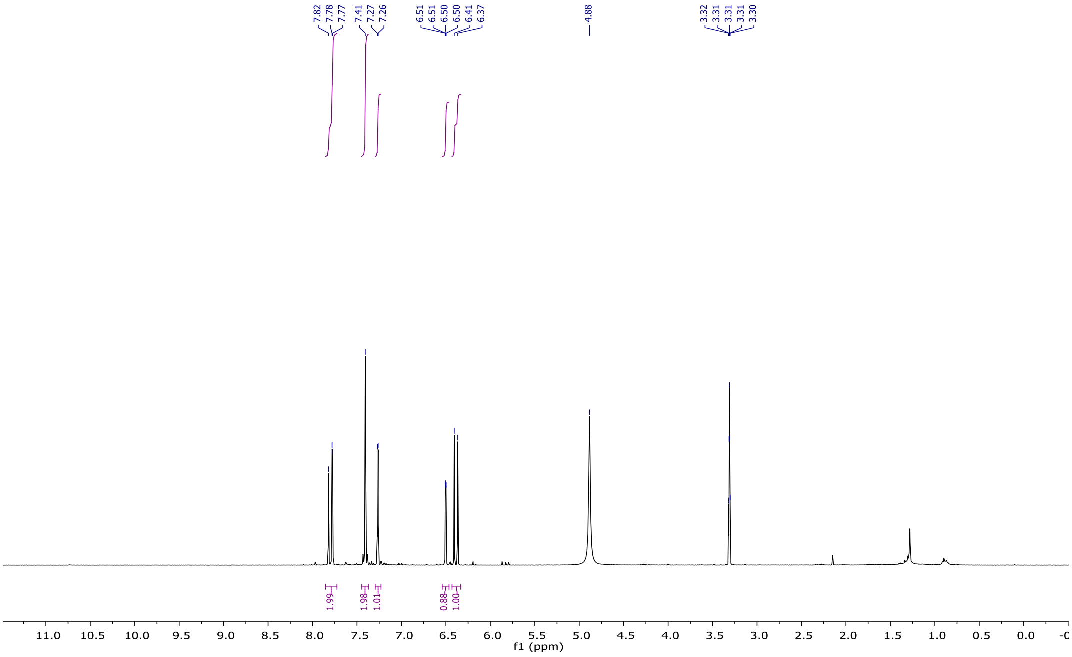

^{13}C NMR (126 MHz, MeOD)

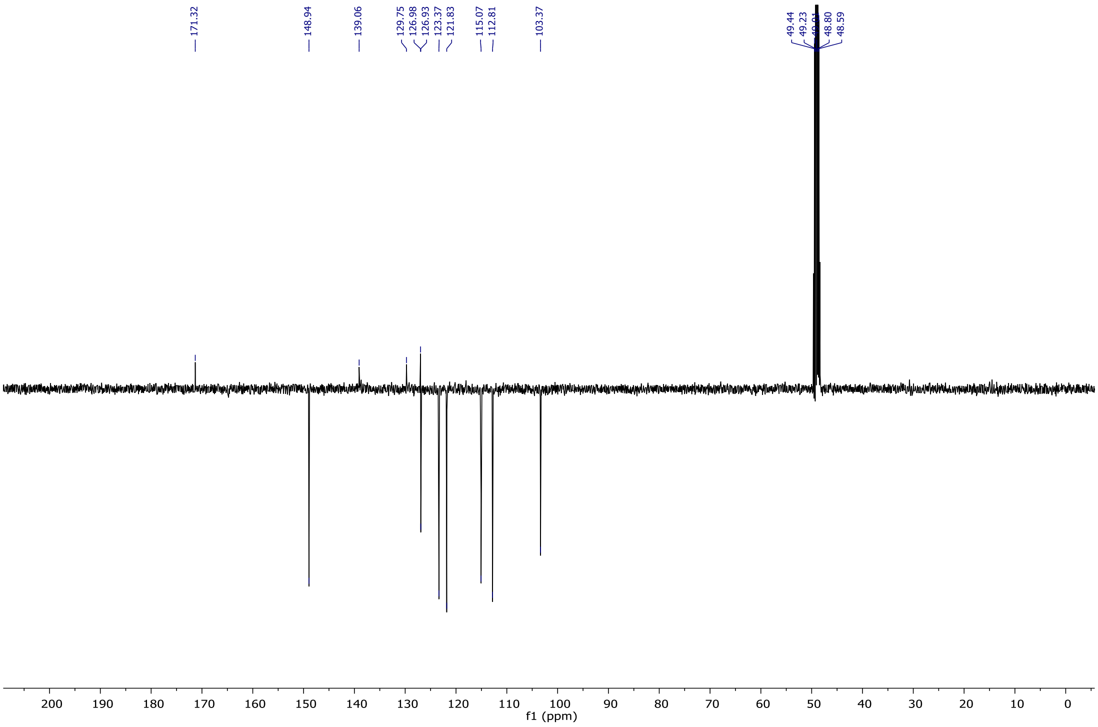

Ethyl (*E*)-3-(1H-indol-5-yl)acrylate

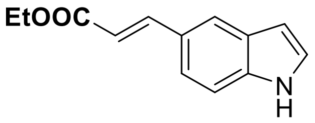

^{1}H NMR (400 MHz, $CDCl_3$)

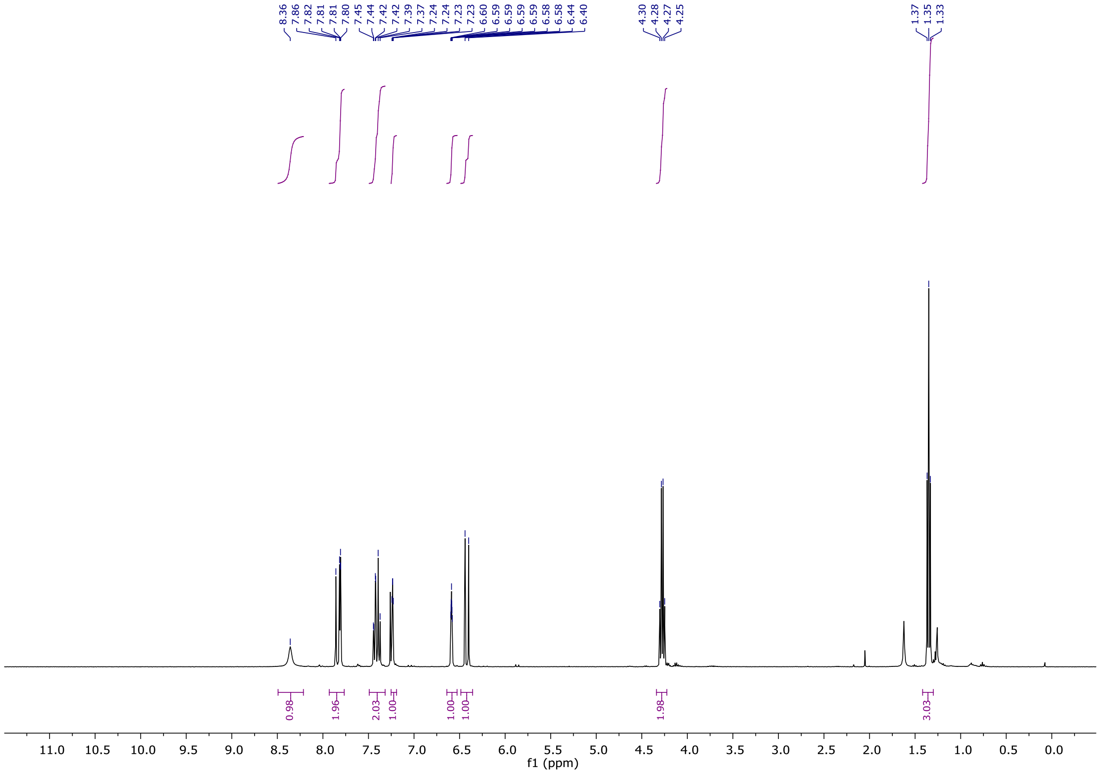

^{13}C NMR (101 MHz, $CDCl_3$)

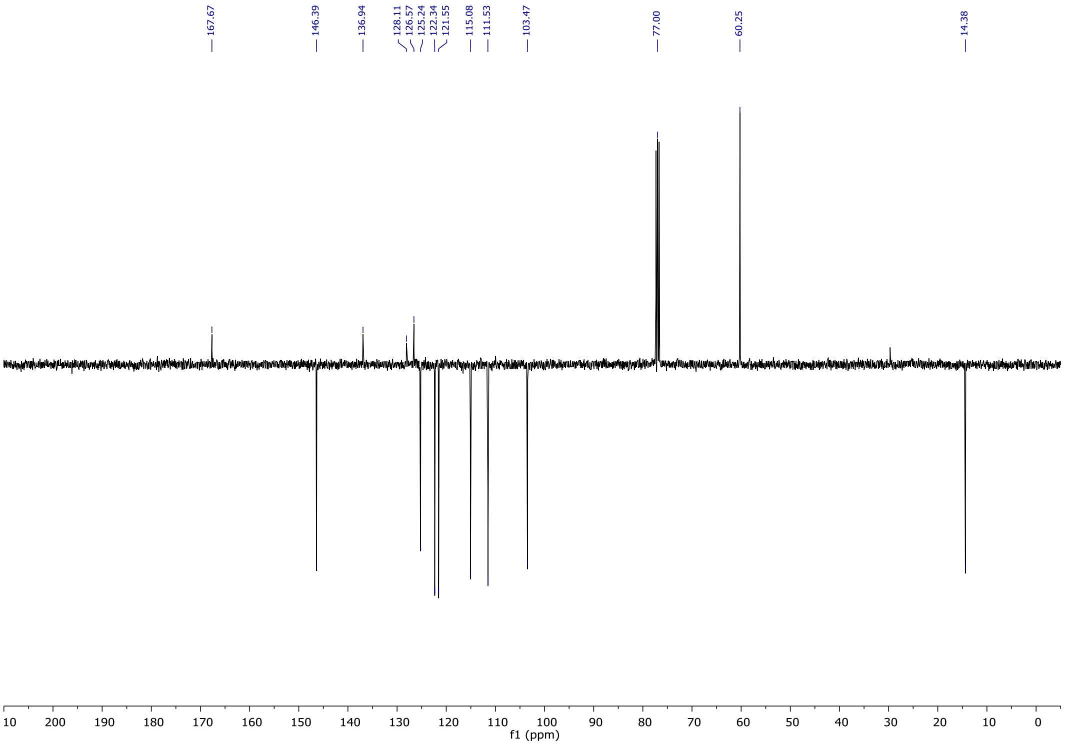

(*E*)-3-(1H-indol-7-yl)acrylic acid

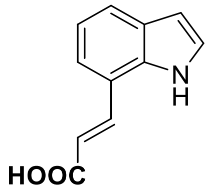

^{1}H NMR (400 MHz, MeOD)

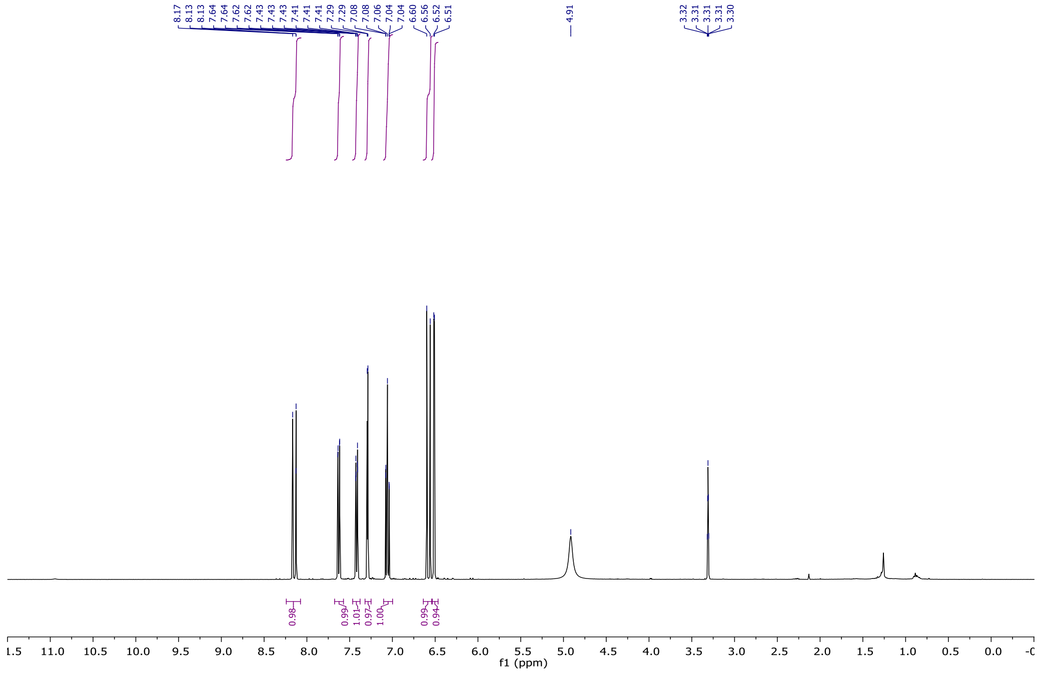

^{13}C NMR (101MHz, MeOD)

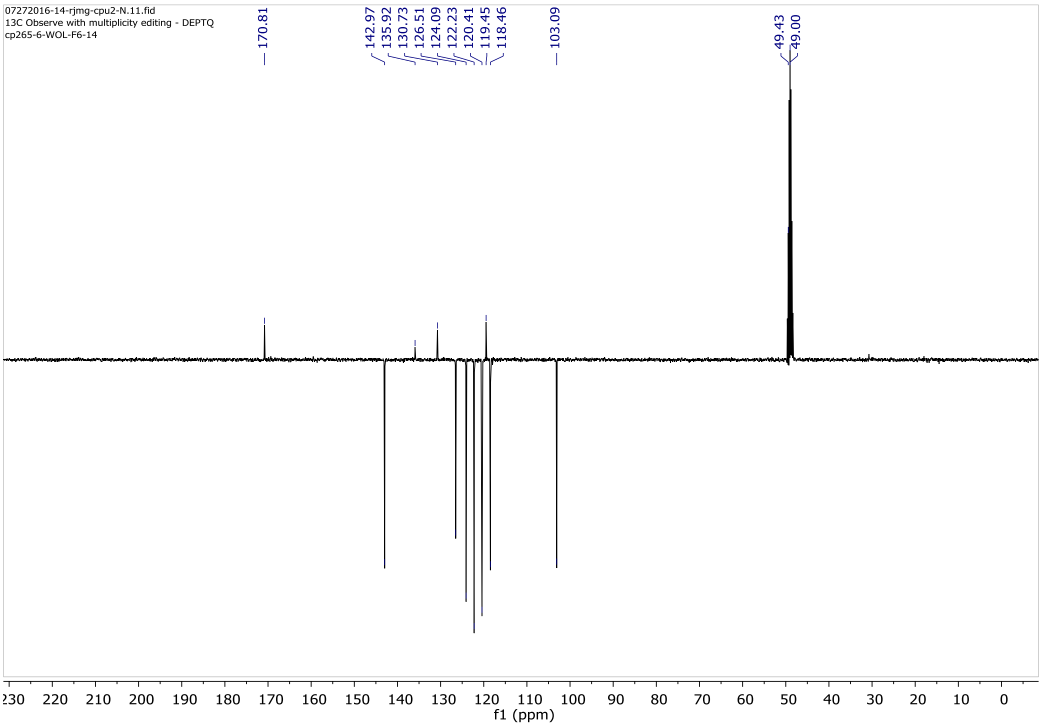

(*E*)-4-(1H-indol-5-yl)but-3-en-2-one

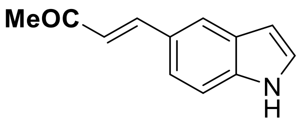

^{1}H NMR (400 MHz, CDCl$_3$)

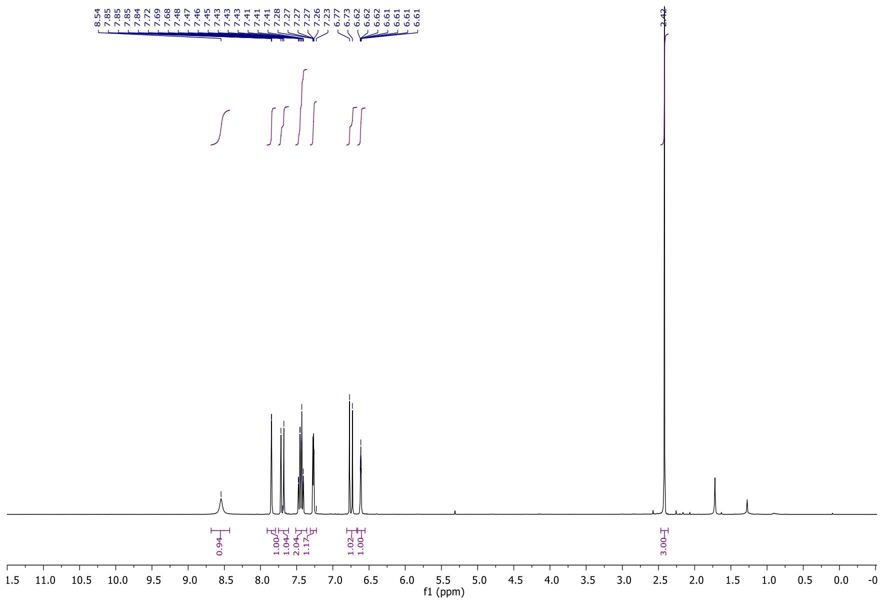

^{13}C NMR (101 MHz, CDCl$_3$)

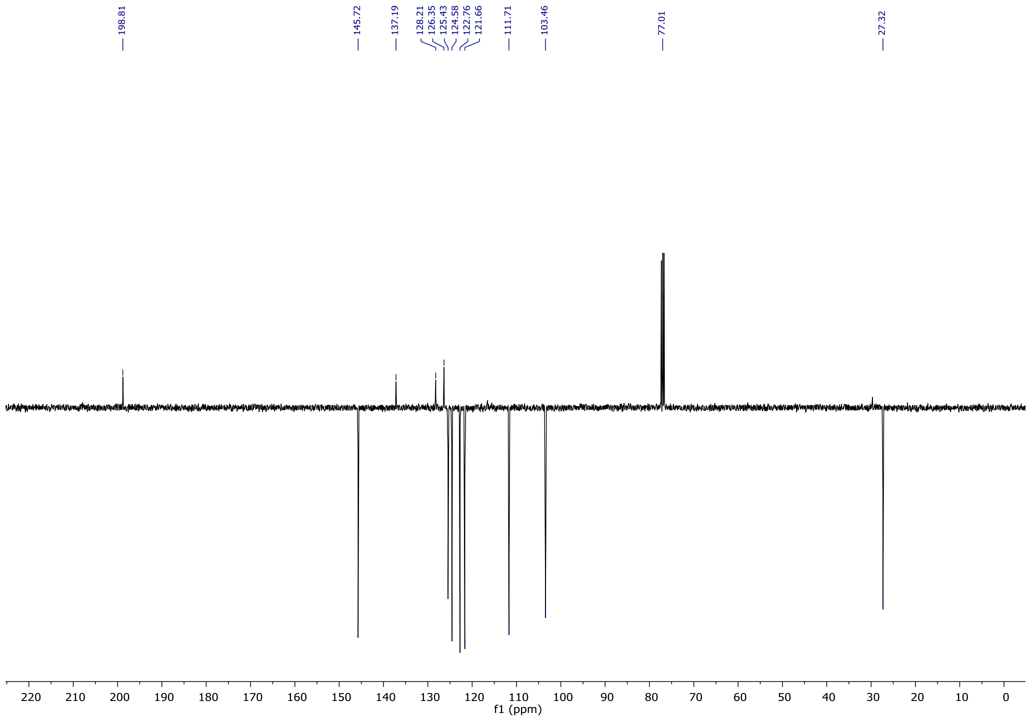

(*E*)-3-(1H-indol-5-yl)acrylonitrile

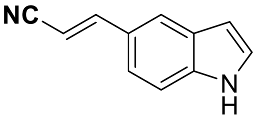

^{1}H NMR (400 MHz, MeOD)

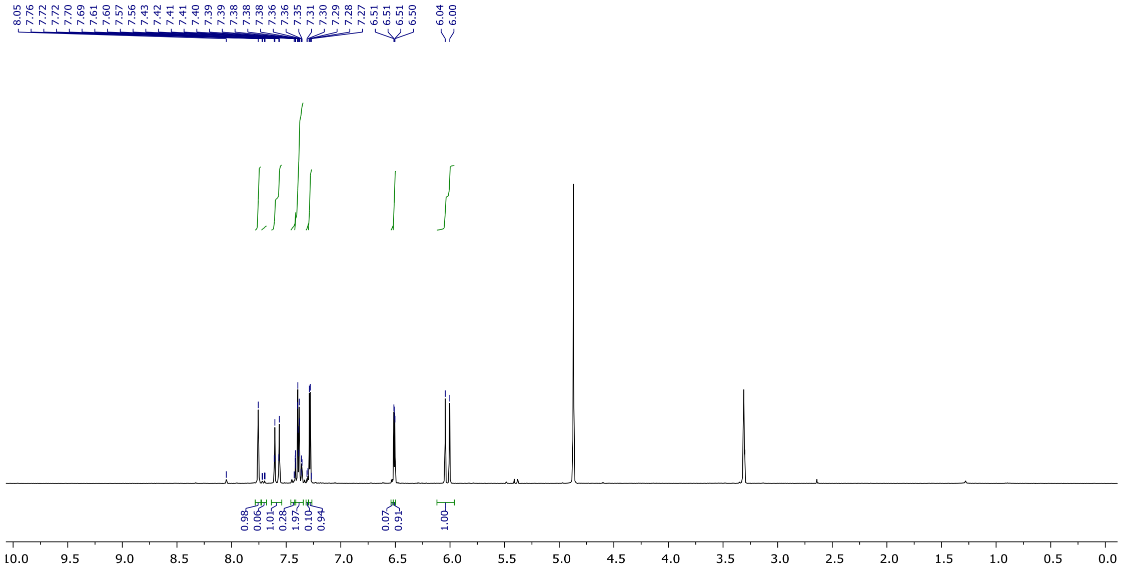

^{13}C NMR (101 MHz, MeOD)

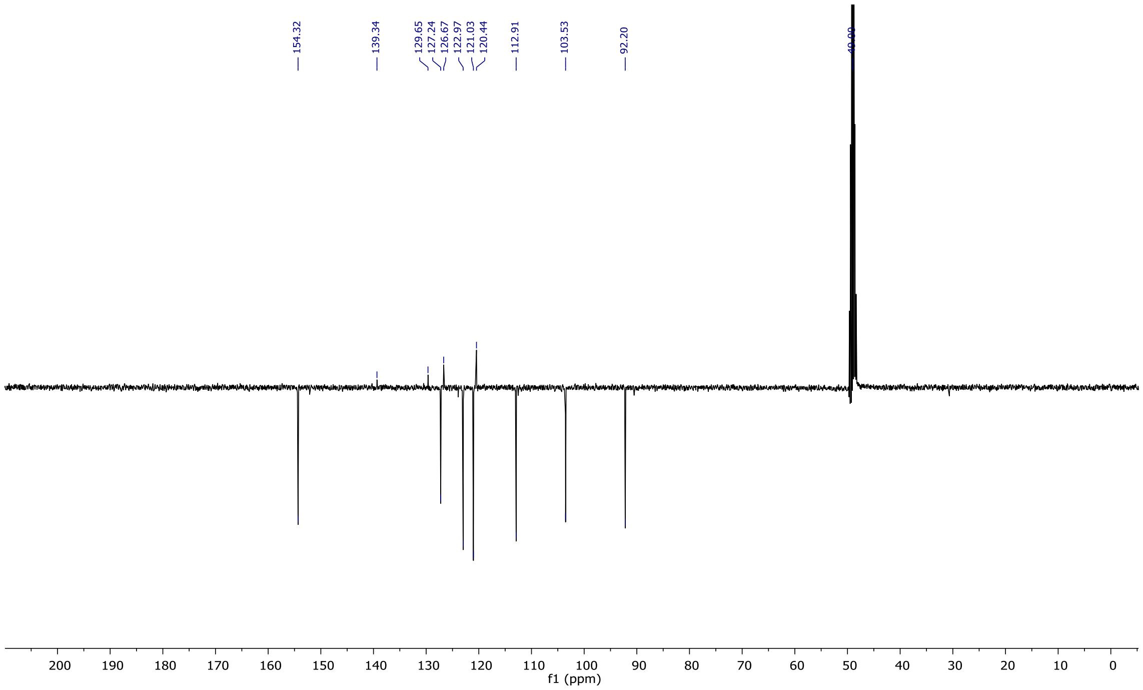

(*E*)-3-(1H-indol-6-yl)acrylic acid

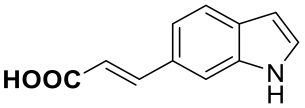

^{1}H NMR (400 MHz, MeOD)

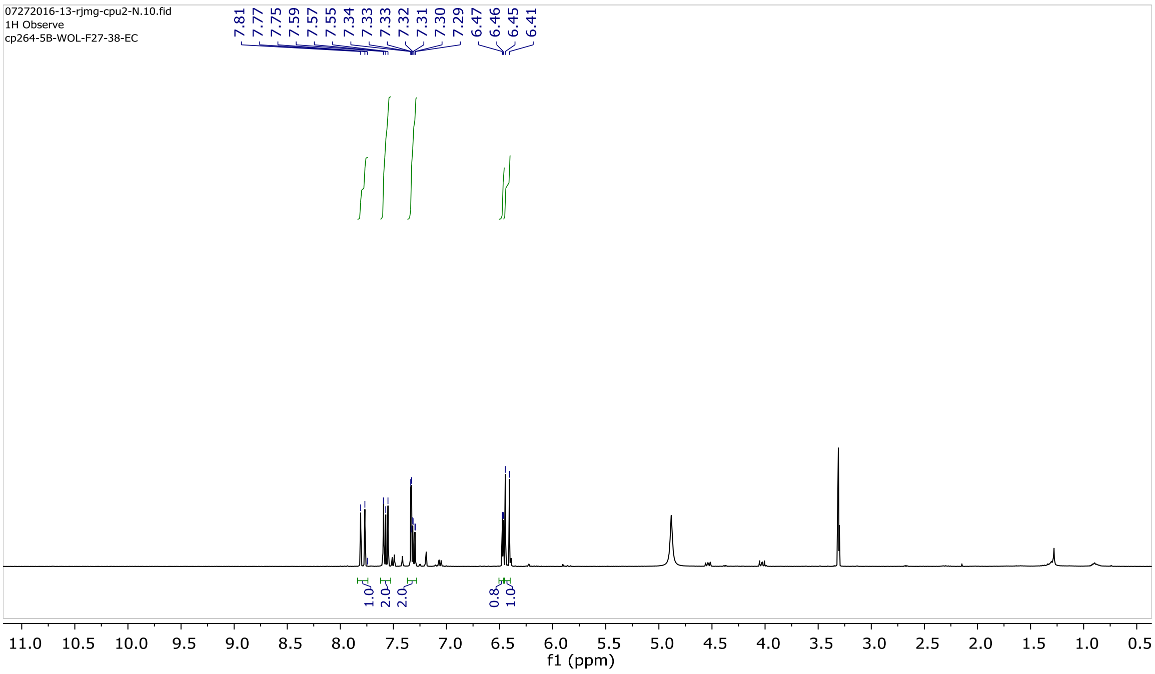

^{13}C NMR (101 MHz, MeOD)

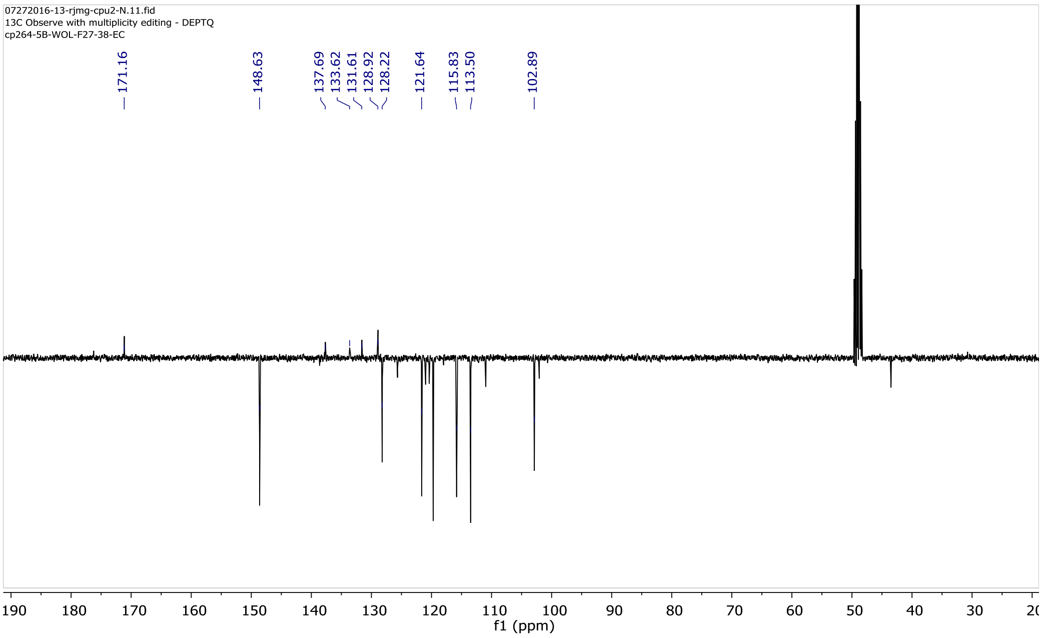

(E)-3-(3-((S)-2-amino-2-carboxyethyl)-1H-indol-5-yl)acrylic acid

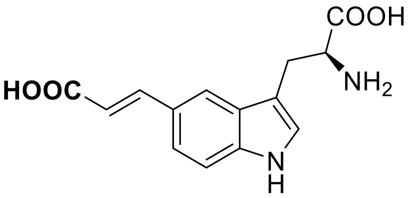

^{1}H NMR (400 MHz, MeOD)

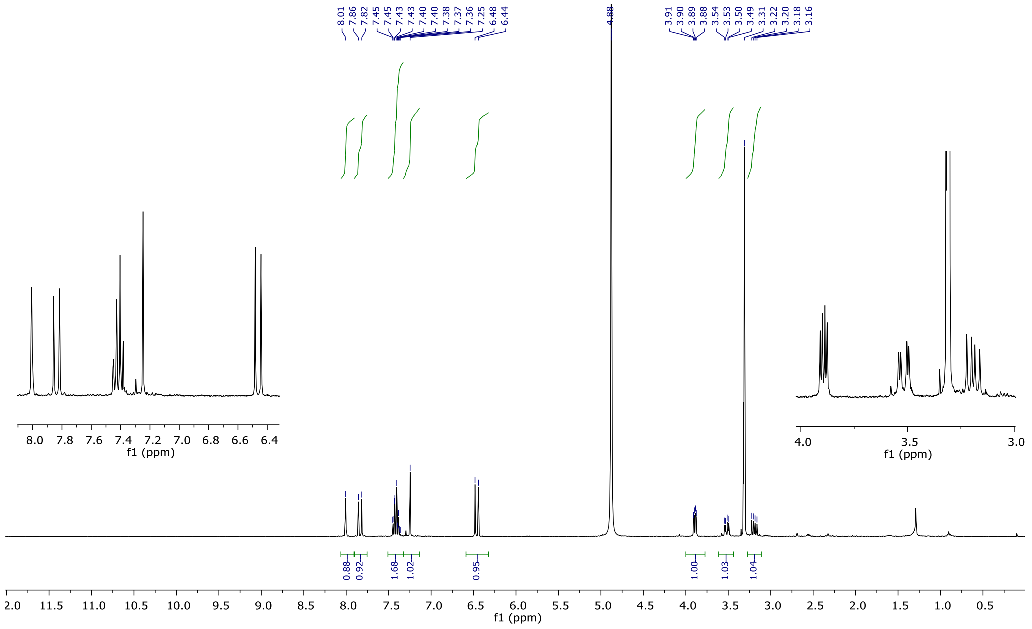

^{13}C NMR (126 MHz, DMSO-d_6)

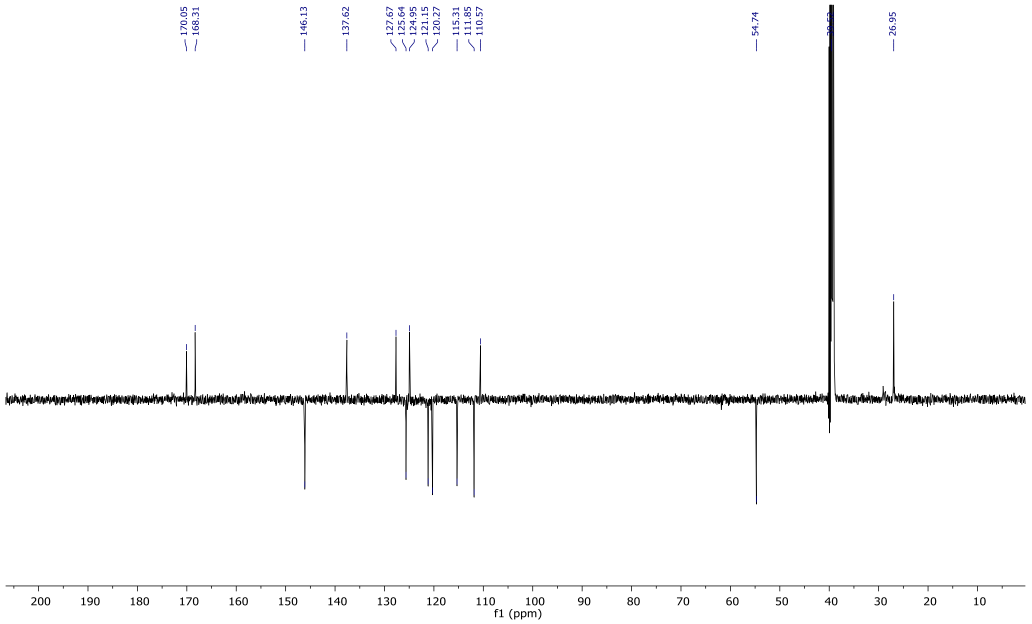

(*E*)-3-(3-((*S*)-2-amino-2-carboxyethyl)-1H-indol-6-yl)acrylic acid

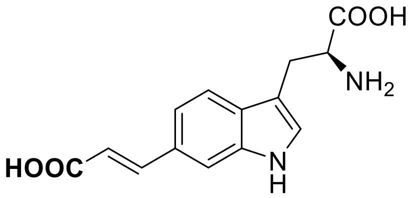

^{1}H NMR (500 MHz, MeOD)

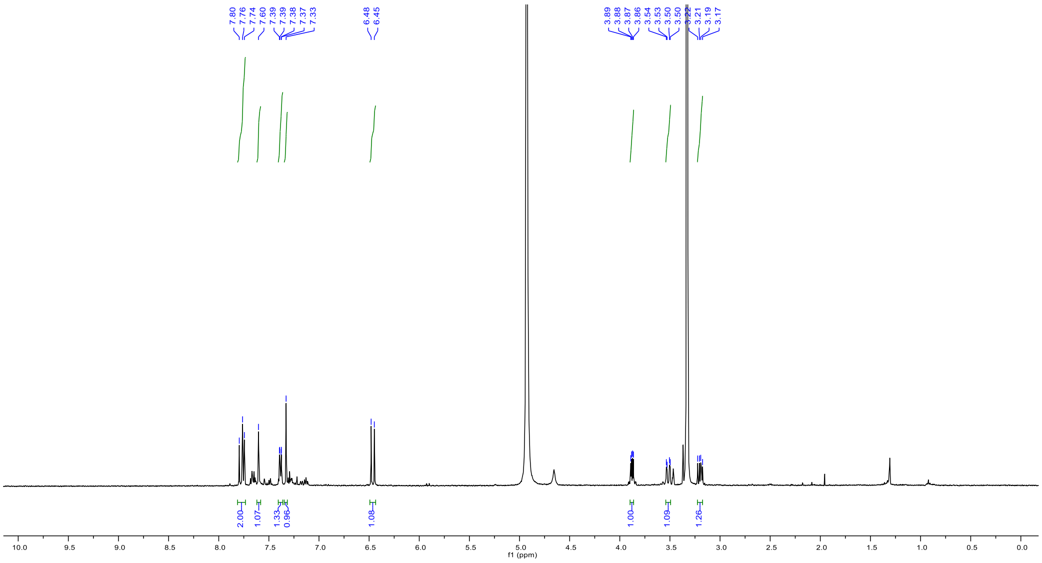

^{13}C NMR (126 MHz, MeOD)

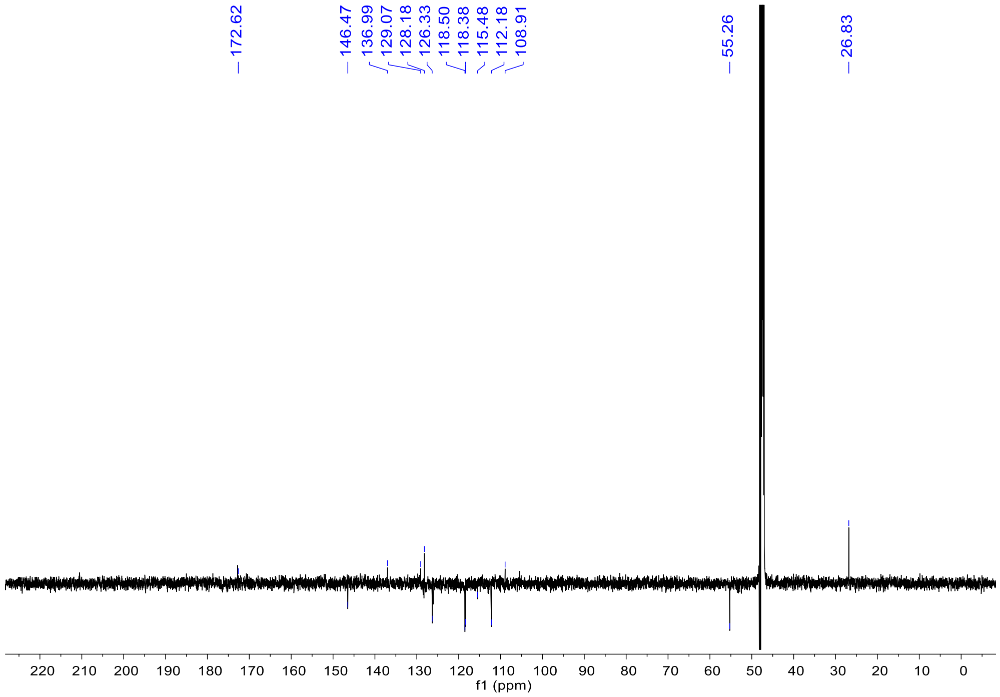

COSY NMR (500 MHz, MeOD)

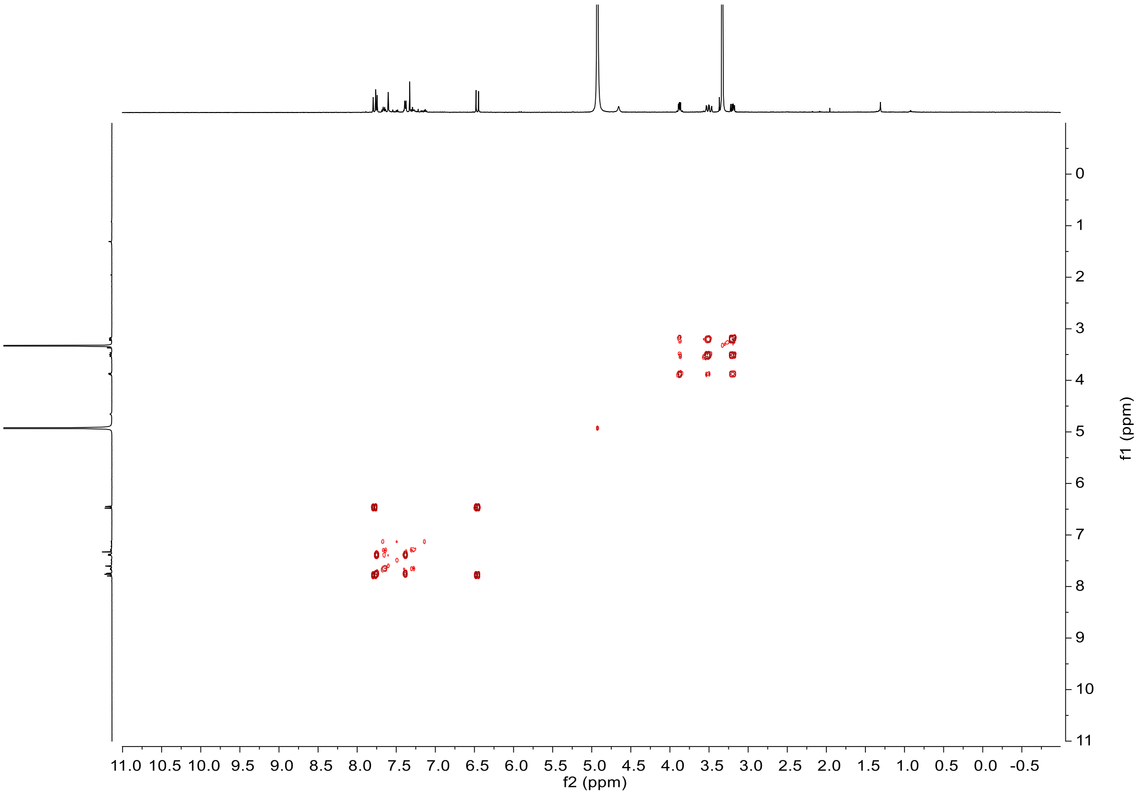

HSQC NMR (500 MHz, MeOD)

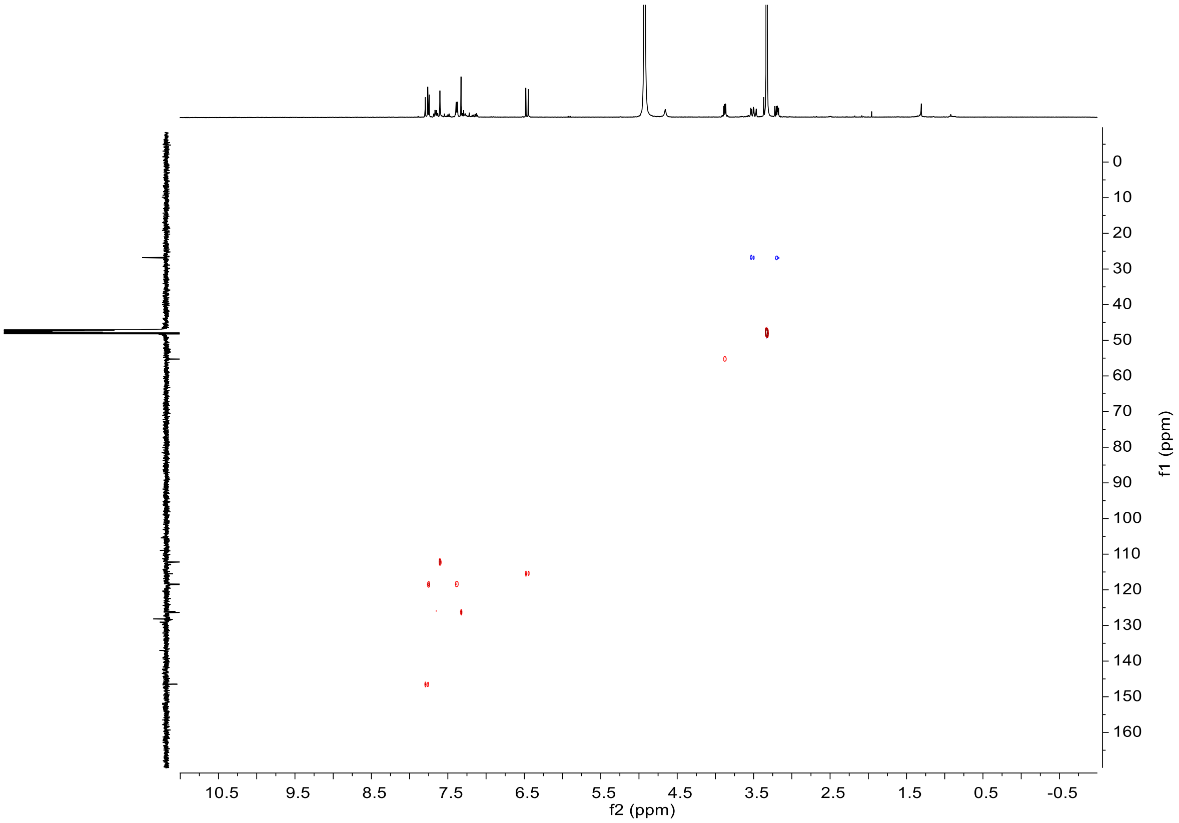

HMBC NMR (500 MHz, MeOD)

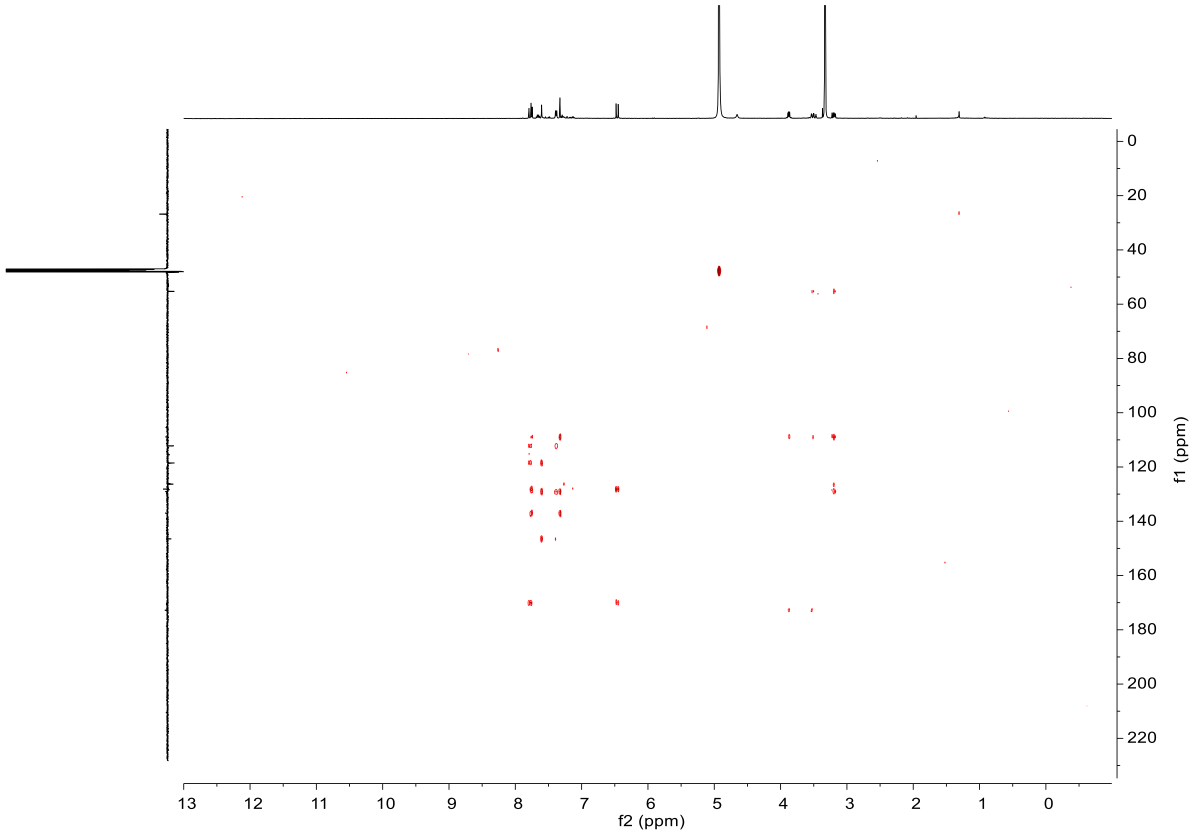

(*E*)-3-(3-((*S*)-2-amino-2-carboxyethyl)-1H-indol-7-yl)acrylic acid

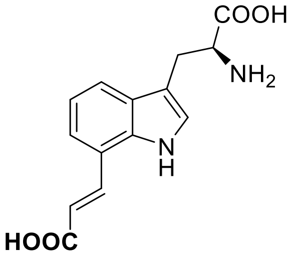

^{1}H NMR (400 MHz, MeOD)

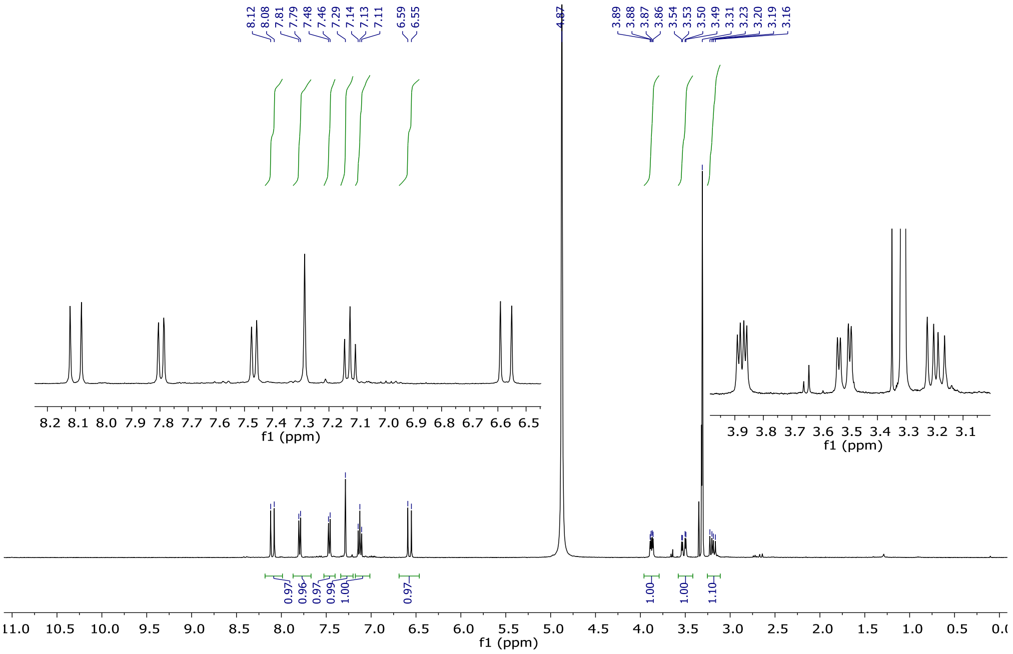

^{13}C NMR (101 MHz, MeOD)

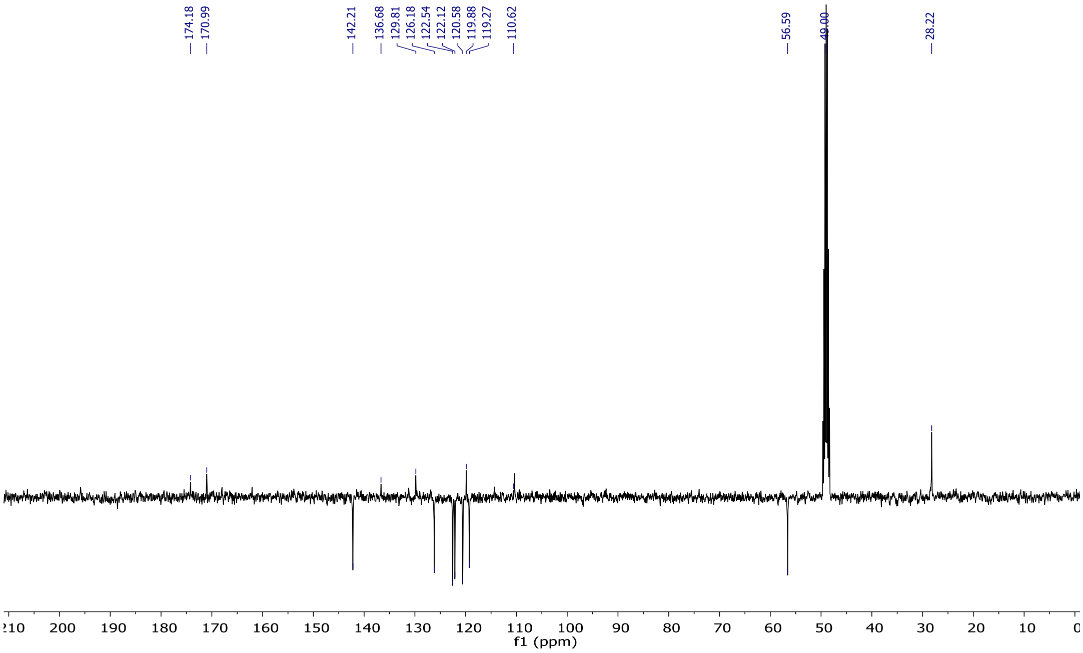

(*S*)-3-(4-bromo-1H-indol-3-yl)-2-((*tert*-butoxycarbonyl)amino)propanoic acid

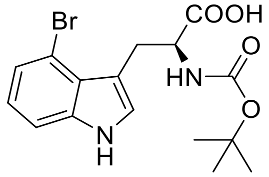

^{1}H NMR (500 MHz, MeOD)

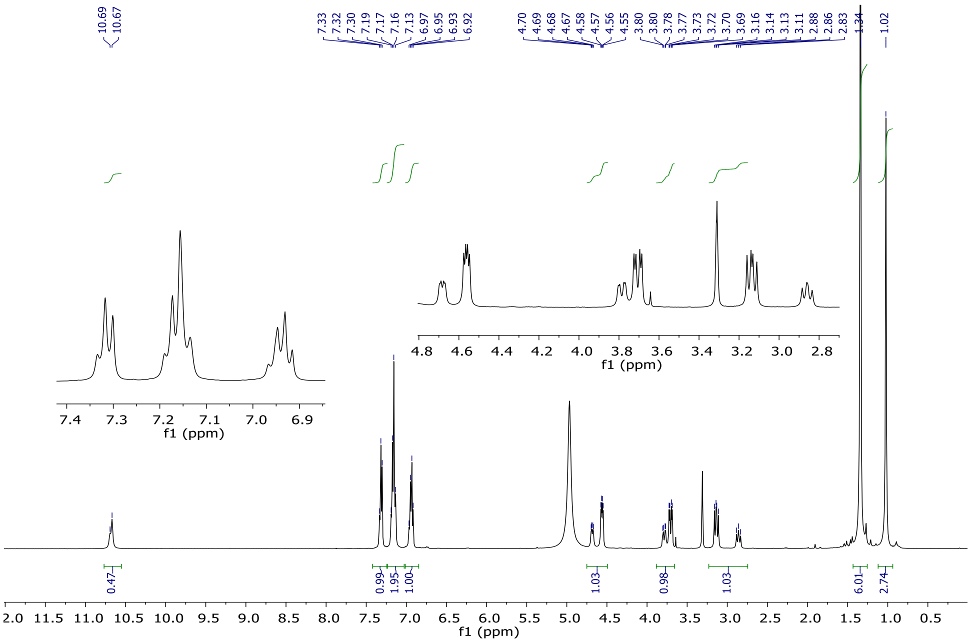

^{13}C NMR (126 MHz, MeOD)

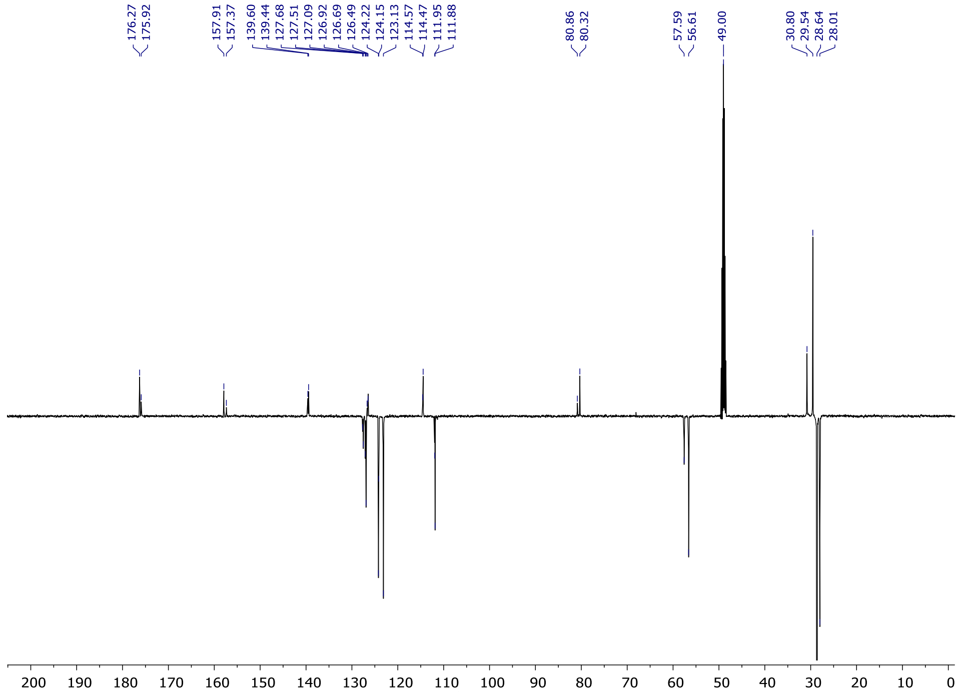

[1]D gs NOESY – 4.576 (500 MHz, MeOD)

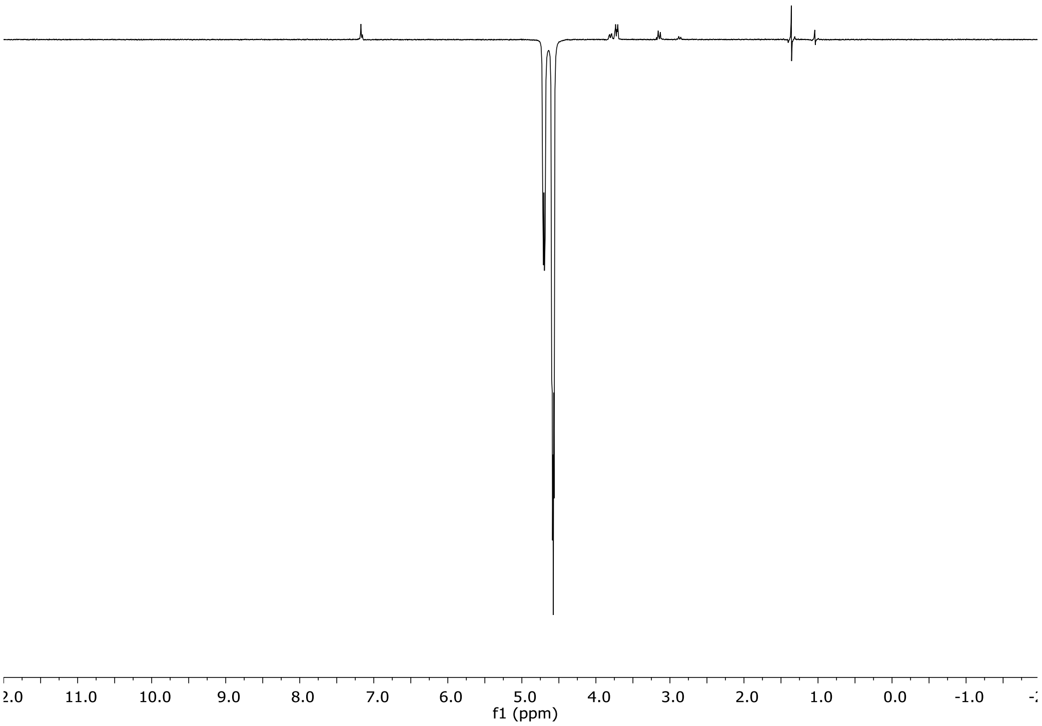

[1]D gs NOESY – 4.695 (500 MHz, MeOD)

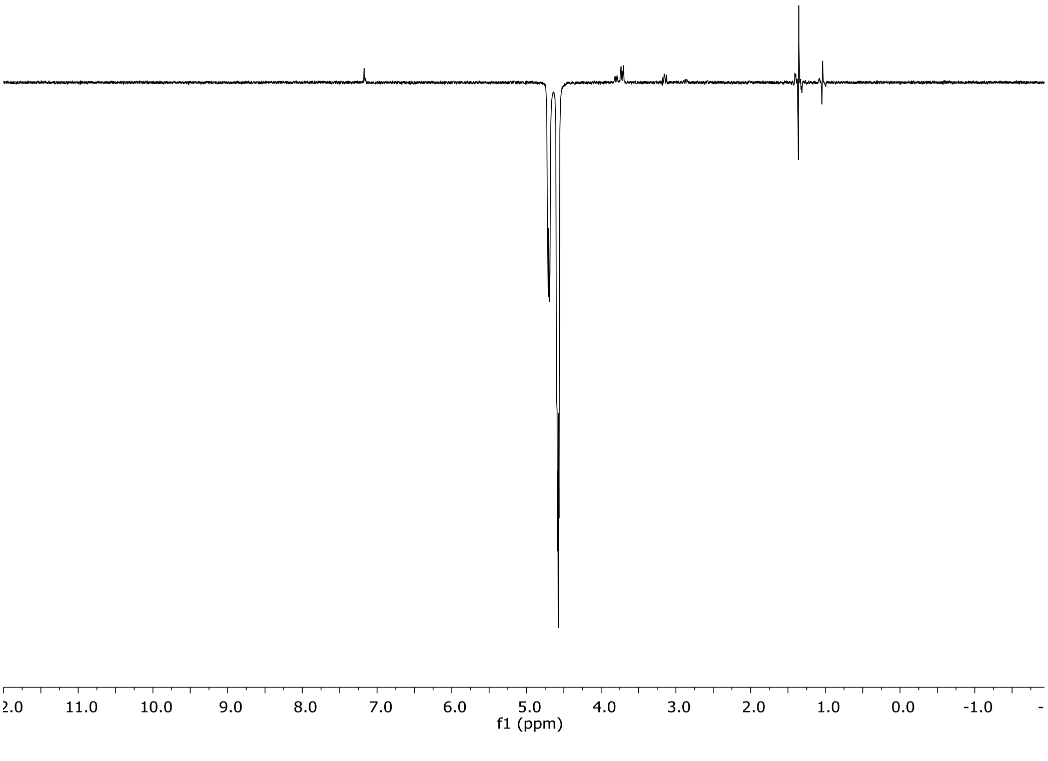

(*E*)-3-(3-((*S*)-2-((*tert*-butoxycarbonyl)amino)-2-carboxyethyl)-1H-indol-4-yl)acrylic acid

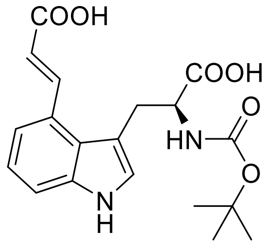

^{1}H NMR (500 MHz, MeOD)

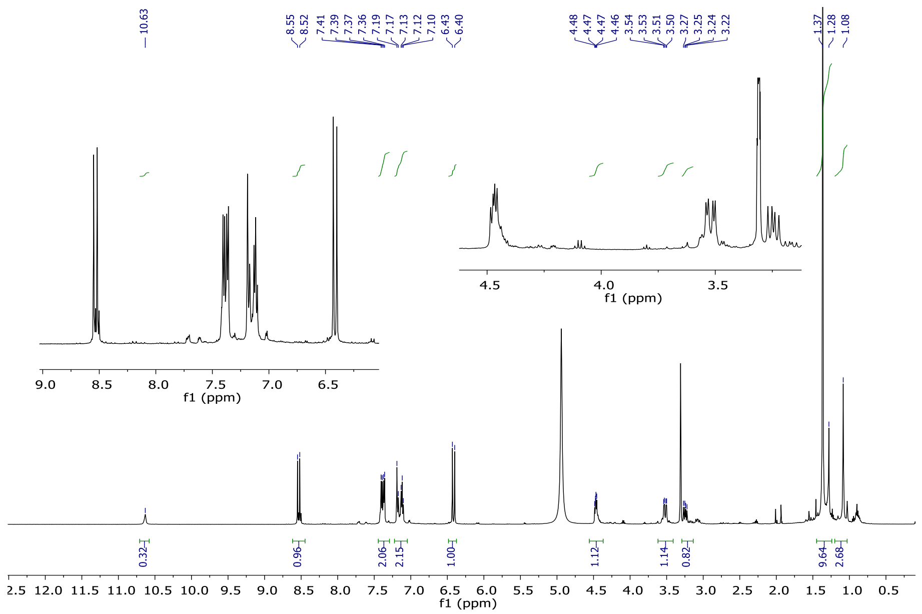

^{13}C NMR (126 MHz, MeOD)

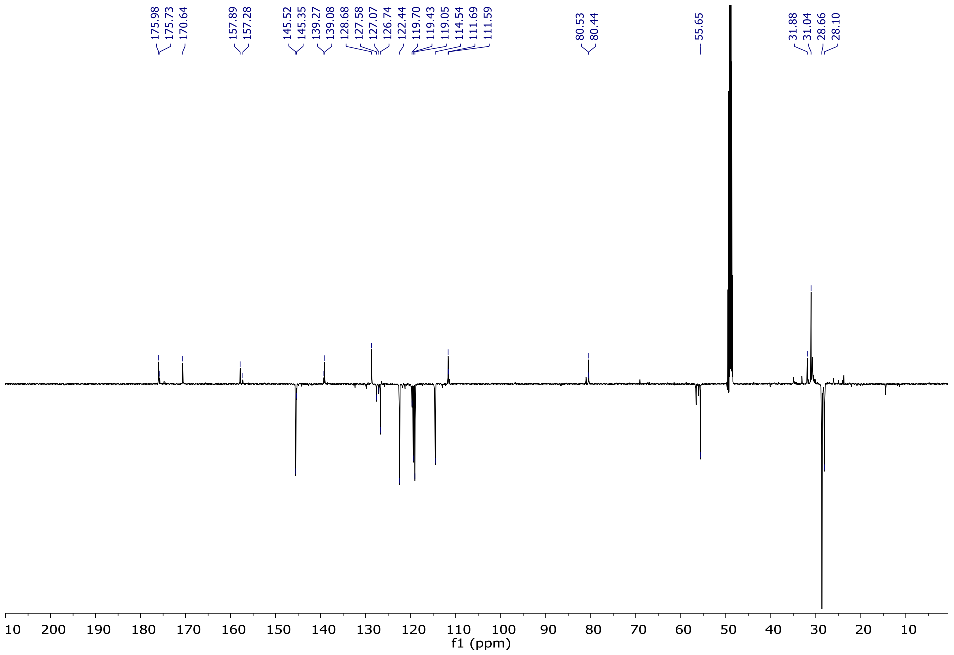

^{1}D gs NOESY – 1.385 (500 MHz, MeOD)

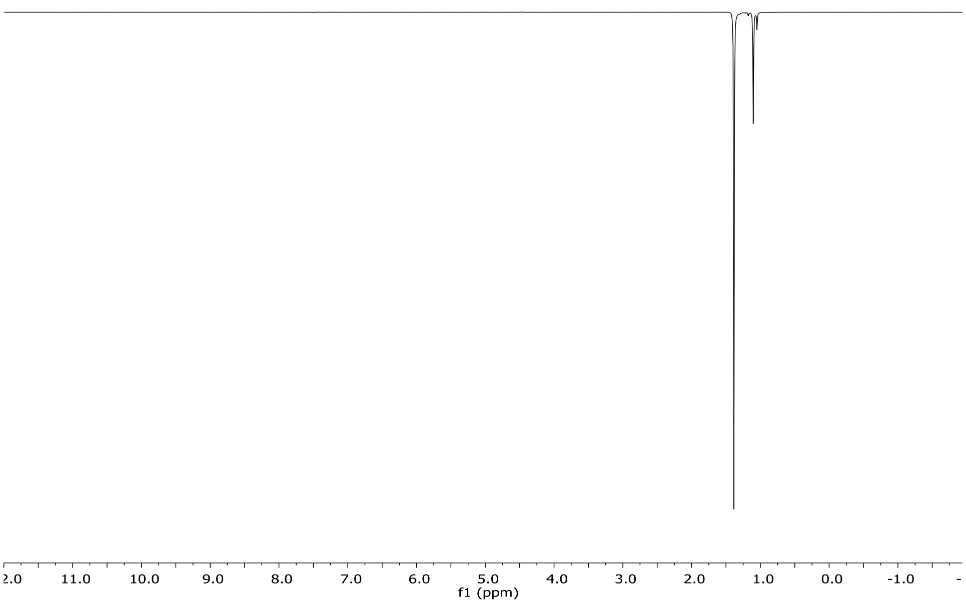

^{1}D gs NOESY – 1.104 (500 MHz, MeOD)

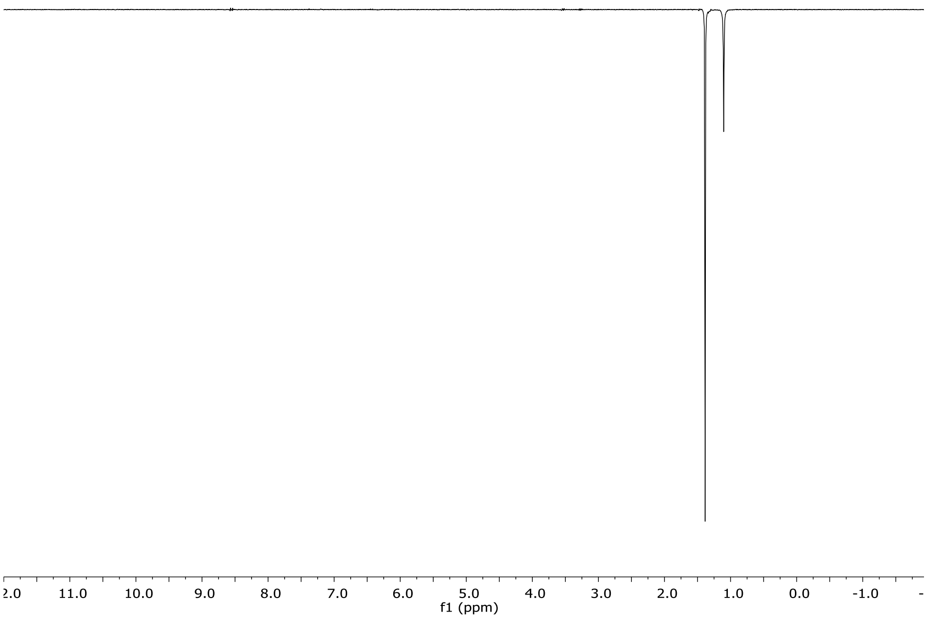

(*E*)-3-(3-((*S*)-2-amino-2-carboxyethyl)-1H-indol-4-yl)acrylic acid

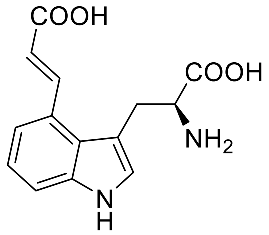

^{1}H NMR (500 MHz, MeOD)

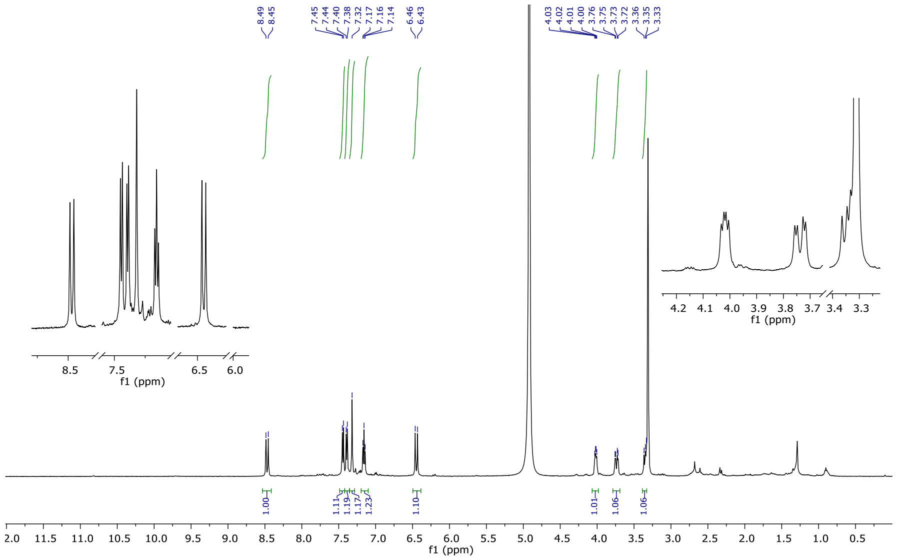

^{13}C NMR (126 MHz, MeOD)

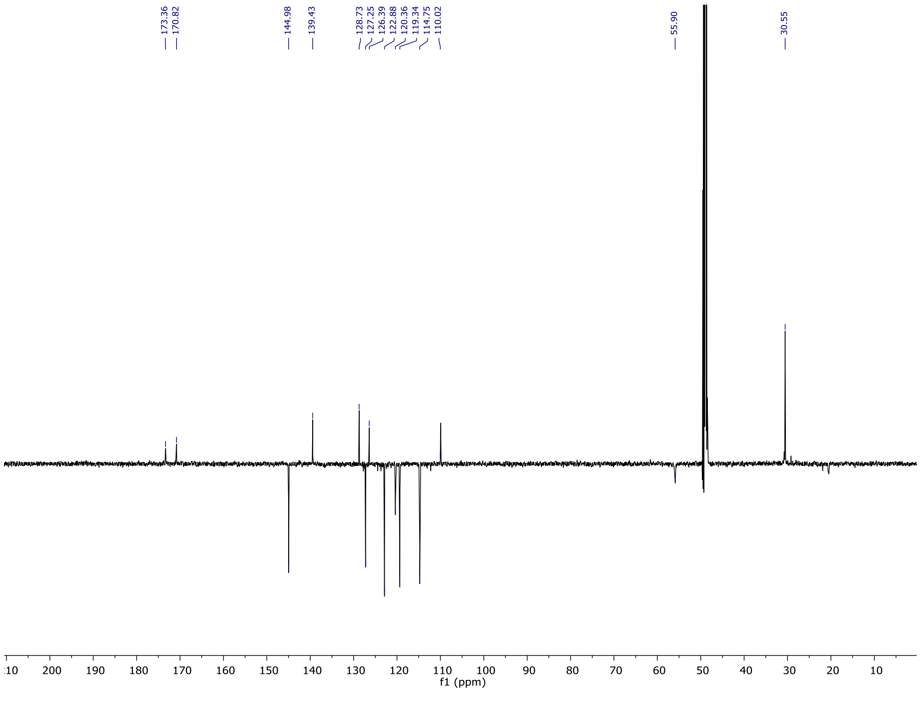

(*E*)-3-(4-((*S*)-2-((*tert*-butoxycarbonyl)amino)-2-carboxyethyl)phenyl)acrylic acid

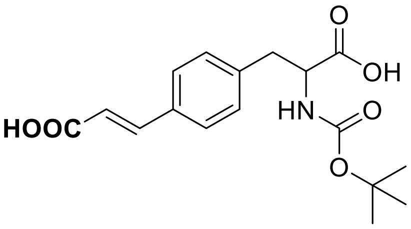

^{1}H NMR (500 MHz, MeOD)

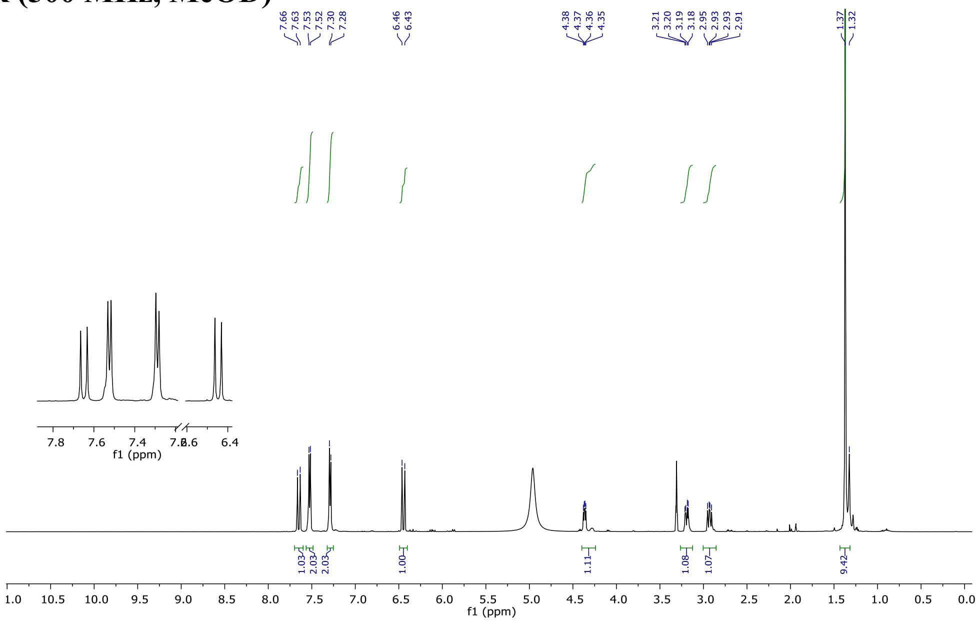

^{13}C NMR (126 MHz, MeOD)

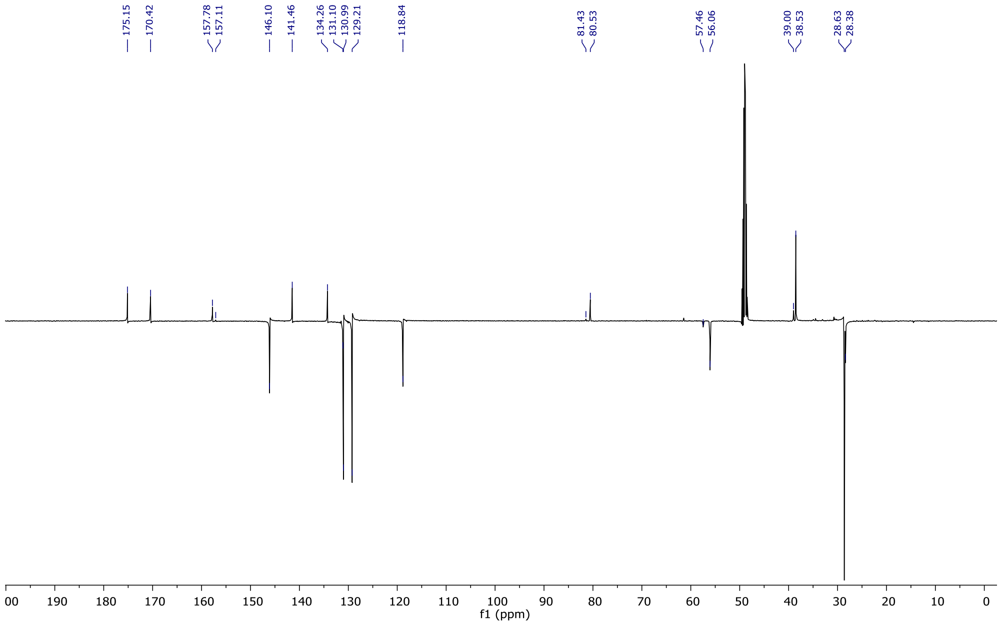

^{1}D gs NOESY – 1.37 (500 MHz, MeOD)

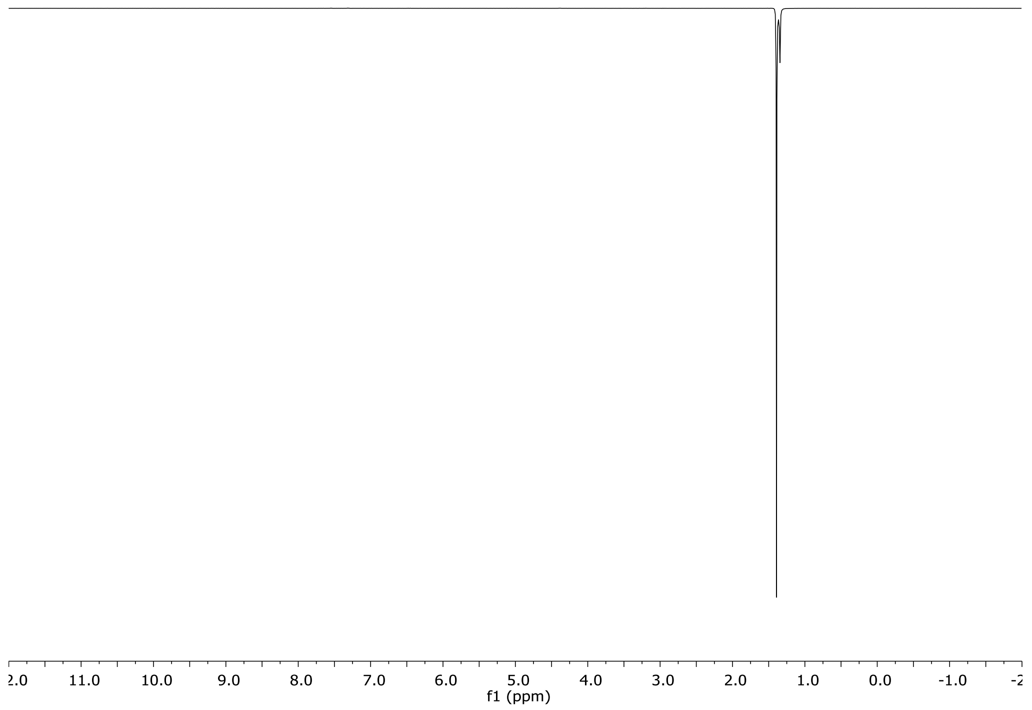

^{1}D gs NOESY – 1.32 (500 MHz, MeOD)

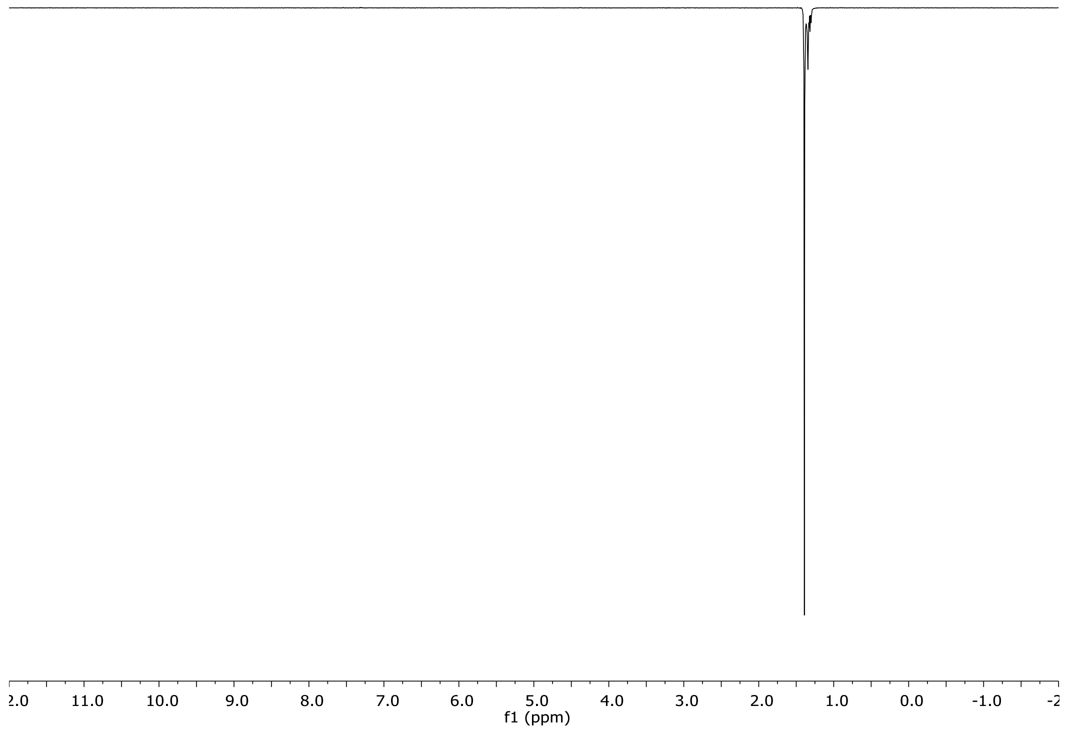

(*E*)-3-(2-(2-((*tert*-butoxycarbonyl)amino)-2-carboxyethyl)phenyl)acrylic acid

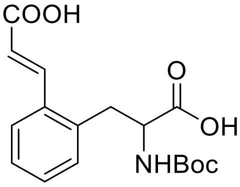

^{1}H NMR (500 MHz, MeOD)

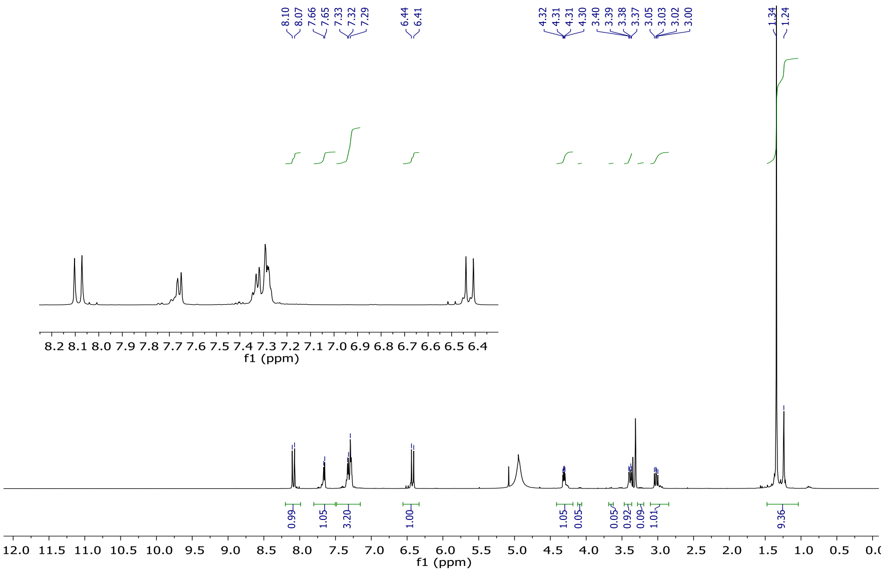

^{13}C NMR (126 MHz, MeOD)

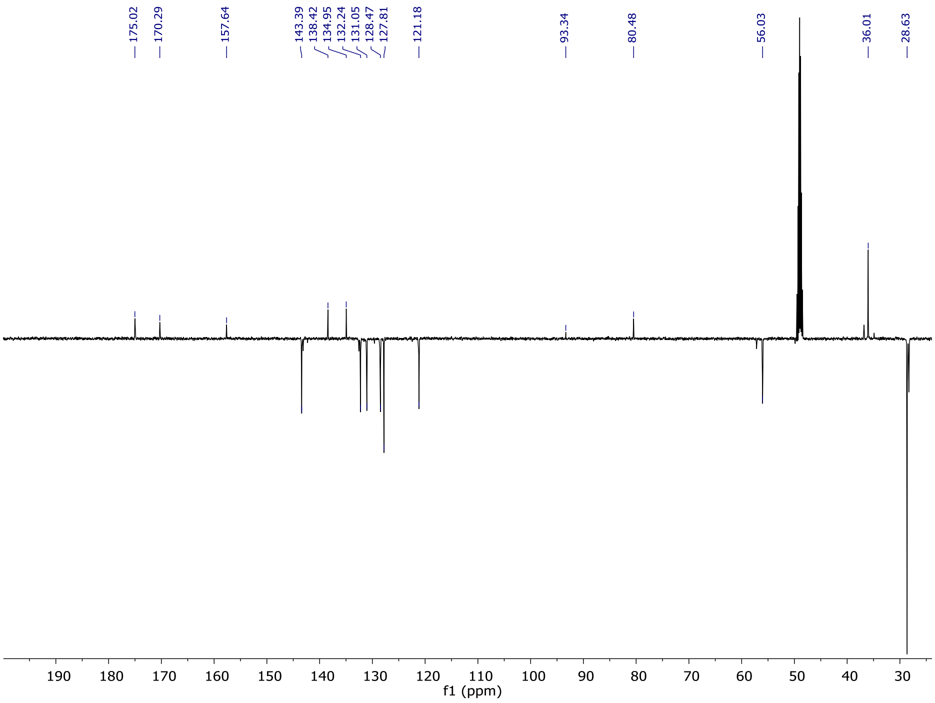

[1]D gs NOESY – 1.340 (500 MHz, MeOD)

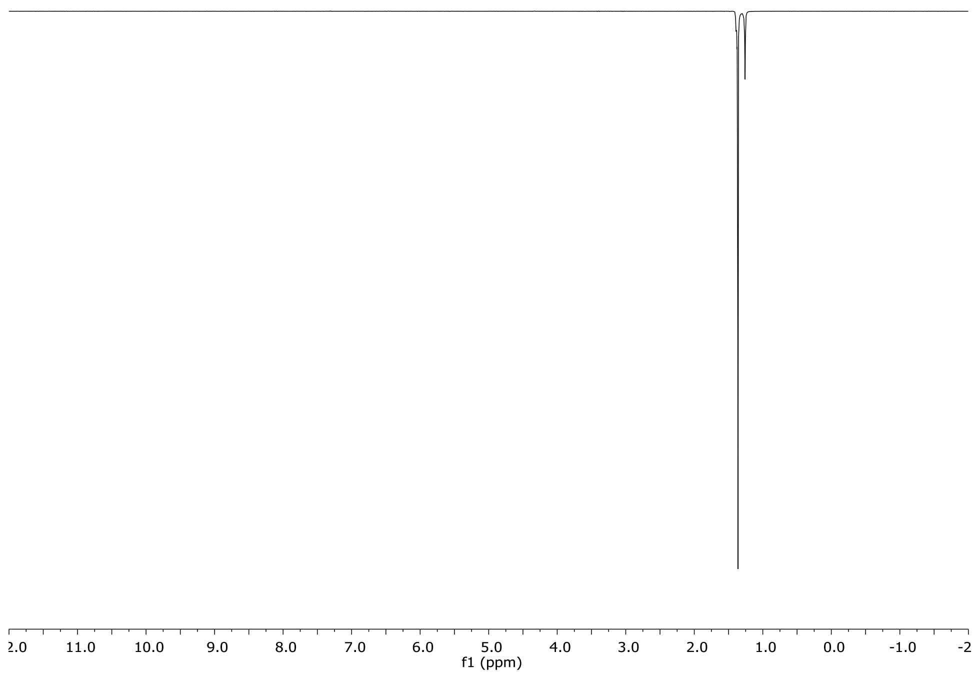

[1]D gs NOESY – 1.240 (500 MHz, MeOD)

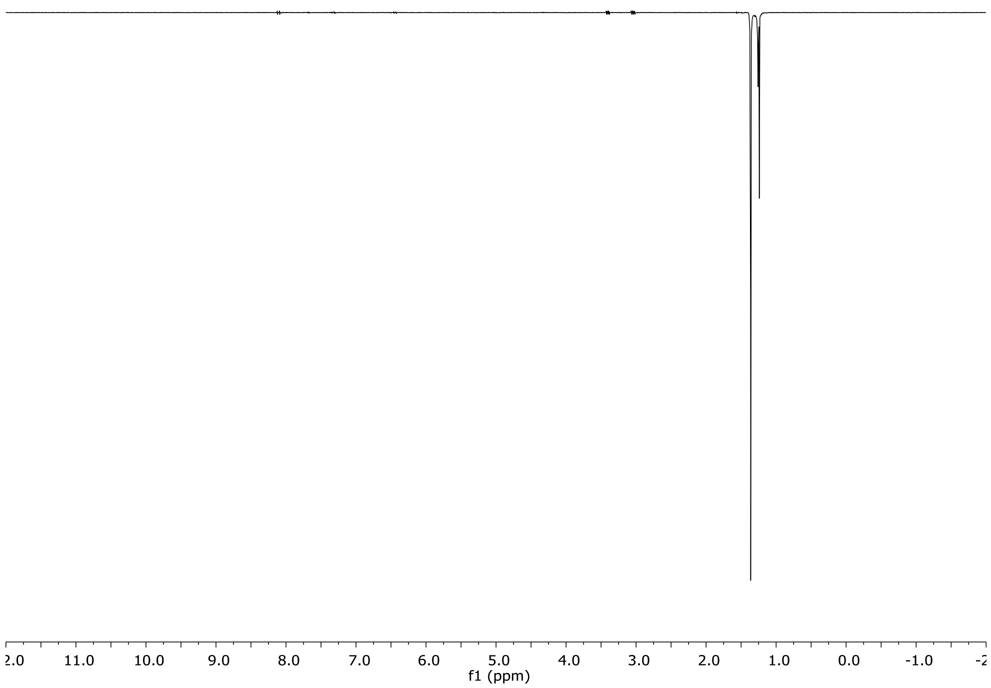

(*S*)-2-amino-3-(5-styryl-1H-indol-3-yl)propanoic acid

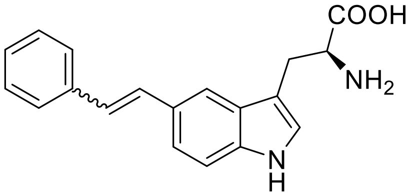

^{1}H NMR (500 MHz, MeOD)

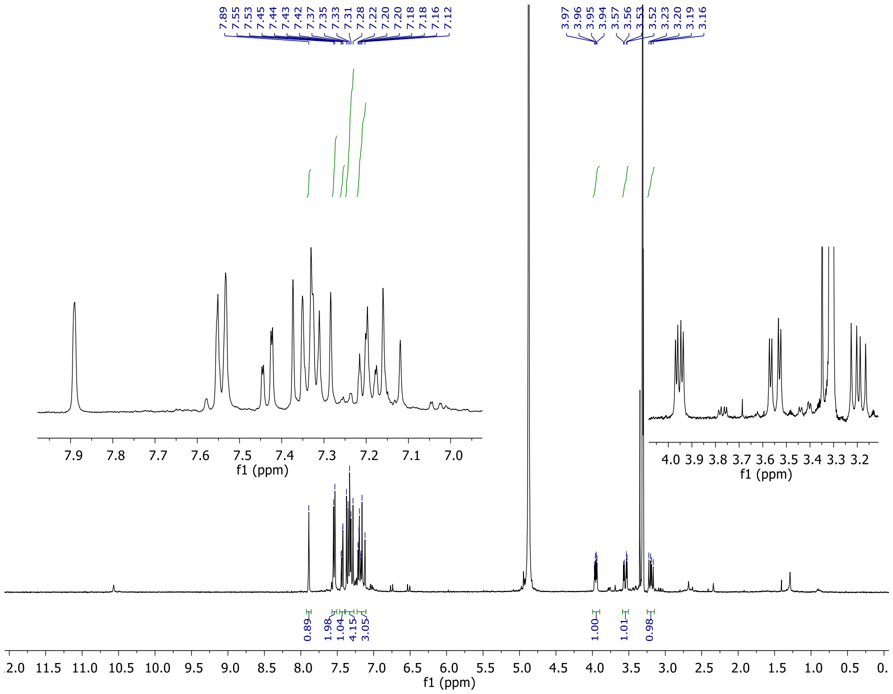

^{13}C NMR (126 MHz, MeOD)

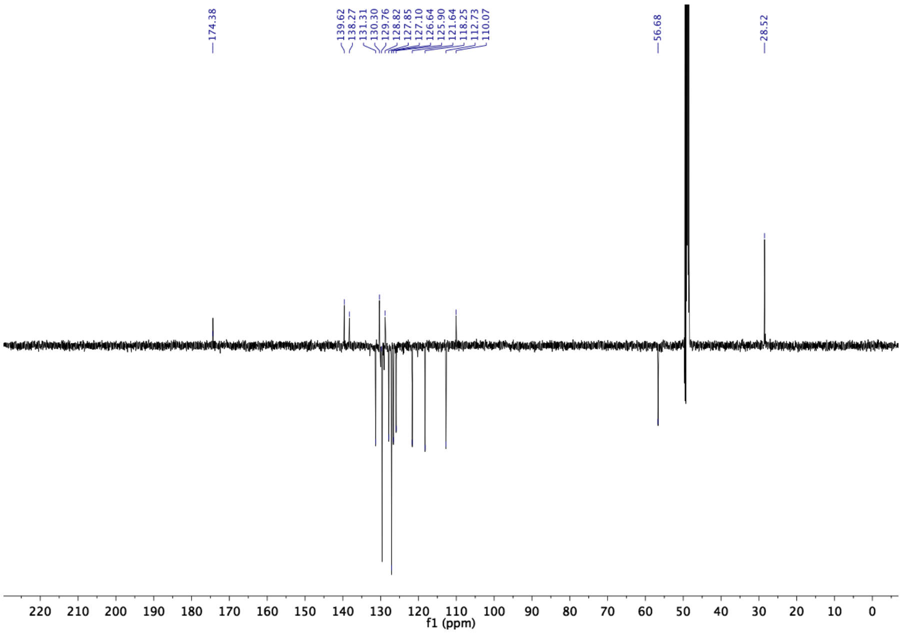

(*S*)-2-amino-3-(7-(*E*)-styryl-1H-indol-3-yl)propanoic acid

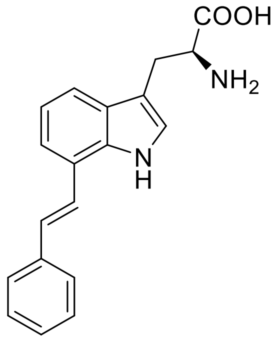

^{1}H NMR (400 MHz, MeOD)

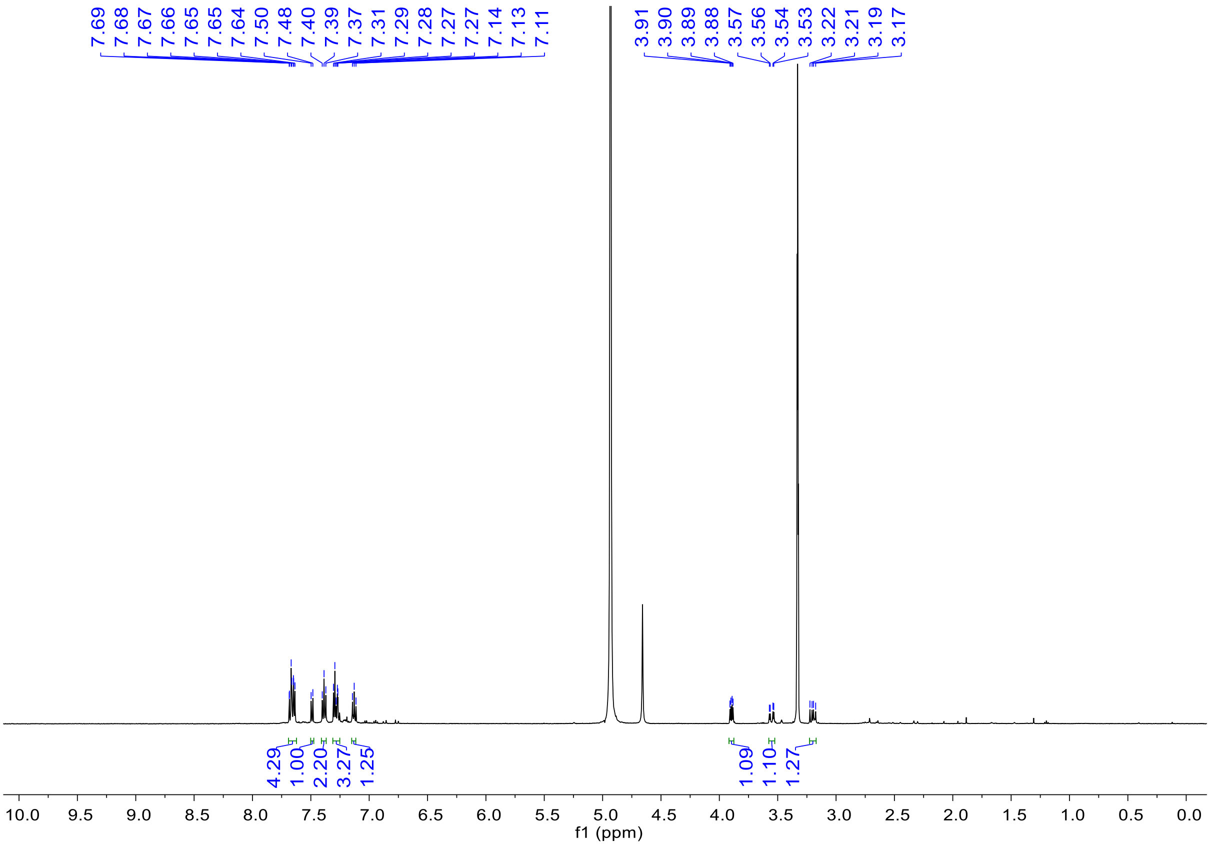

^{13}C NMR (126 MHz, MeOD)

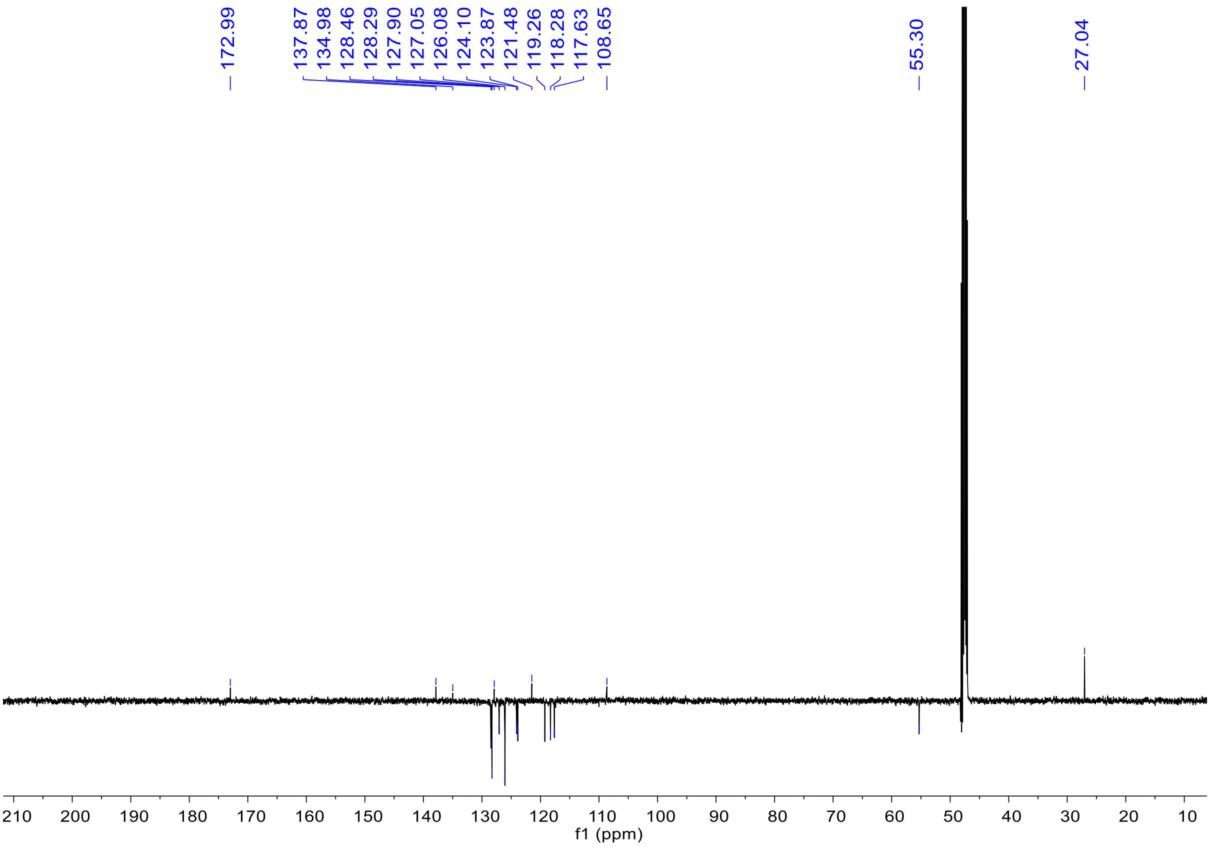

COSY NMR (500 MHz, MeOD)

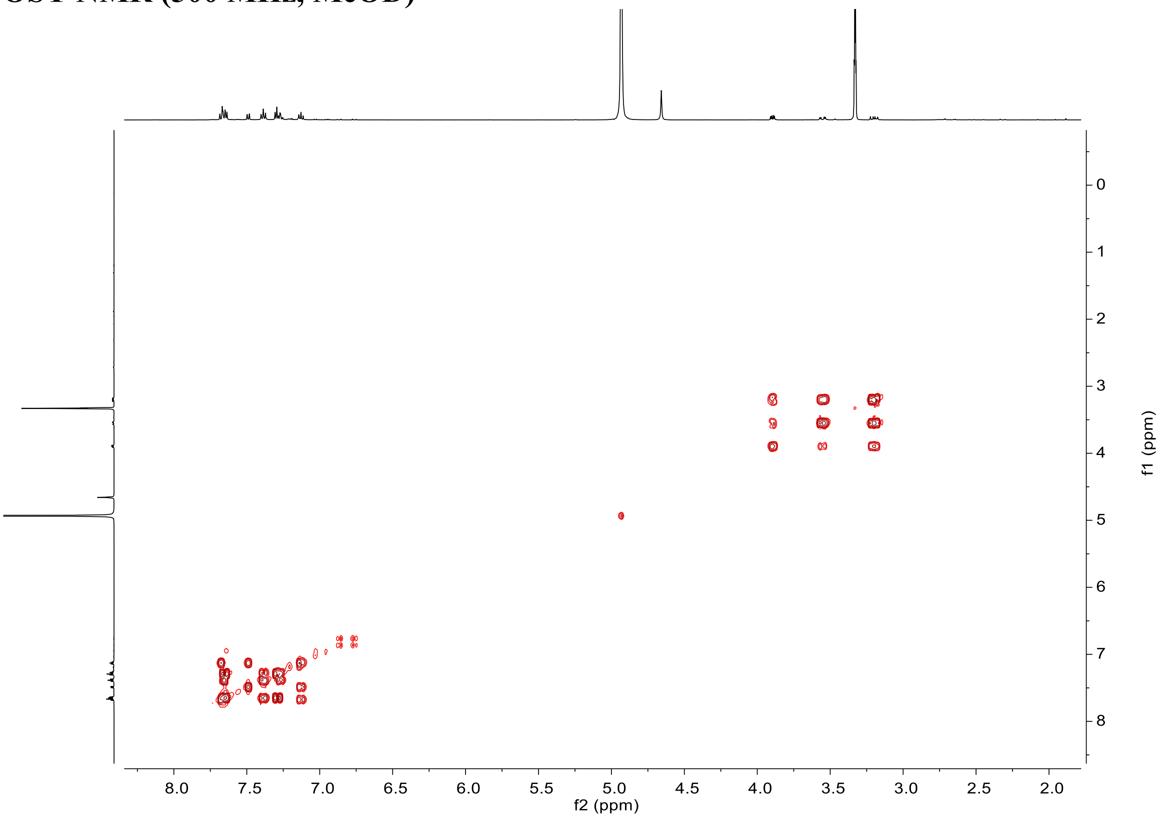

HSQC NMR (500 MHz, MeOD)

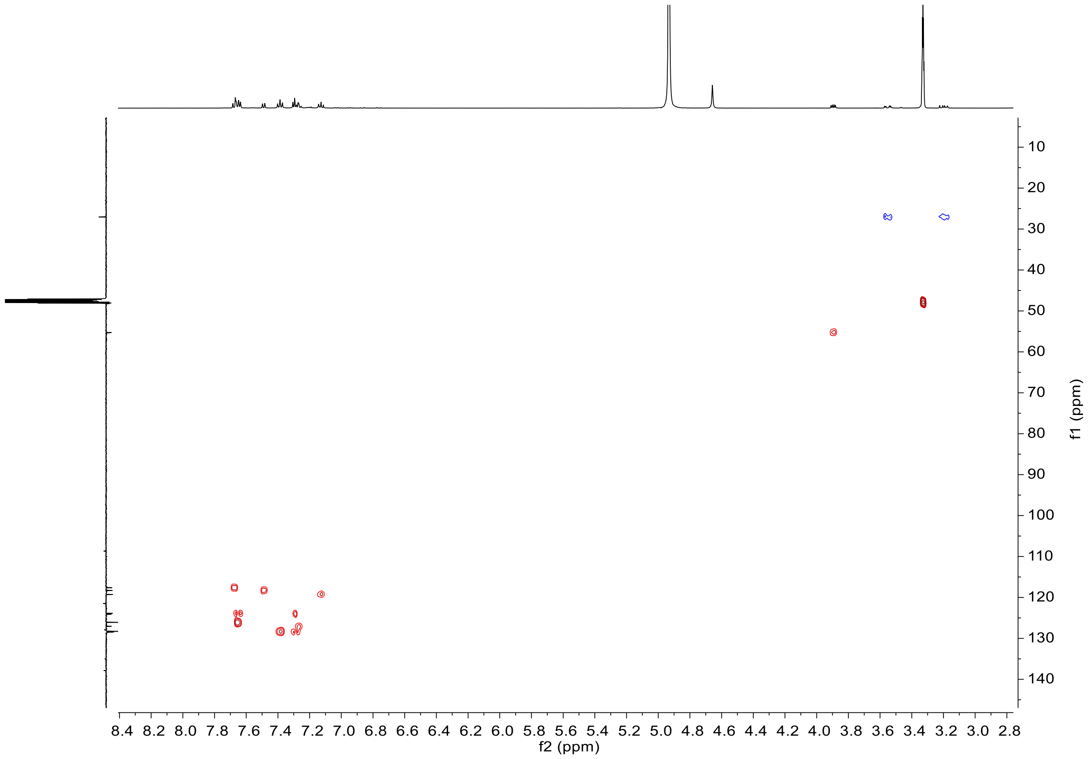

HMBC NMR (500 MHz, MeOD)

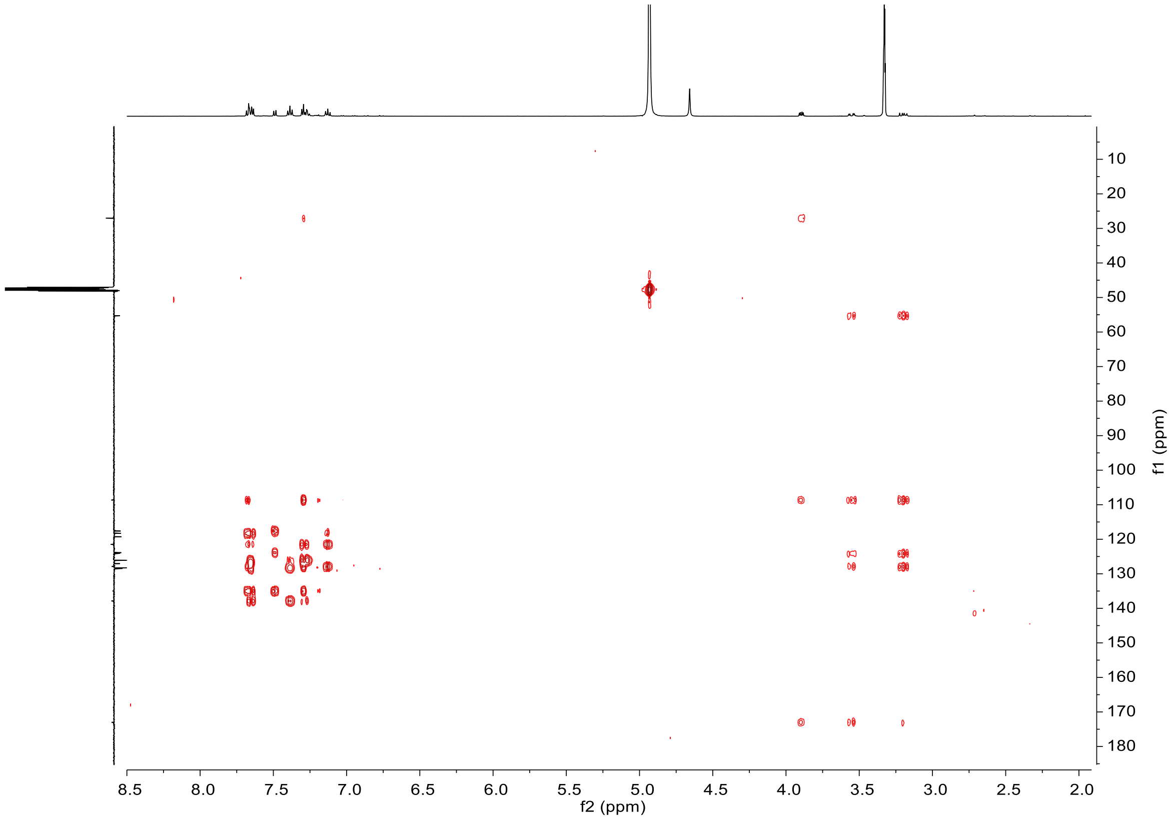

(*S*)-2-amino-3-(5-(4-methylstyryl)-1H-indol-3-yl)propanoic acid

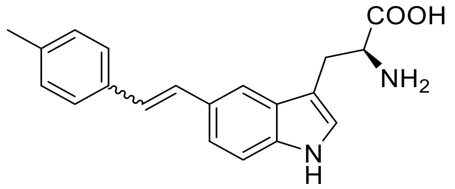

^{1}H NMR (500 MHz, MeOD)

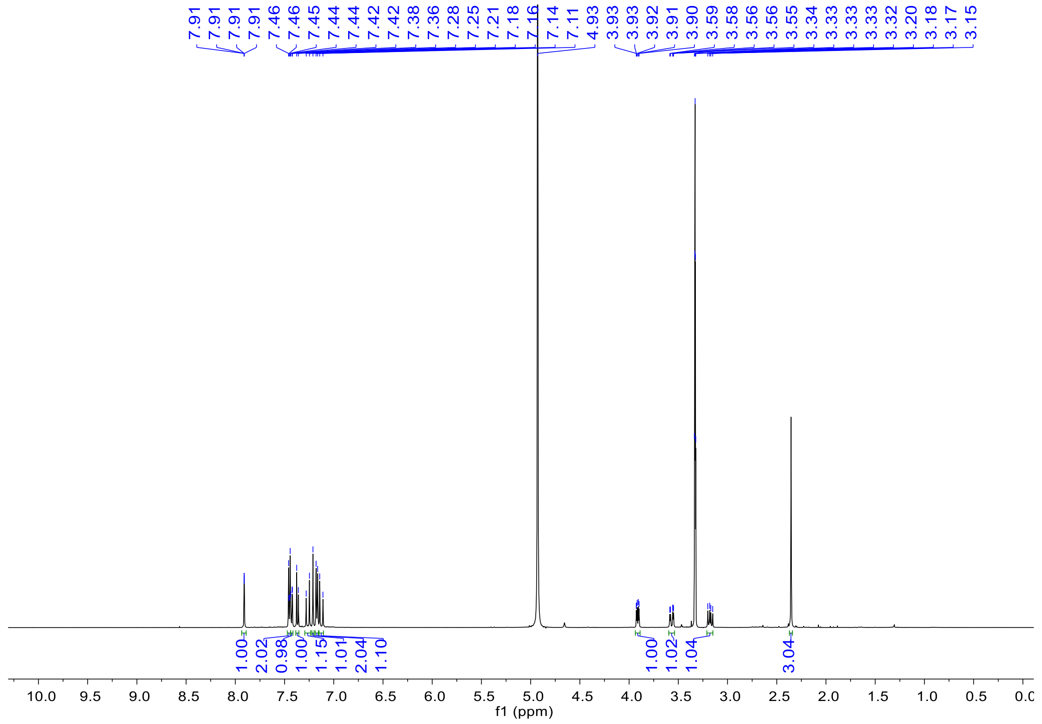

^{13}C NMR (126 MHz, MeOD)

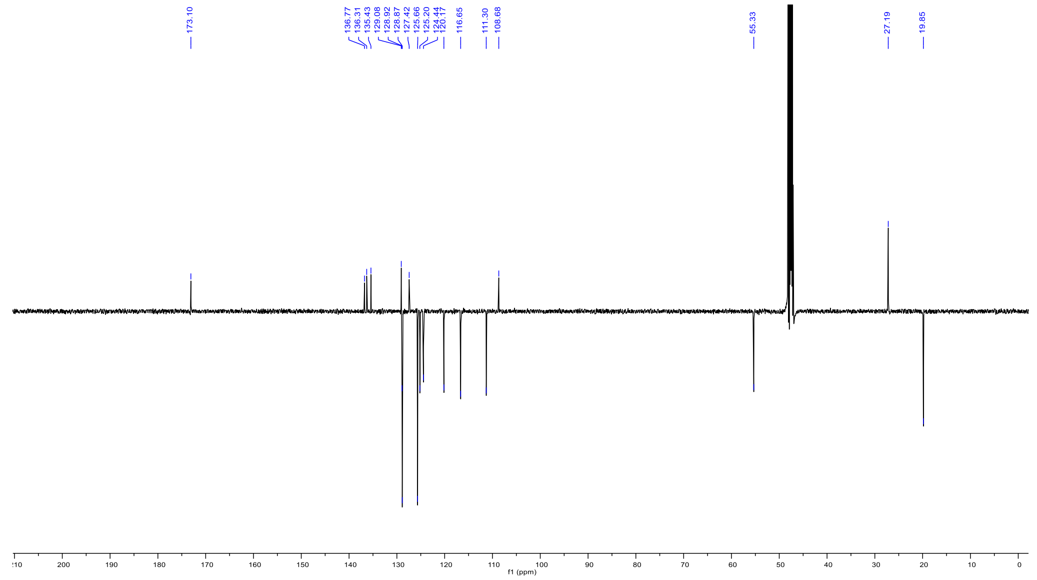

COSY NMR (500 MHz, MeOD)

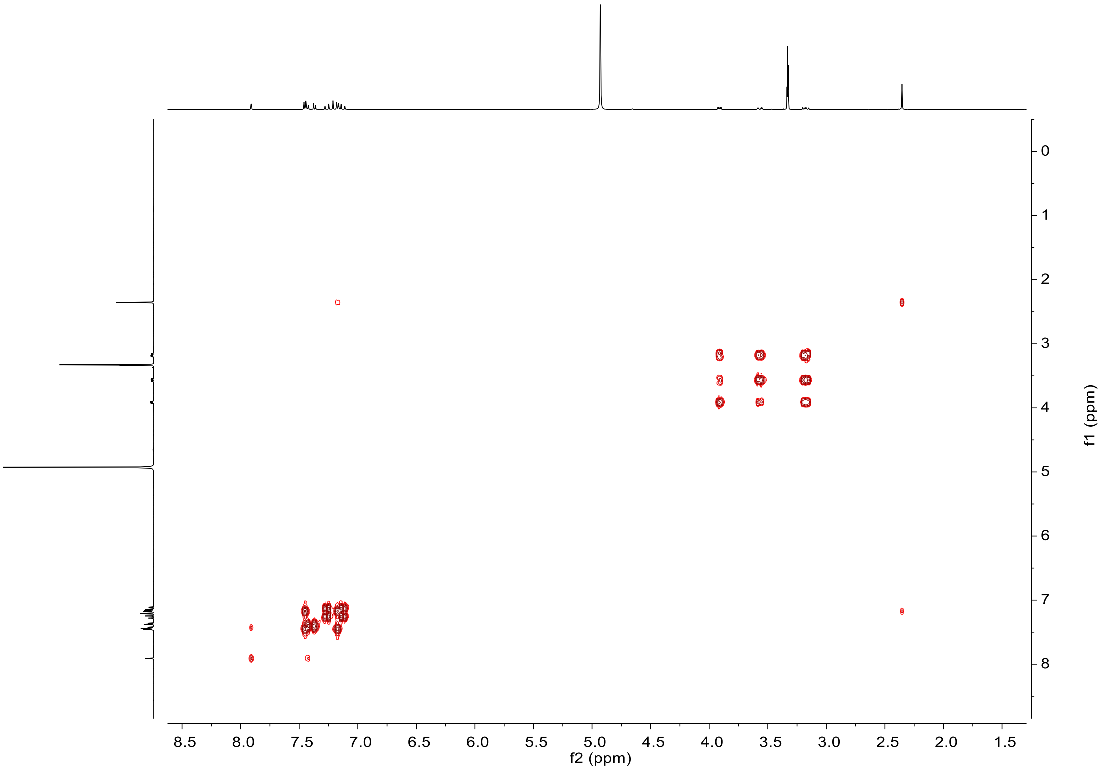

HSQC NMR (500 MHz, MeOD)

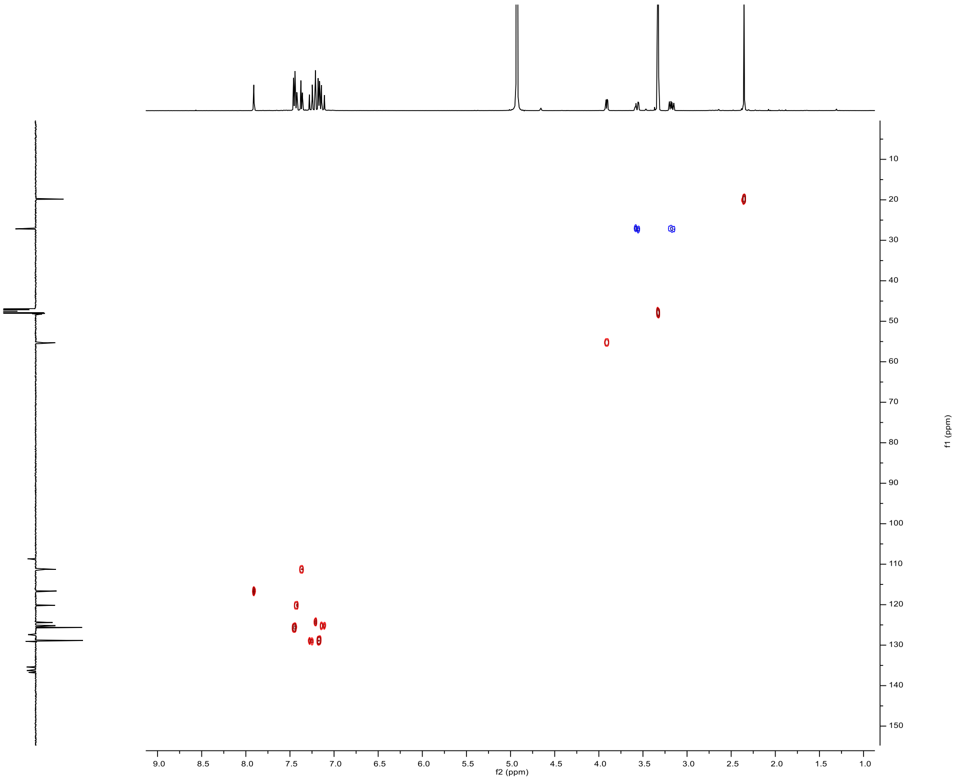

HMBC NMR (500 MHz, MeOD)

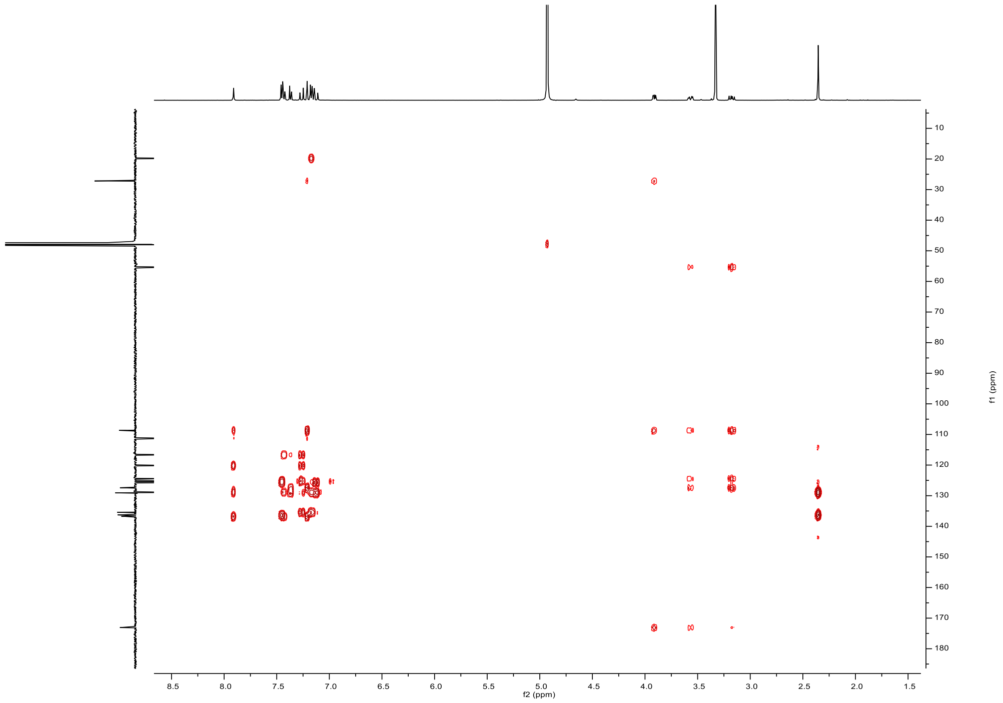

(*S*)-2-amino-3-(5-(4-aminostyryl)-1H-indol-3-yl)propanoic acid

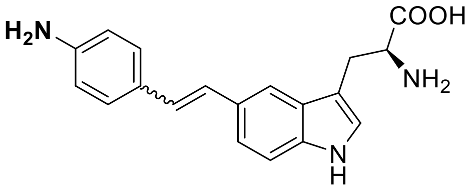

^{1}H NMR (500 MHz, MeOD)

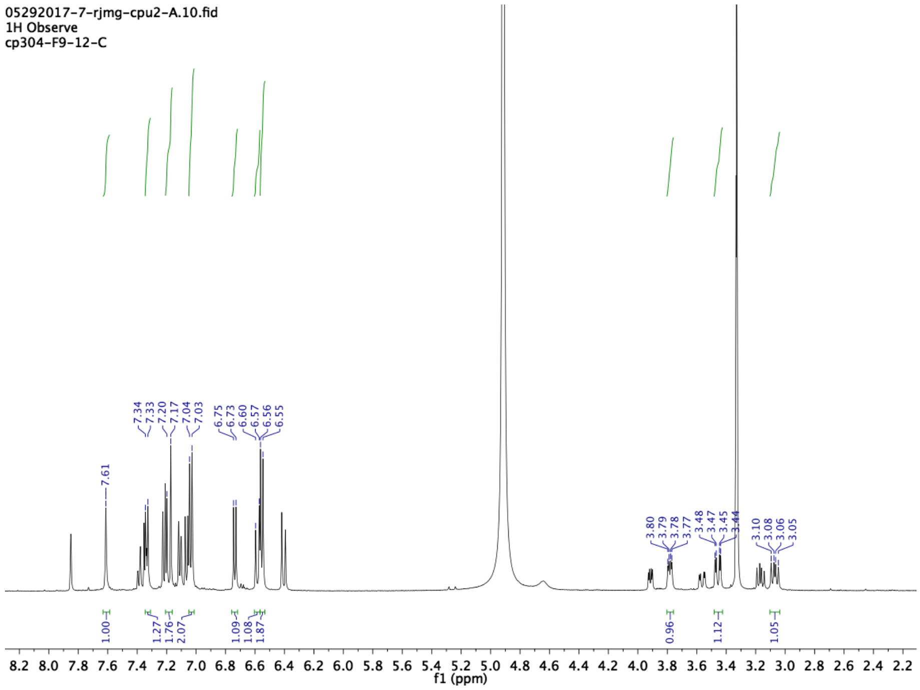

^{13}C NMR (126 MHz, MeOD)

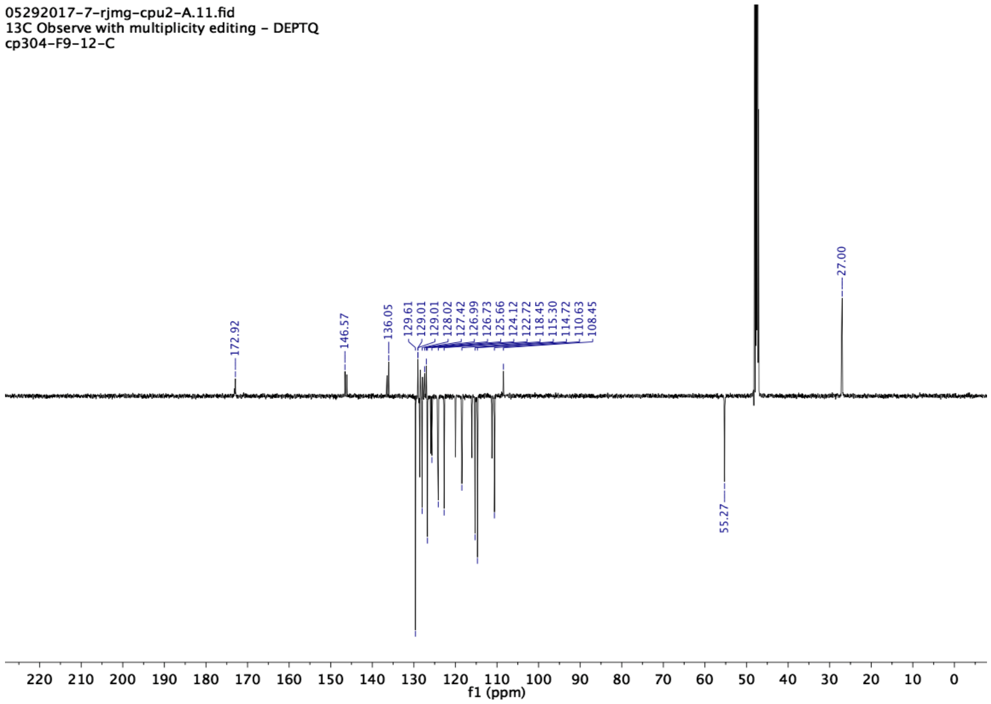

(*S*)-2-amino-3-(7-(4-aminostyryl)-1H-indol-3-yl)propanoic acid

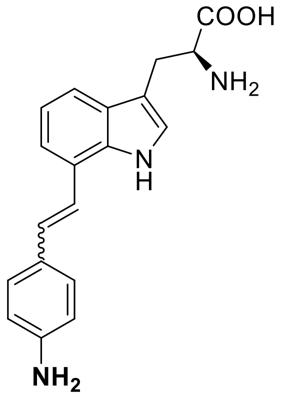

^{1}H NMR (500 MHz, MeOD)

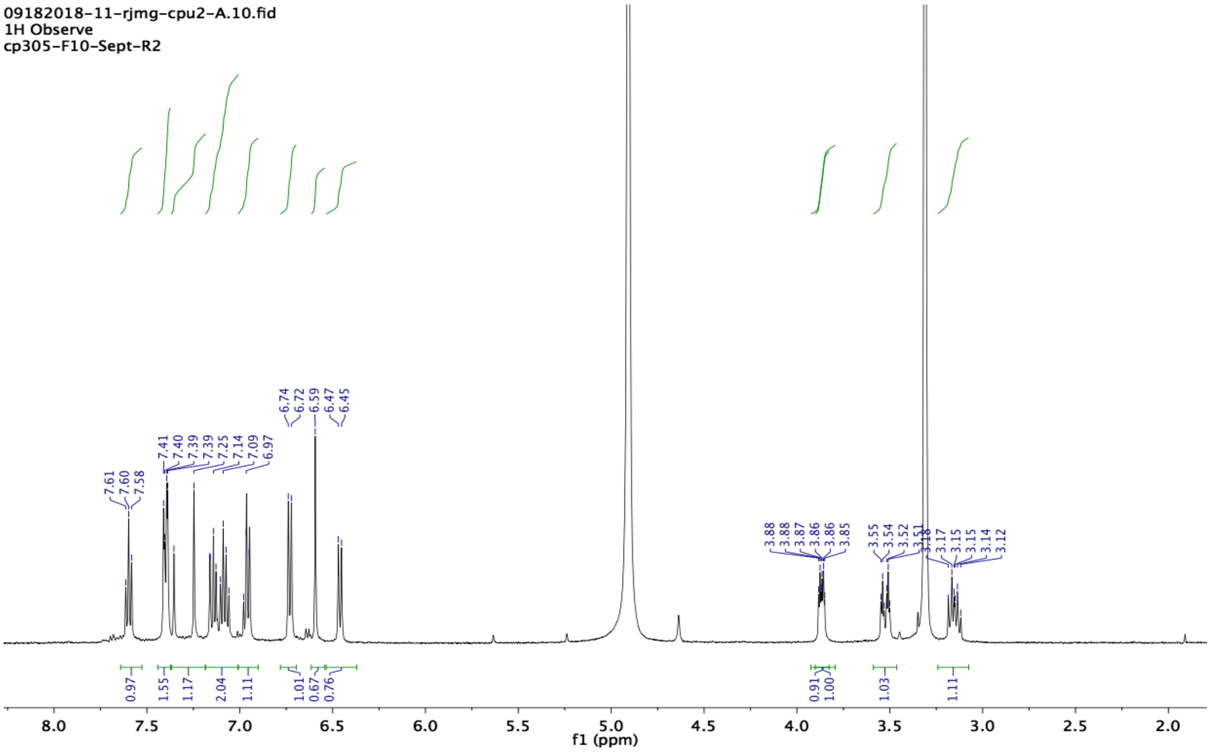

^{13}C NMR (126 MHz, MeOD)

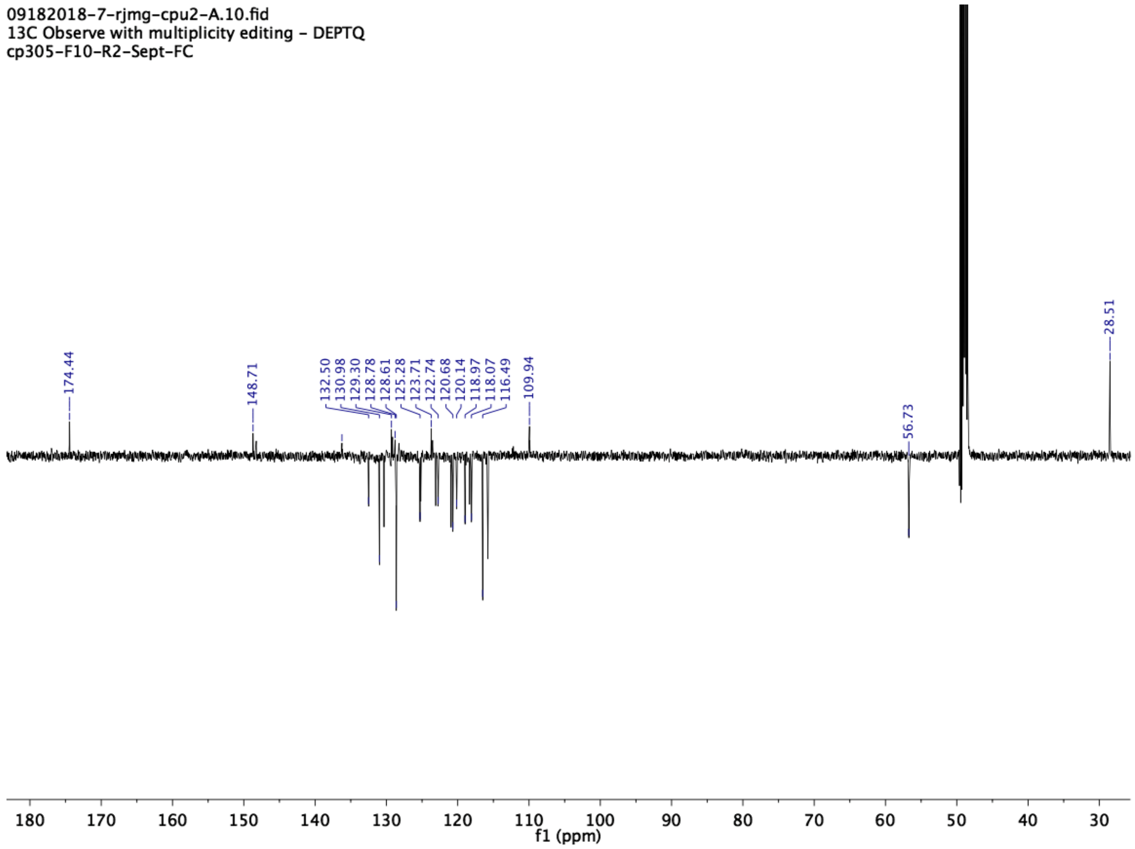

(*S*)-2-amino-3-(5-(4-fluorostyryl)-1H-indol-3-yl)propanoic acid

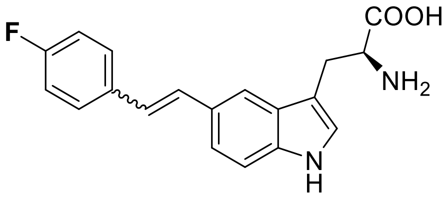

^{1}H NMR (500 MHz, MeOD)

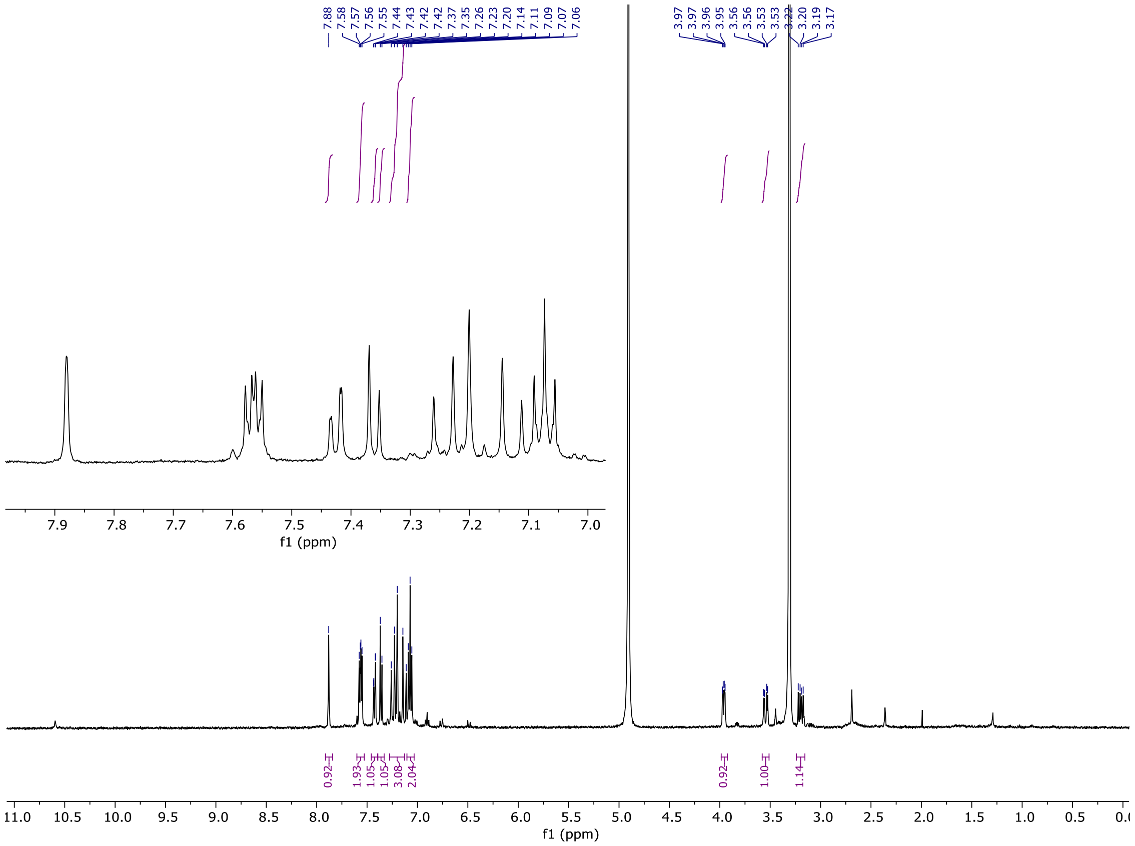

^{13}C NMR (126 MHz, DMSO-d_6)

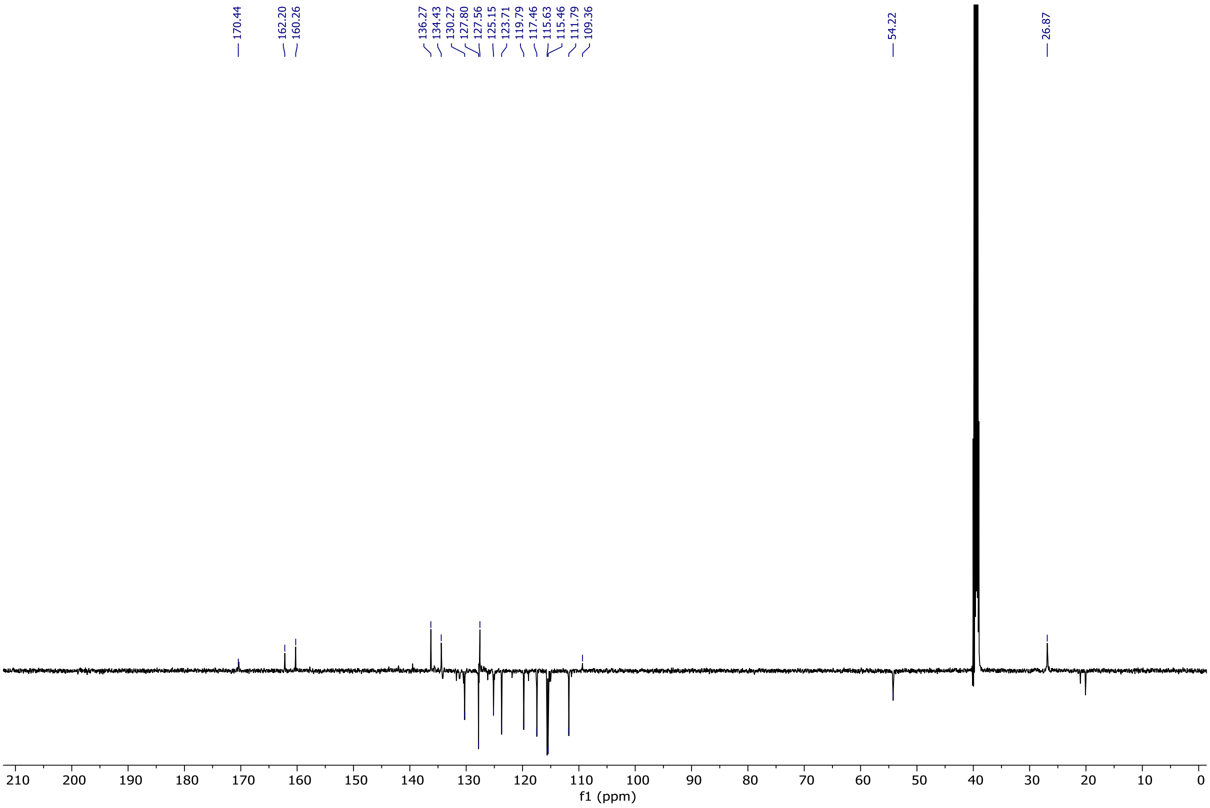

^{19}F NMR (471 MHz, DMSO-d_6)

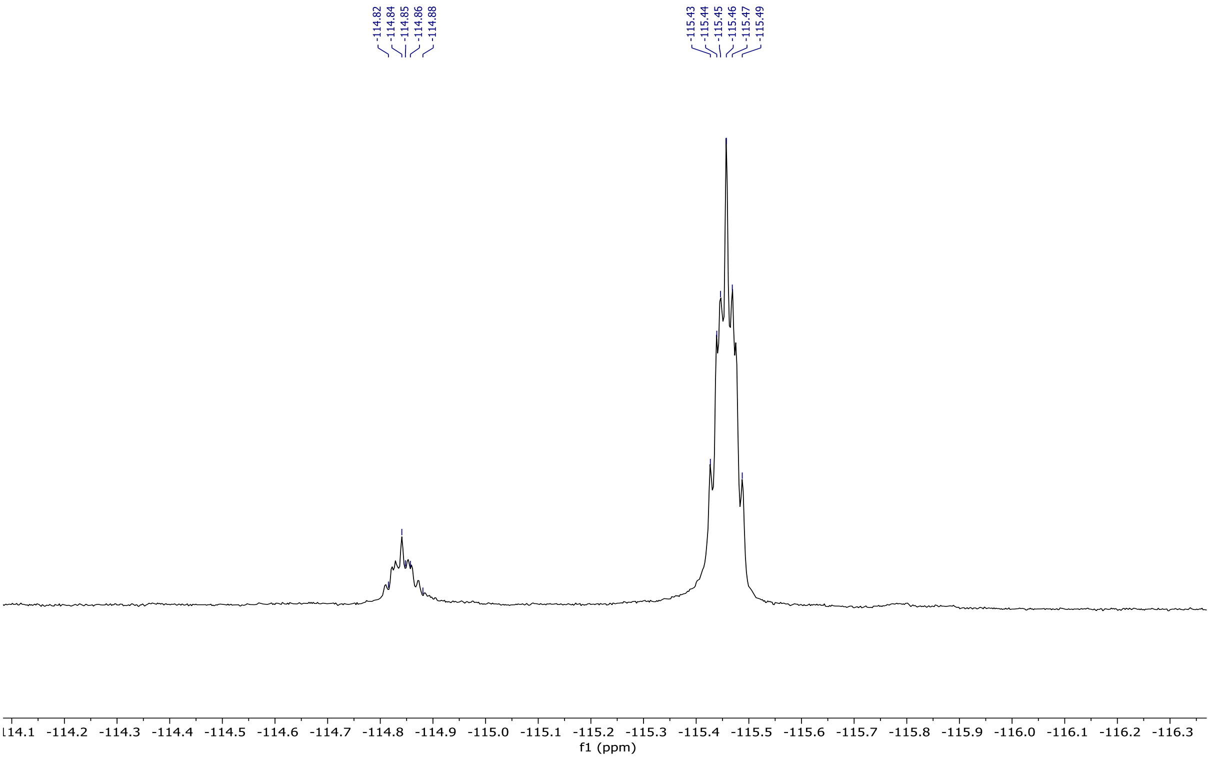

(*S*)-2-amino-3-(7-((*E*)-4-fluorostyryl)-1H-indol-3-yl)propanoic acid

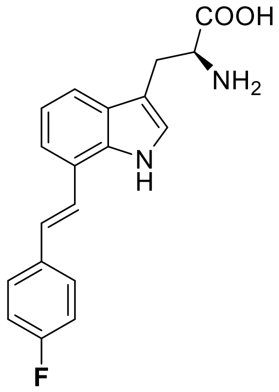

^{1}H NMR (500 MHz, MeOD)

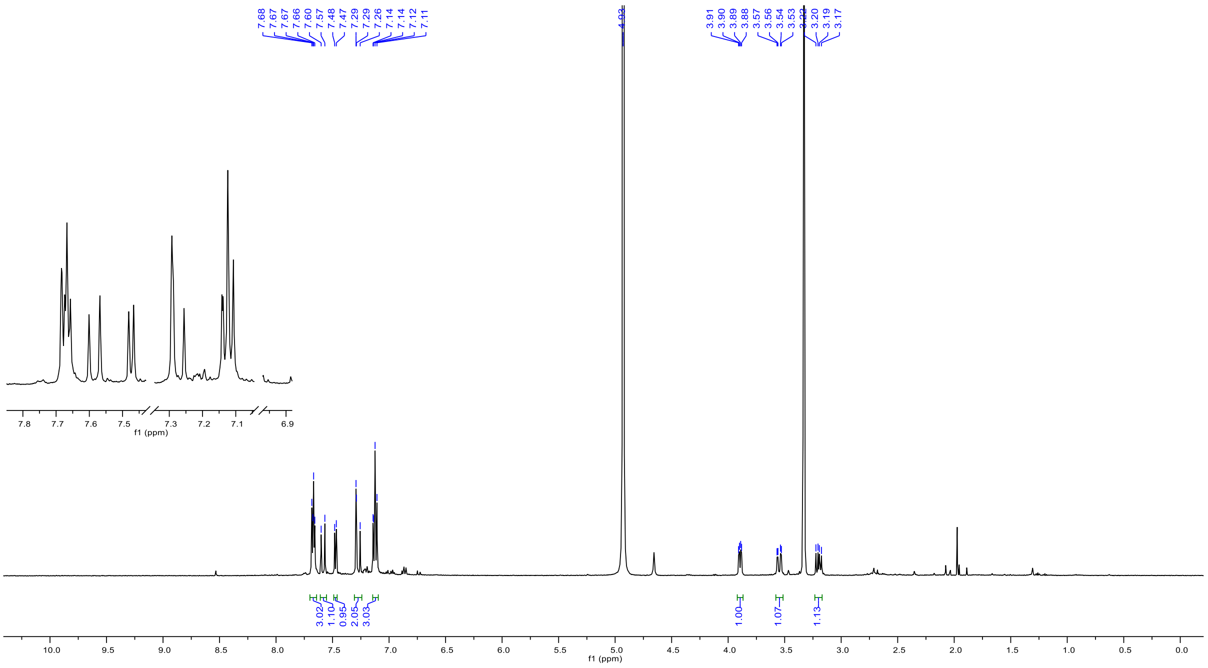

^{13}C NMR (126 MHz, MeOD)

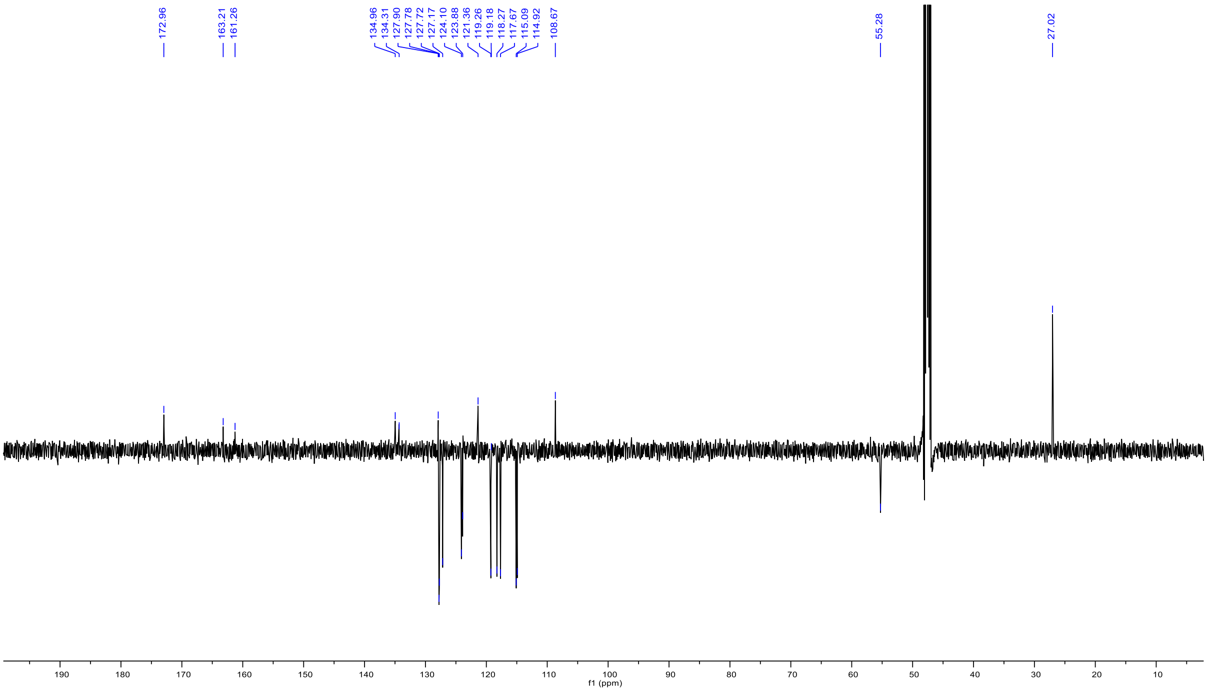

^{19}F NMR (471 MHz, MeOD)

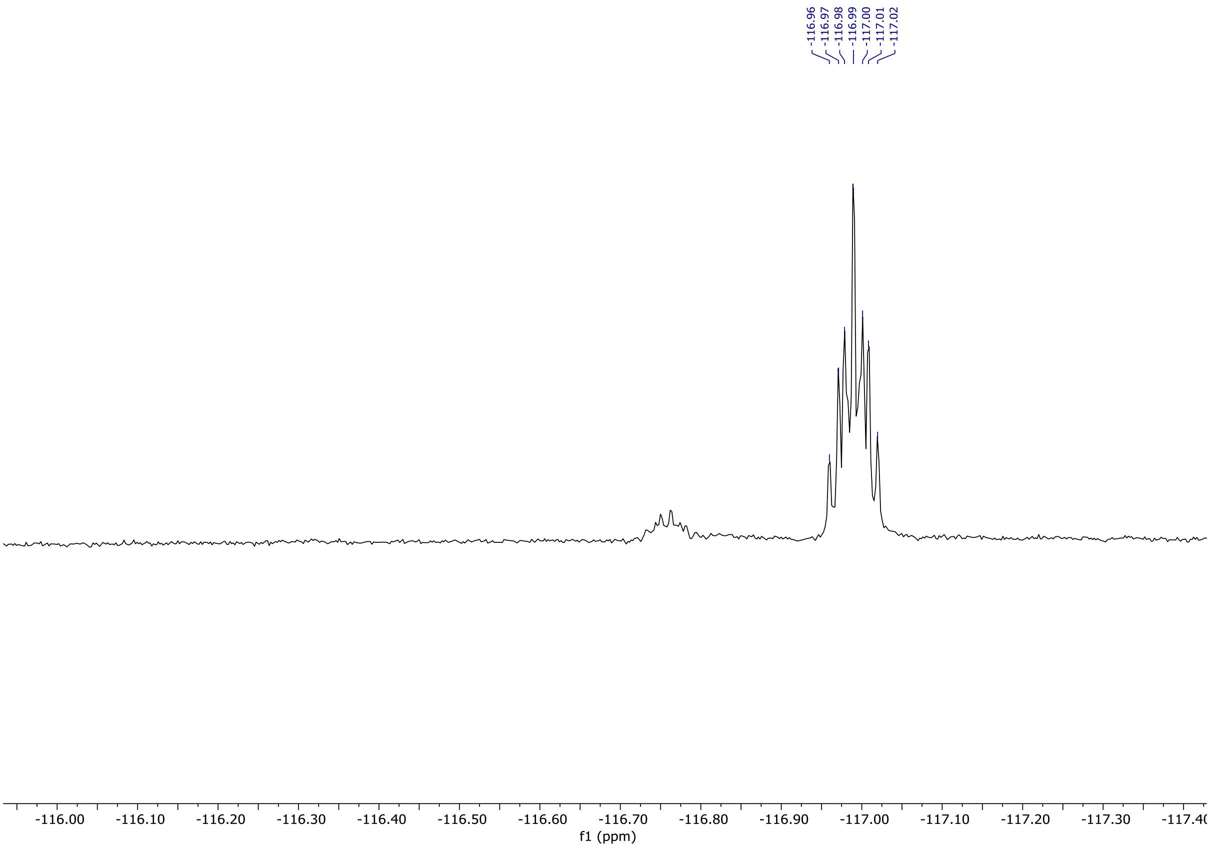

COSY NMR (500 MHz, MeOD)

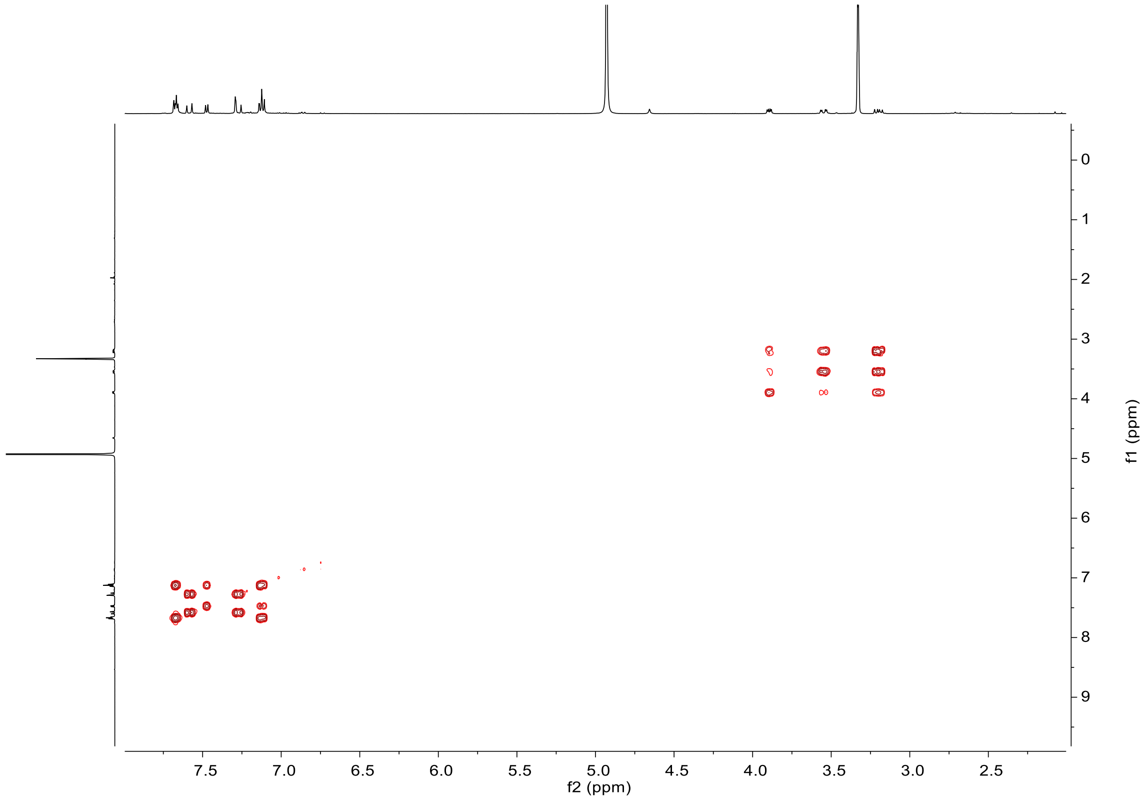

HSQC NMR (500 MHz, MeOD)

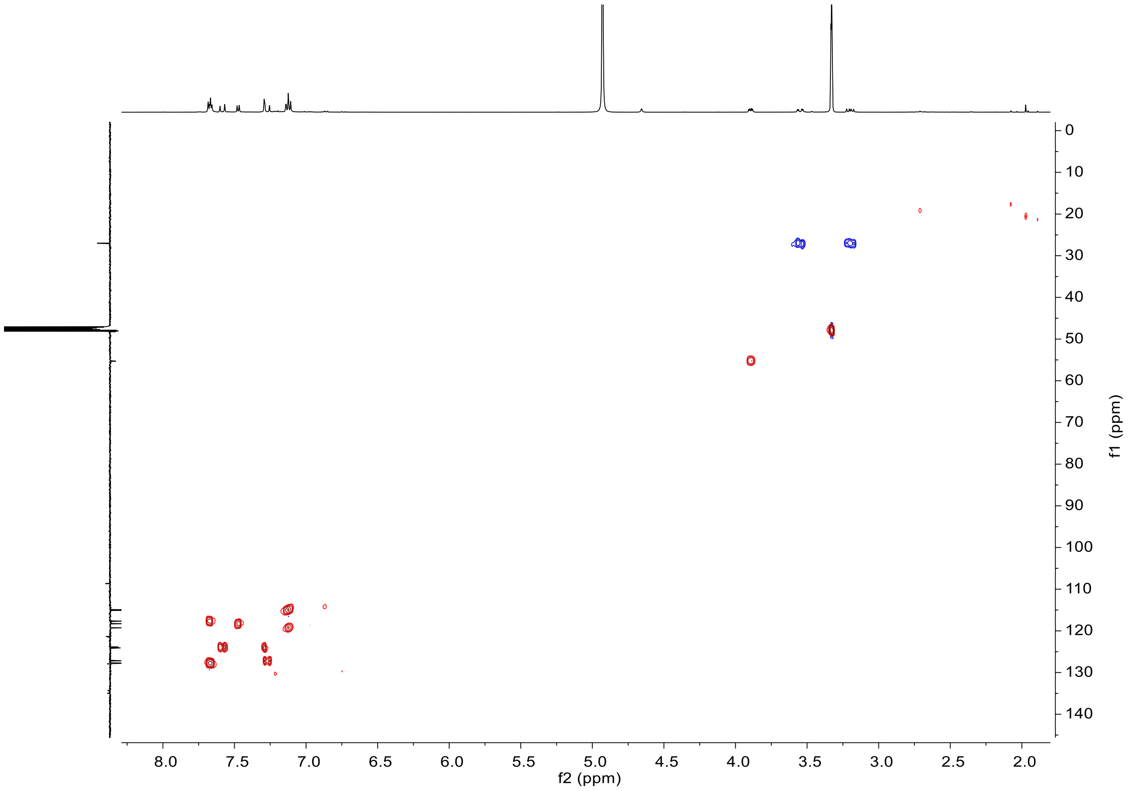

HMBC NMR (500 MHz, MeOD)

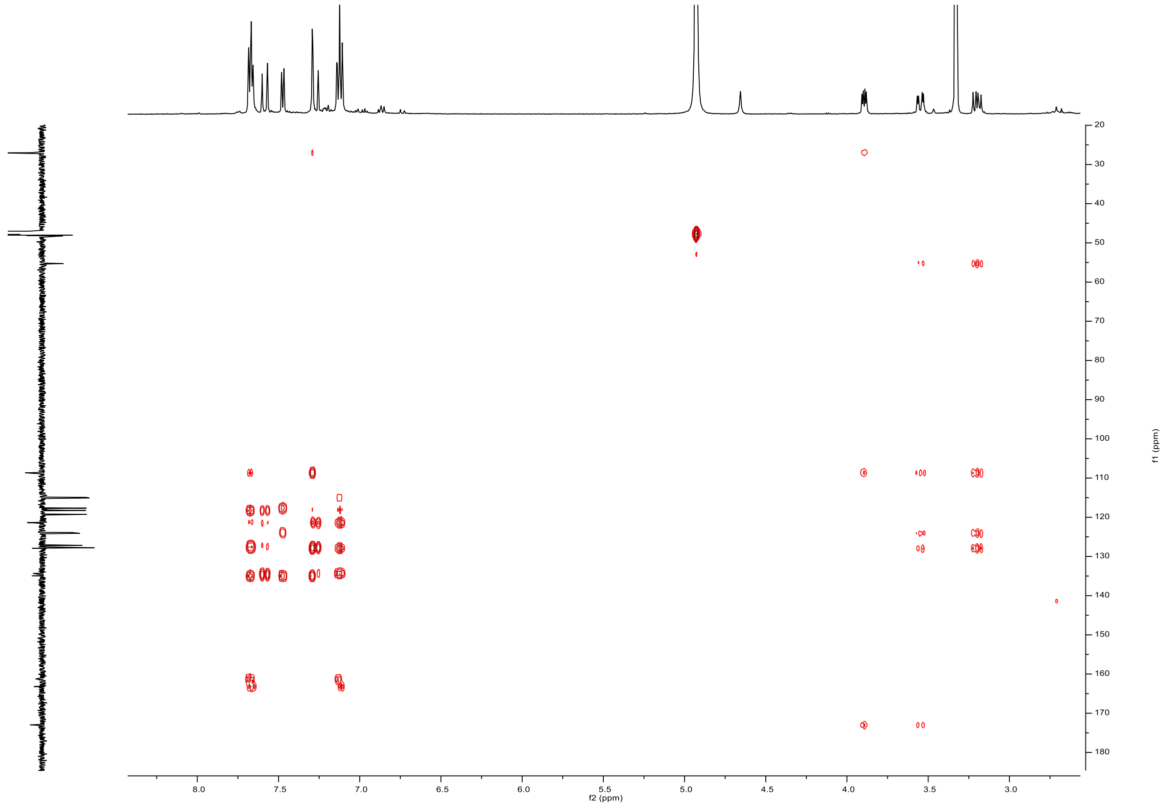

(*S*)-2-amino-3-(7-((*E*)-3-fluorostyryl)-1H-indol-3-yl)propanoic acid

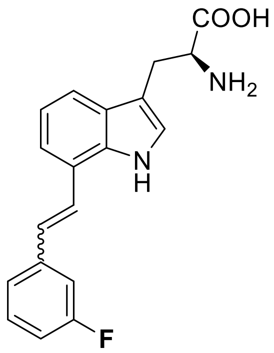

^{1}H NMR (500 MHz, MeOD)

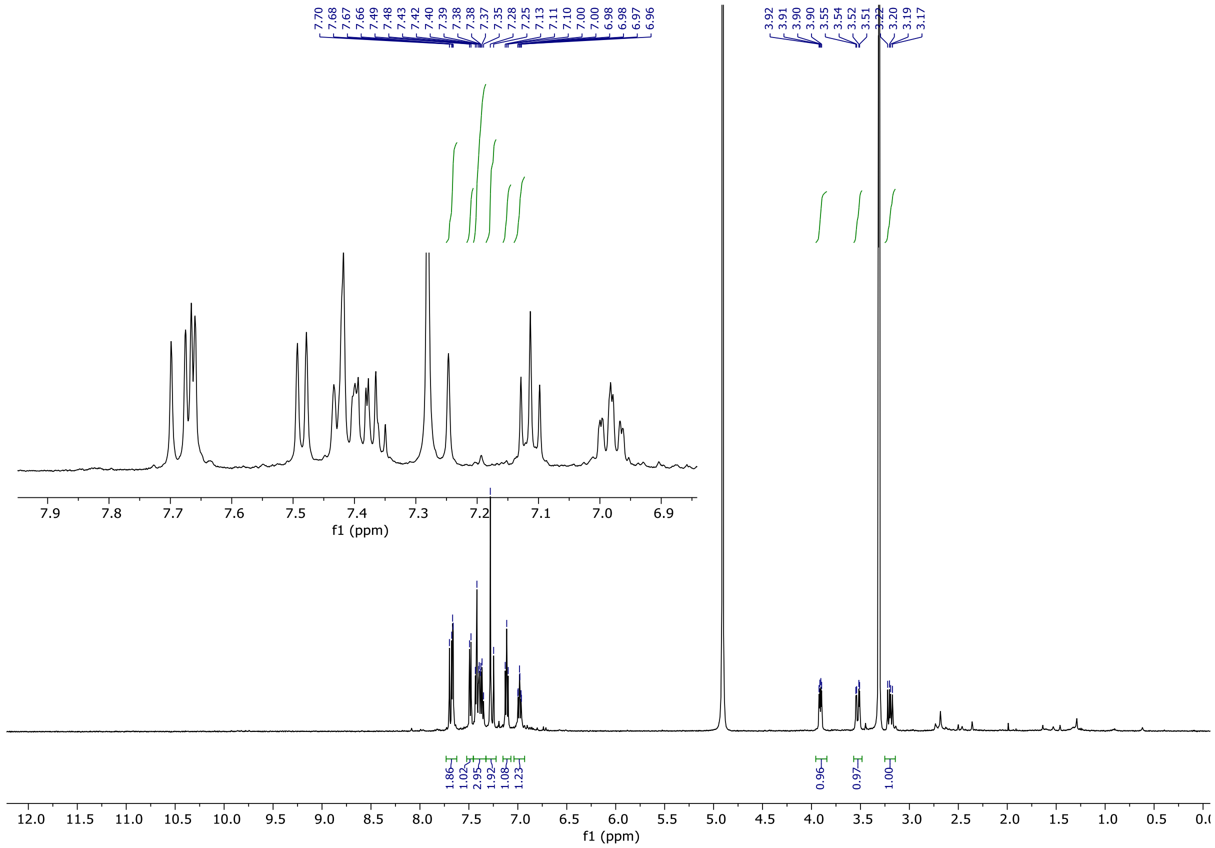

^{13}C NMR (126 MHz, MeOD)

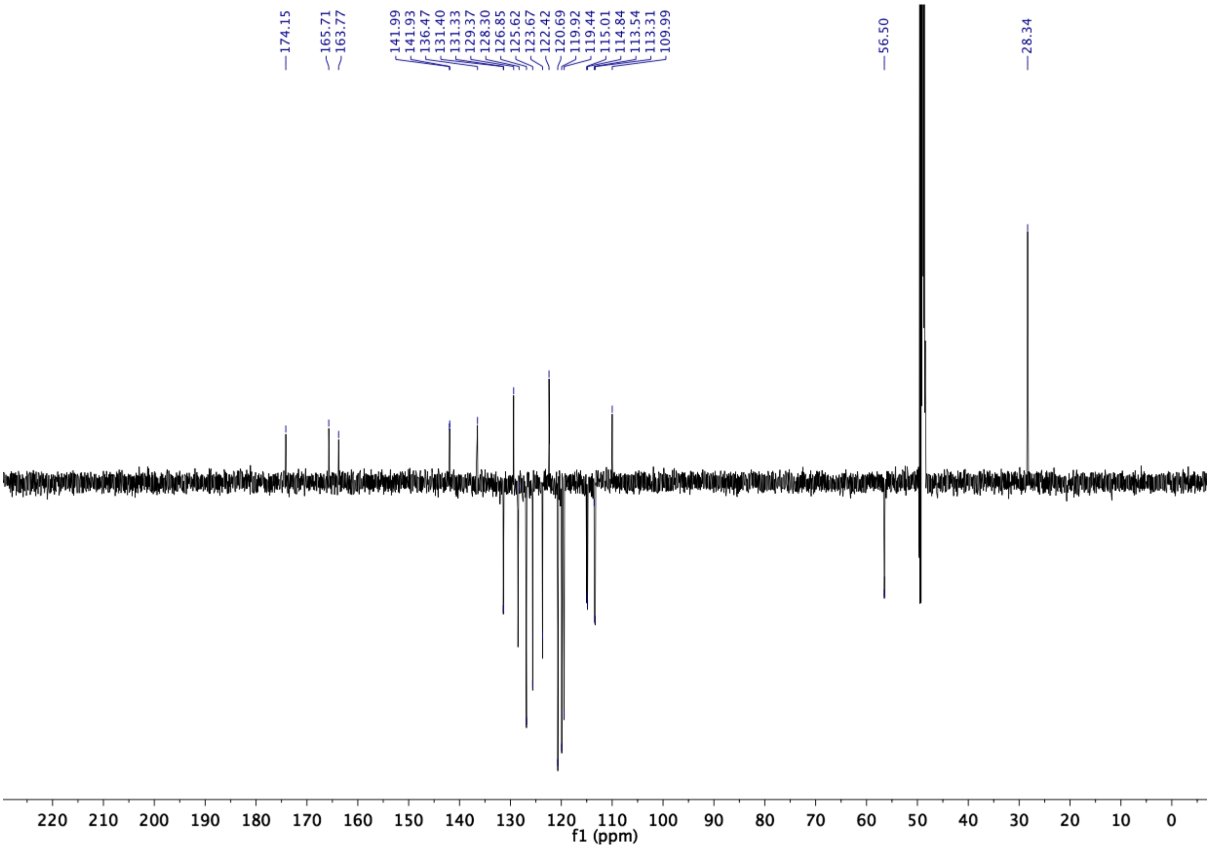

^{19}F NMR (377 MHz, DMSO-d_6)

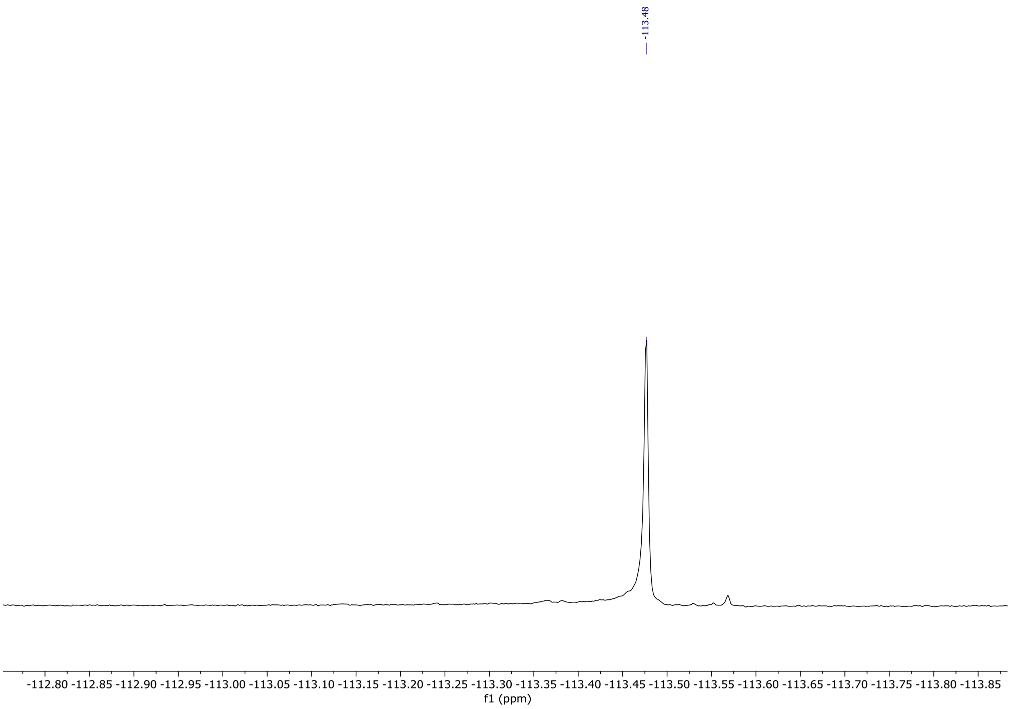

(*S*)-2-amino-3-(5-((*E*)-4-cyanostyryl)-1H-indol-3-yl)propanoic acid

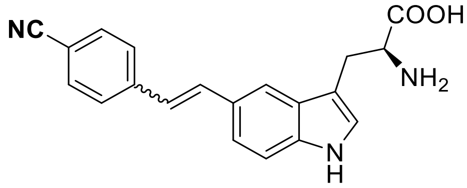

¹H NMR (500 MHz, MeOD)

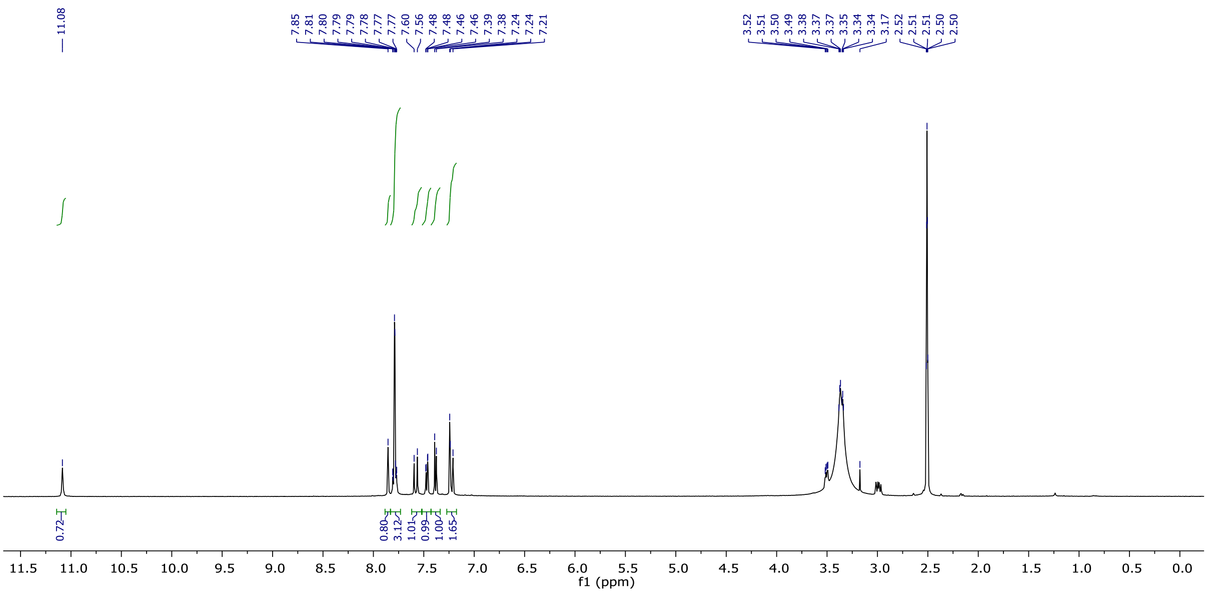

¹³C NMR (126 MHz, MeOD)

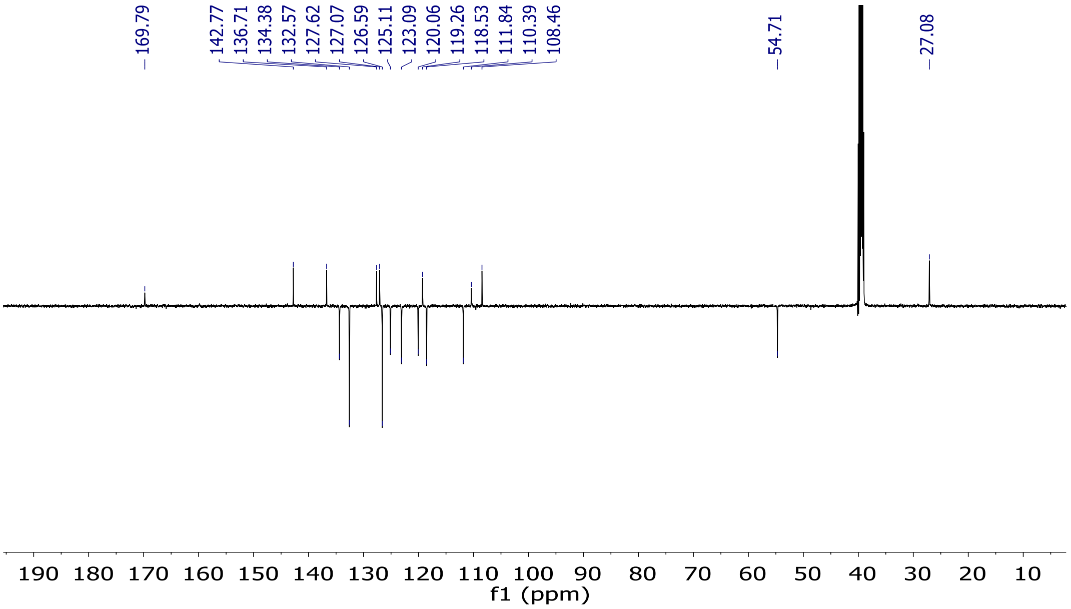

(*S*)-2-amino-3-(7-((*E*)-4-cyanostyryl)-1H-indol-3-yl)propanoic acid

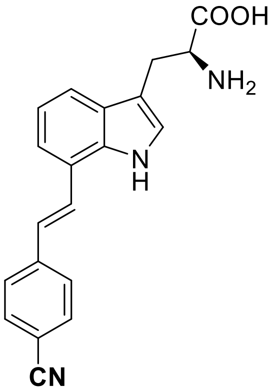

^{1}H NMR (500 MHz, MeOD)

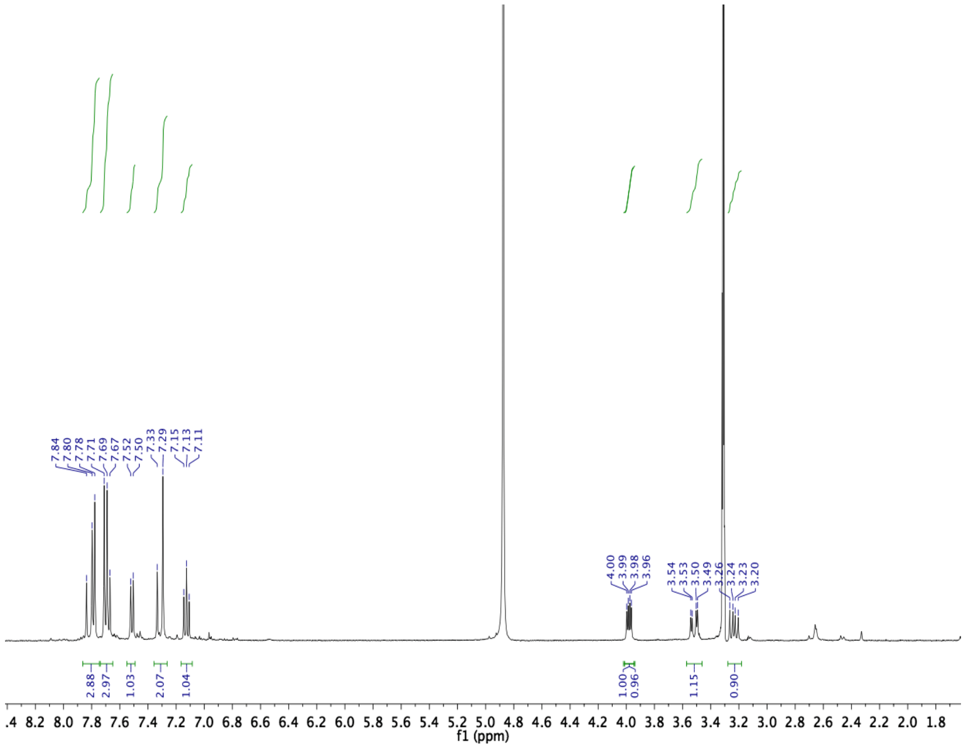

^{13}C NMR (126 MHz, MeOD)

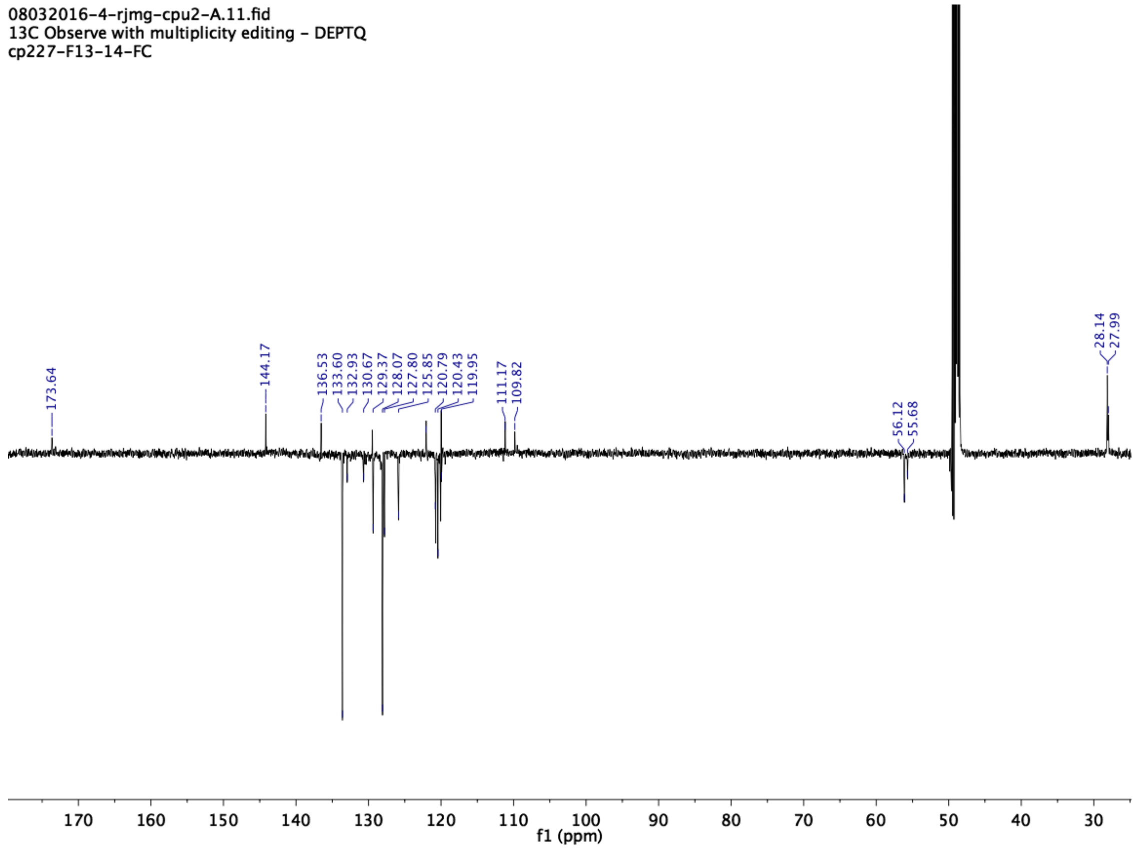

(*S*)-2-amino-3-(5-(2-(pyridin-4-yl)vinyl)-1H-indol-3-yl)propanoic acid

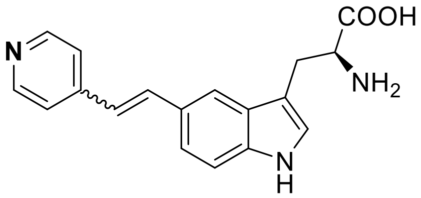

^{1}H NMR (500 MHz, MeOD)

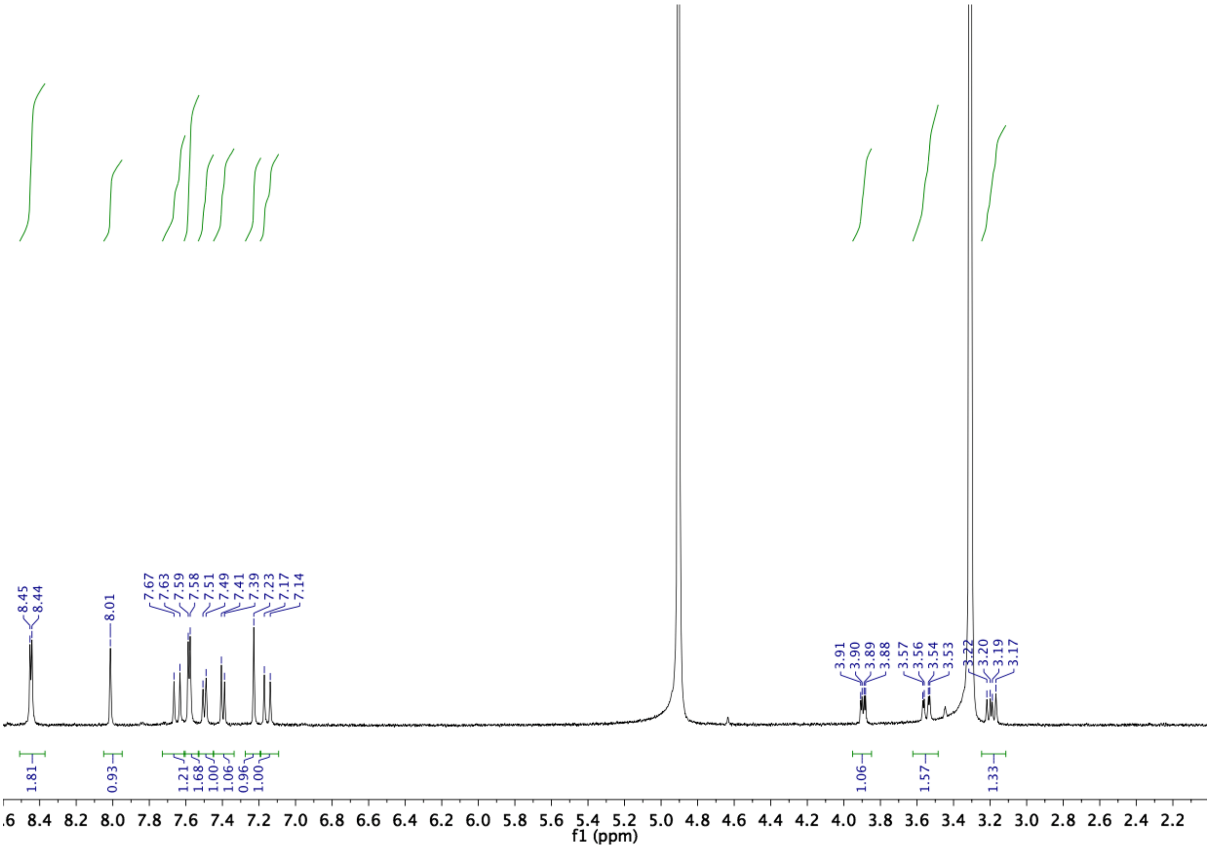

^{13}C NMR (126 MHz, MeOD)

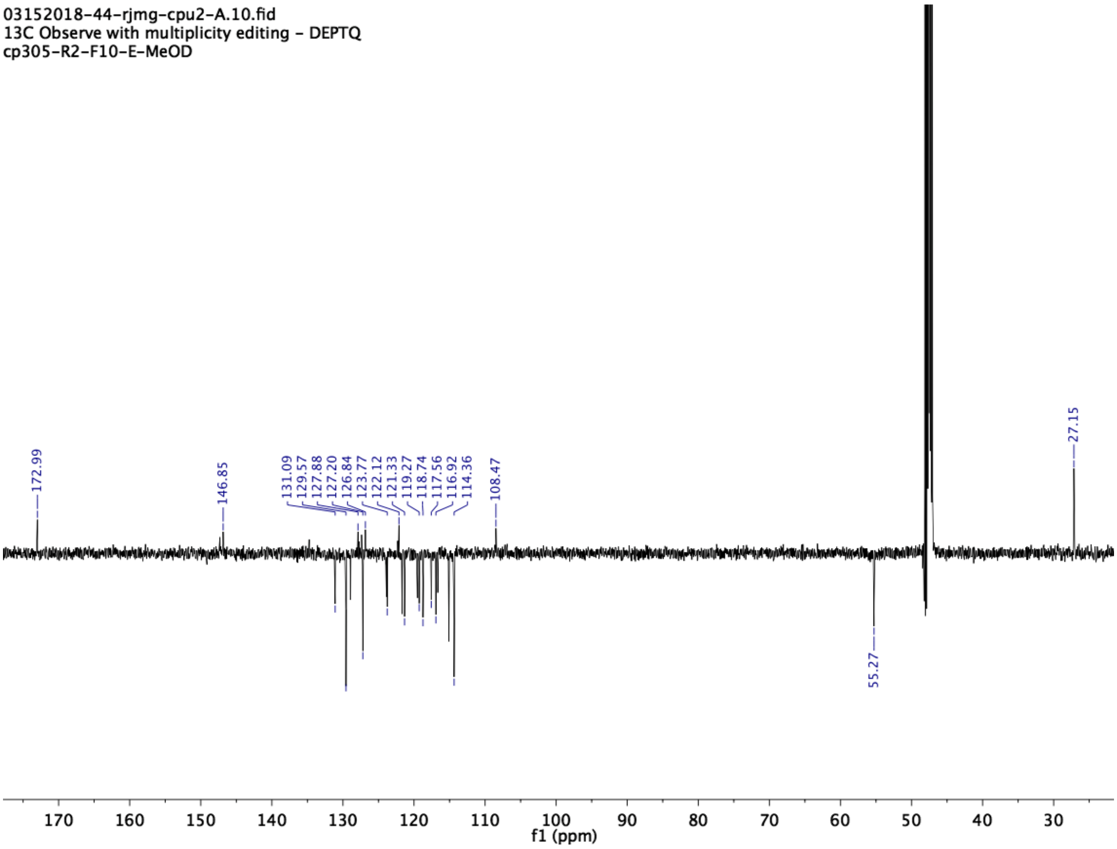

(*S*)-2-amino-3-(7-((*E*)-2-(pyridin-4-yl)vinyl)-1H-indol-3-yl)propanoic acid

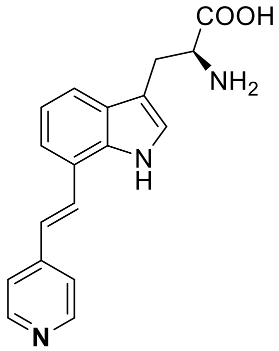

^{1}H NMR (500 MHz, MeOD)

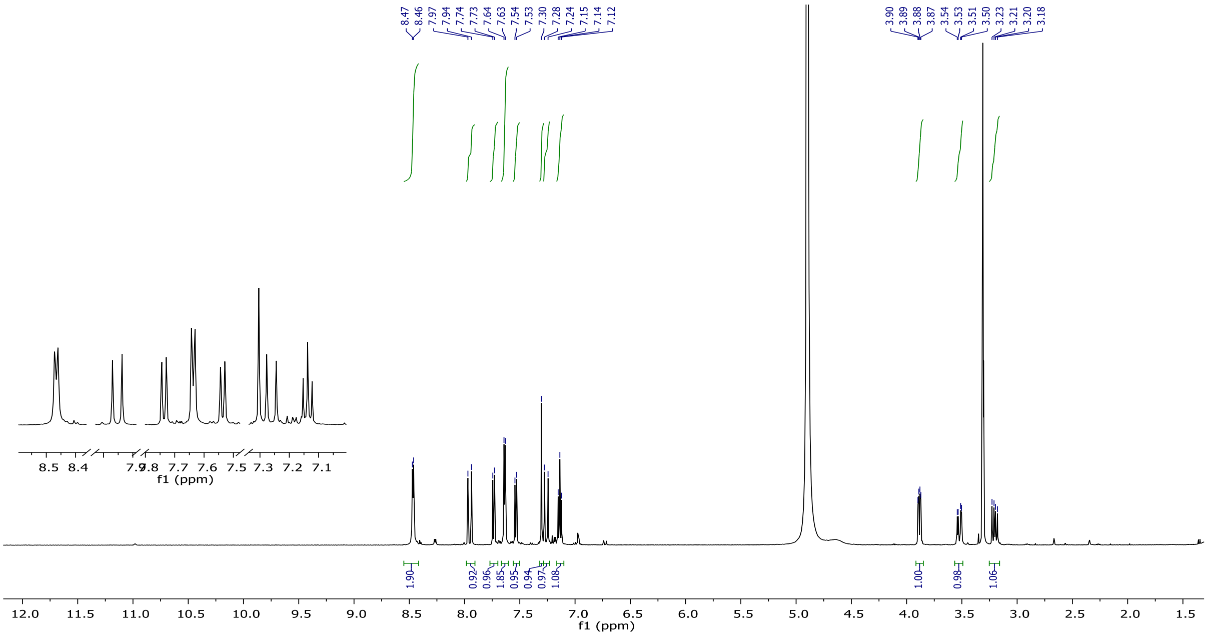

^{13}C NMR (126 MHz, MeOD)

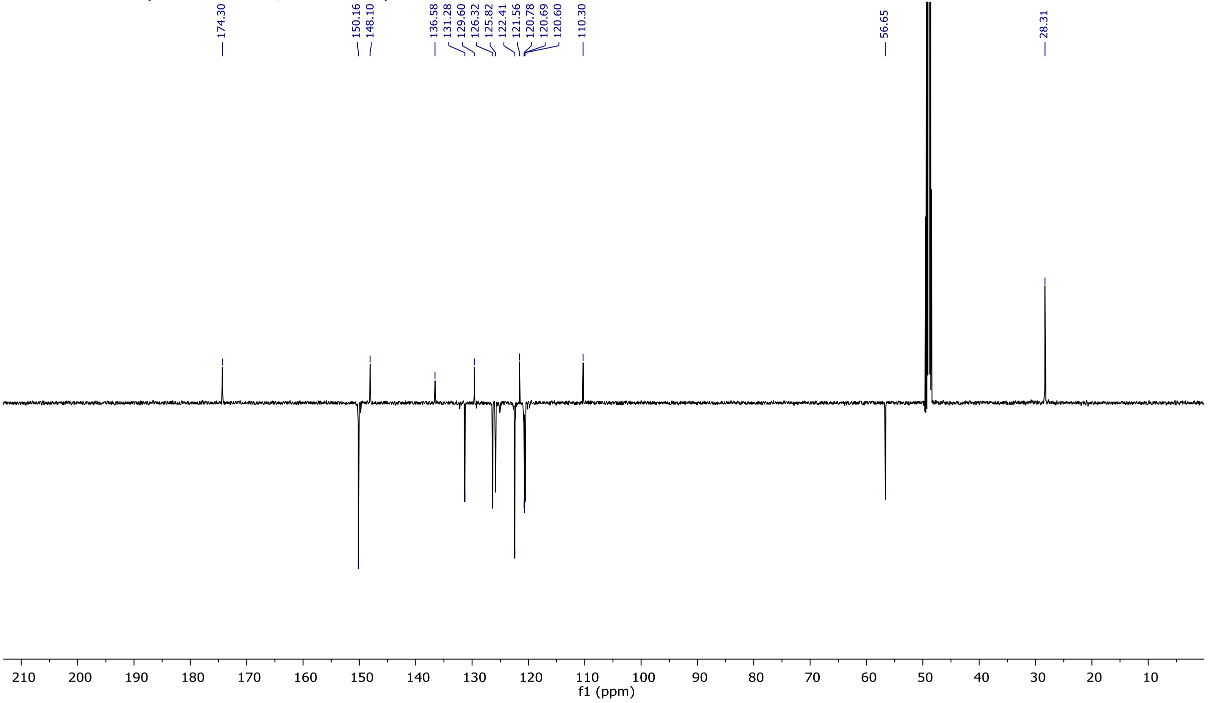

(*R*)-1-(3-(5-(((*Z*)-6-bromo-1H-indol-3-yl)methylene)-3,6-dioxopiperazin-2-yl)propyl)guanidine

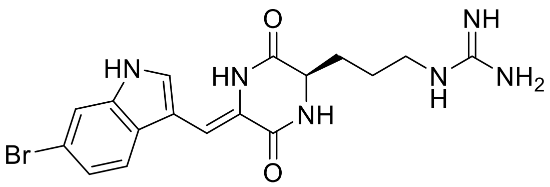

^{1}H NMR (700 MHz, MeOD)

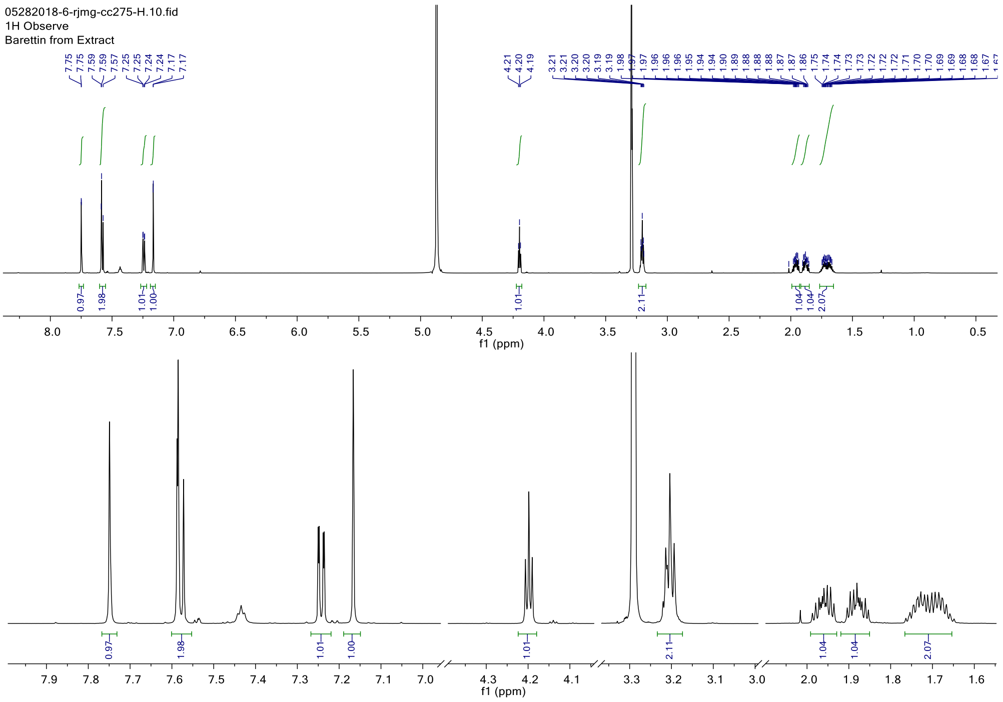

^{13}C NMR (176 MHz, MeOD)

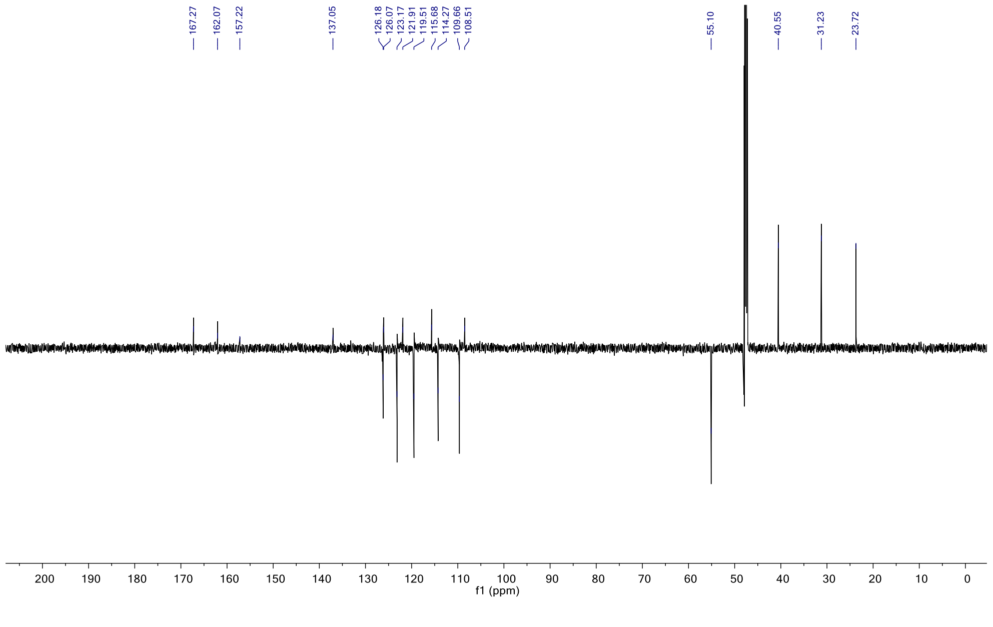

1-(3-((*R*)-5-(*Z*)-((6-(4-fluorostyryl)-1H-indol-3-yl)methylene)-3,6-dioxopiperazin-2-yl)propyl)guanidine

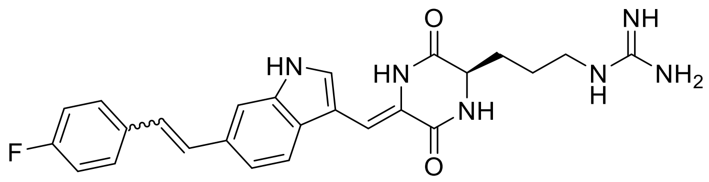

^{1}H NMR (700 MHz, MeOD)

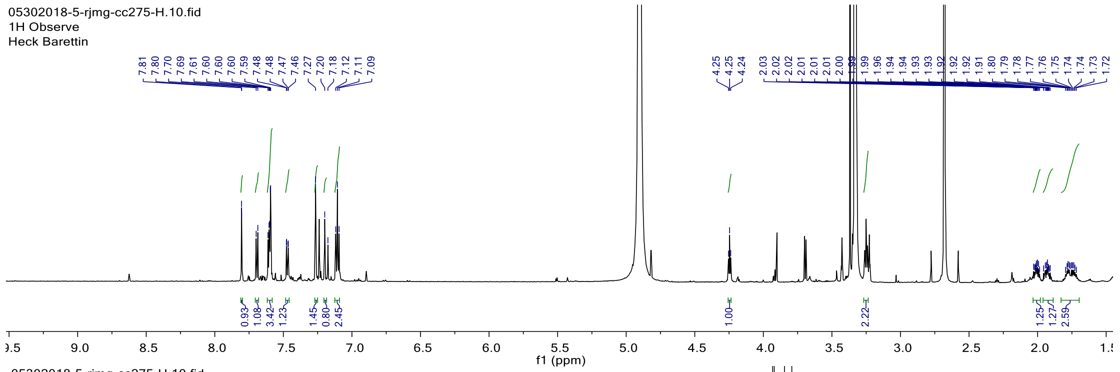

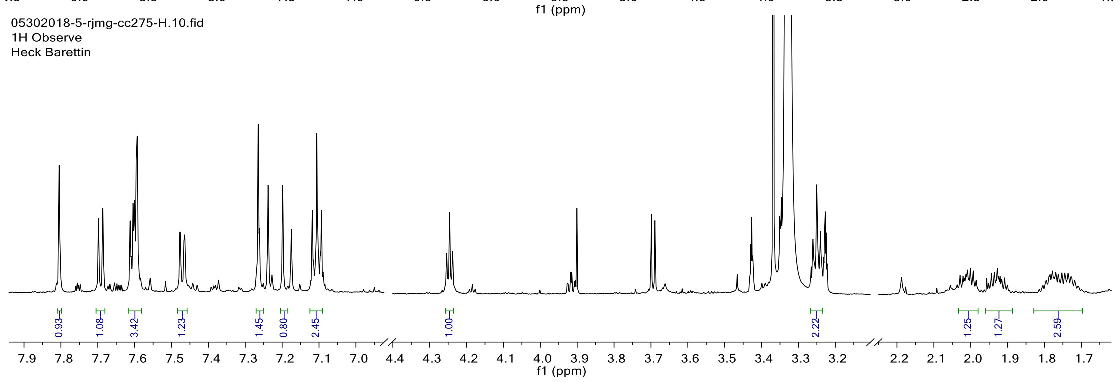

^{19}F NMR (659 MHz, MeOD)

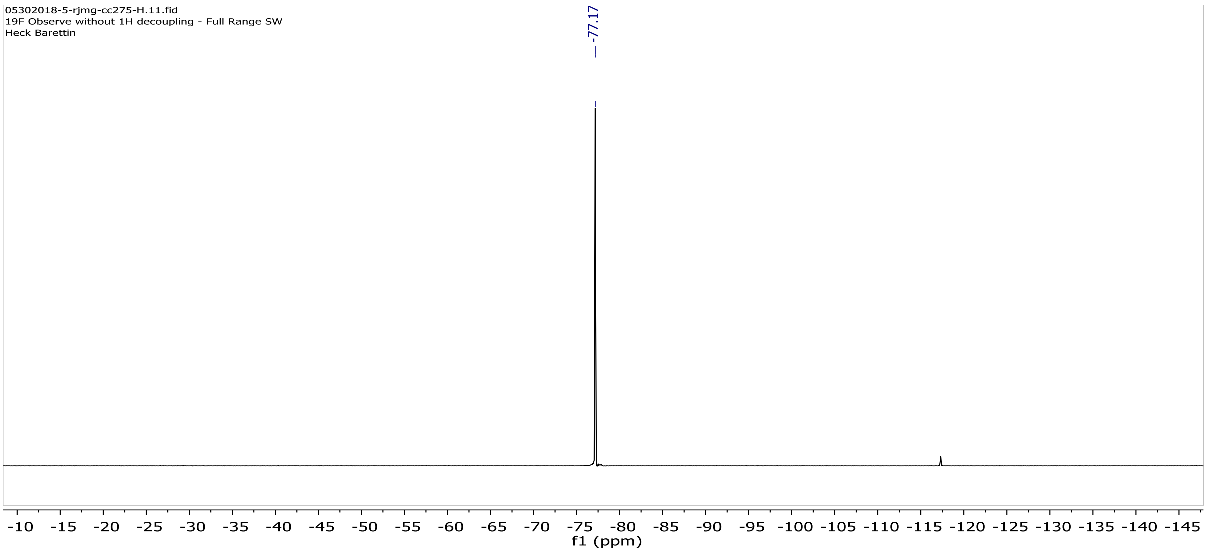

Pacidamycin D

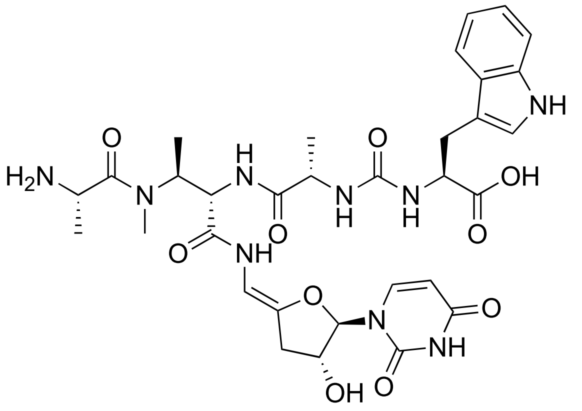

^{1}H NMR (700 MHz, MeOD)

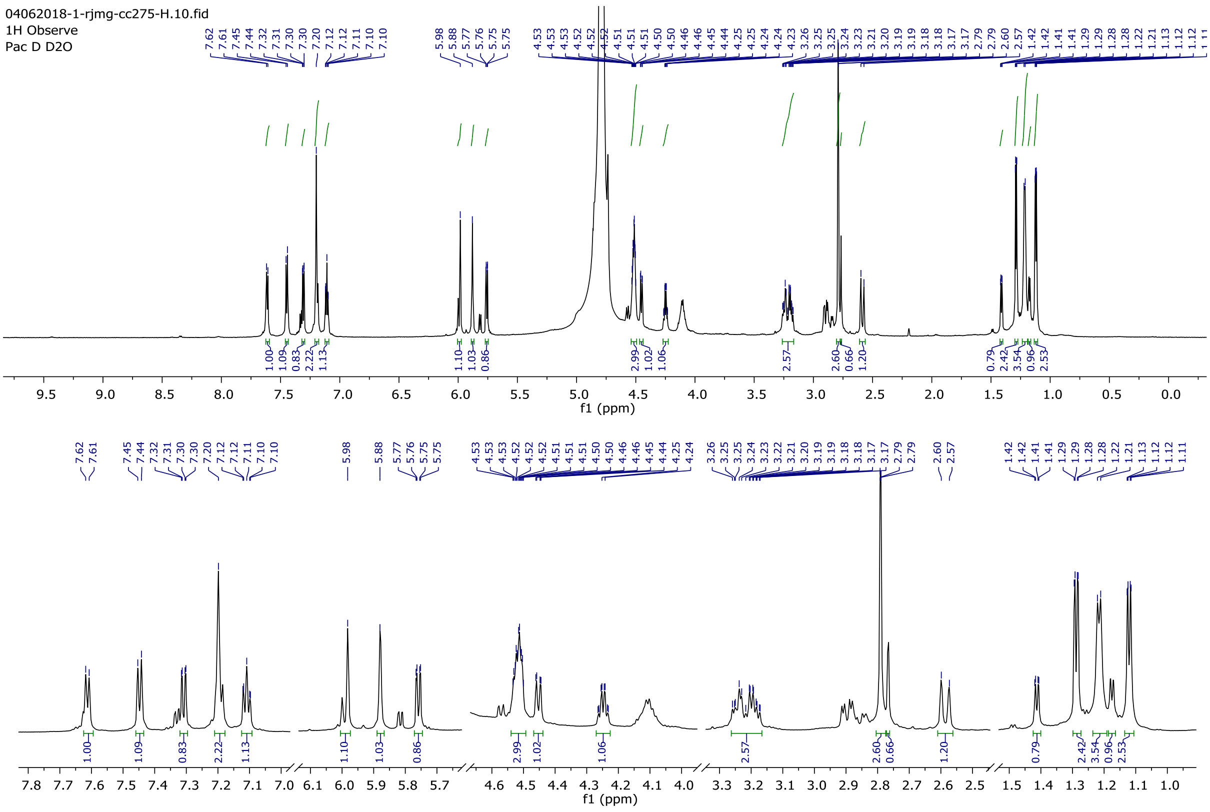

HSQC (700 MHz, MeOD)

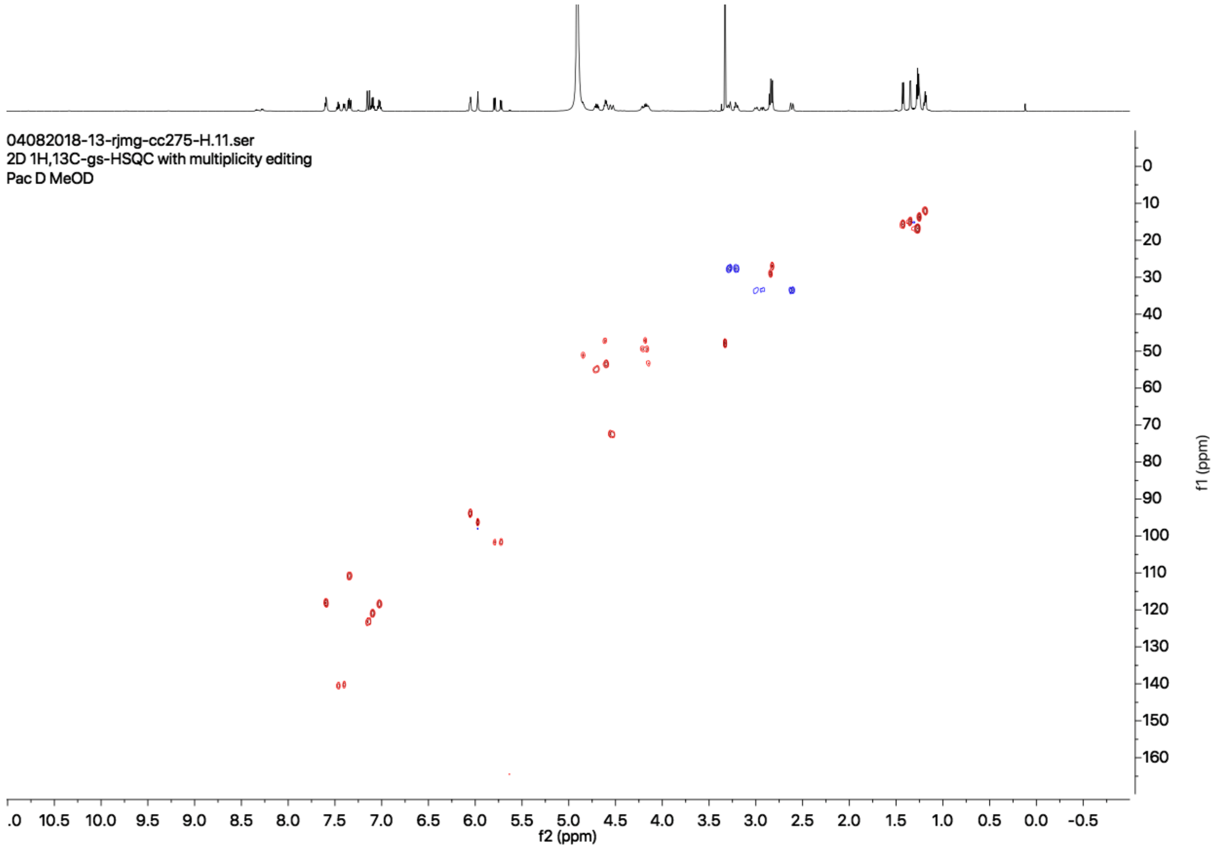

Br-pacidamycin D

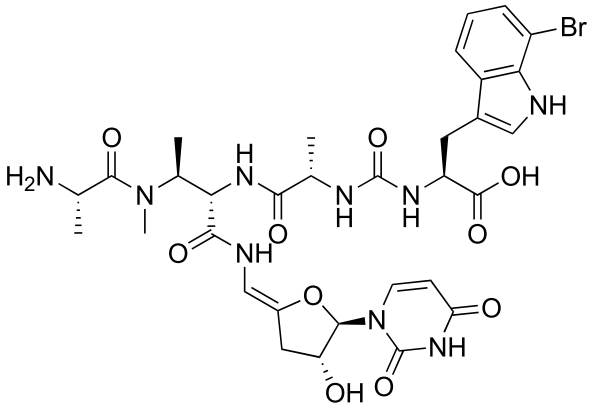

^{1}H NMR (700 MHz, MeOD)

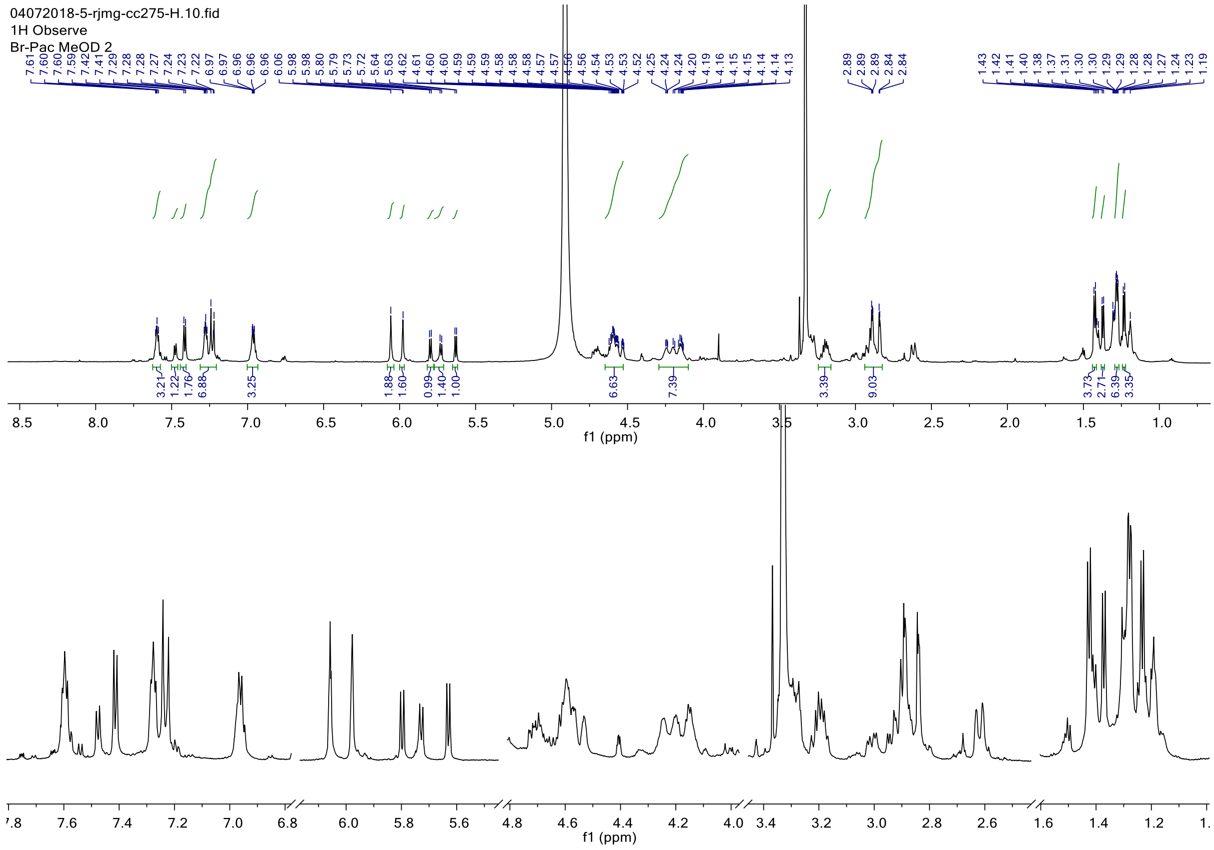

HSQC (700 MHz, MeOD)

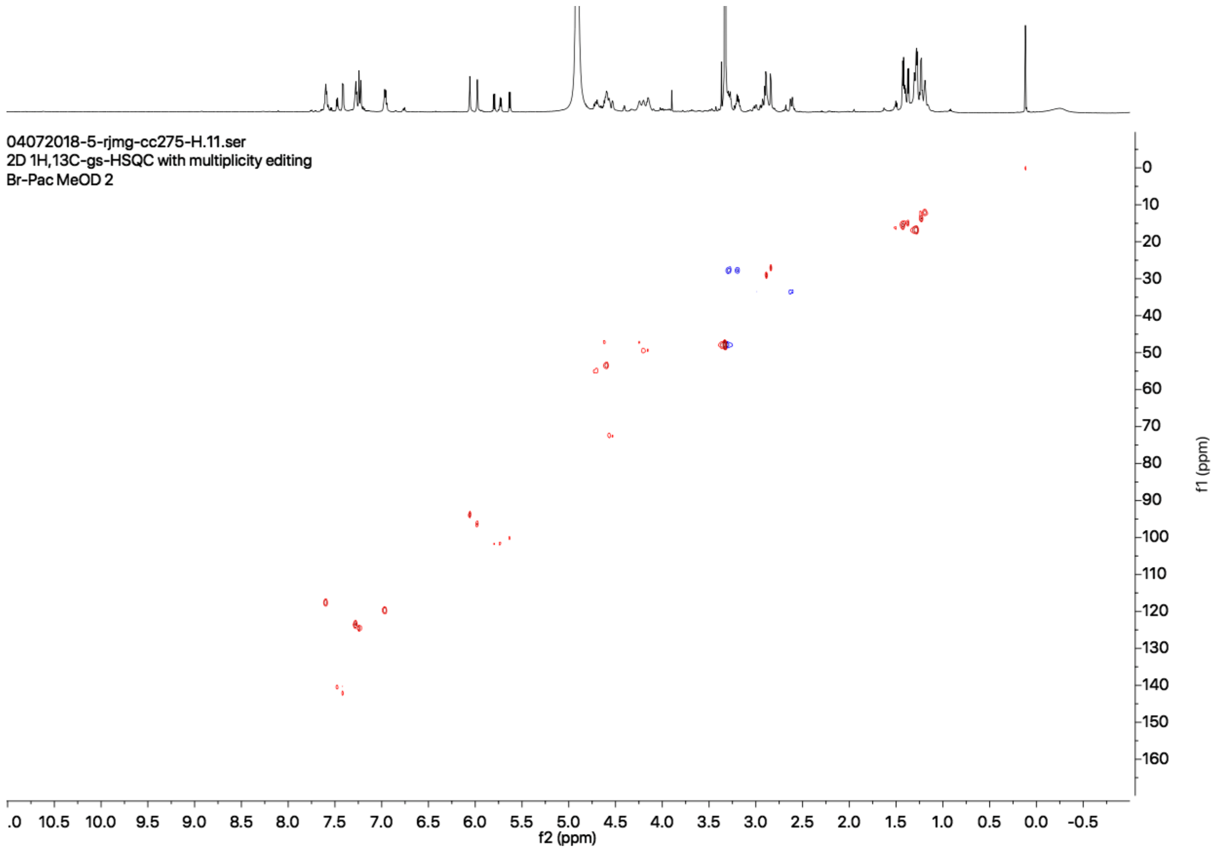

Supplement: Supplementary file 1 — Supplementary [file CHEM-25-10866-s001.pdf]
